# Dual-action mitochondria-targeted prodrugs that both deplete mitochondrial glutathione and deliver a toxic payload to the matrix

**DOI:** 10.1016/j.ejmech.2026.118706

**Published:** 2026-04-15

**Authors:** Patrick A. Cardwell, Alva M. Casey, Suvagata Roy Chowdhury, Eloïse Marques, Chak Shun Yu, Rebecca L. Taig, Stuart T. Caldwell, Julien Prudent, Michael P. Murphy, Richard C. Hartley

**Affiliations:** a School of Chemistry, Joseph Black Building, University Avenue, University of Glasgow, Glasgow, G12 8QQ, UK; b MRC Mitochondrial Biology Unit, University of Cambridge, Cambridge Biomedical Campus, Cambridge, CB2 0XY, UK; c Department of Medicine, University of Cambridge, Cambridge, CB2 0QQ, UK

**Keywords:** Mitochondria, Glutathione, Prodrug, Reactive oxygen species, Oxidative stress, Cancer

## Abstract

Mitochondrial glutathione (mGSH) protects the organelle and the cell against reactive oxygen species (ROS), electrophilic metabolites and xenobiotics. Many cancers upregulate GSH to confer resistance against cell death by ferroptosis and anticancer drugs, so mGSH depletion is a potential anticancer strategy. We previously developed MitoCDNB, a mitochondria-targeted molecule that selectively depletes mGSH and disrupts mitochondrial thiol redox homeostasis. However, mGSH depletion by MitoCDNB required catalysis by glutathione-S-transferases (GSTs). Here, we develop a dual-action prodrug scaffold to deplete mGSH independently of GSTs and simultaneously release a payload to increase oxidative stress. The scaffold has four components: a triphenylphosphonium (TPP) group for targeting to the mitochondria, a GSH-reactive electrophilic dinitroaryl ring bearing a sulfonamide leaving group for depleting mGSH, an ethylenediamine-derived self-immolative linker and a phenolic payload. The rates of nucleophilic aromatic substitution (S_N_Ar) of the sulfonamide by GSH and the cyclisation of the released linker-payload intermediate were measured and the kinetics successfully modelled as consecutive reactions. Under physiological levels of GSH (10 mM) and matrix pH (8.0), our best linker releases a 7-hydroxycoumarin reporter with a half-life of 2.5 min at 30 °C. We used the scaffold for cellular and mitochondrial uptake of a compound that depletes mGSH and releases the redox-cycling pro-oxidant, menadiol/menadione, in the mitochondrial matrix. The combination of mGSH depletion with enhanced mitochondrial ROS production showed synergistic cytotoxicity towards cancer cells, paving the way for the development of dual-action mitochondria-targeted prodrugs as potential cancer therapeutics.

## Highlights

• Dual-action prodrugs are activated by glutathione to release a phenolic payload.
• The prodrugs deplete glutathione in mitochondria via formation of a conjugate.
• The prodrugs selectively deliver the payload to the mitochondria in cells.
• This system delivers the ROS-generating drug, menadiol, to mitochondria.
• Glutathione depletion and ROS generation showed synergistic cytotoxicity.

## Graphical abstract

**Figure:**
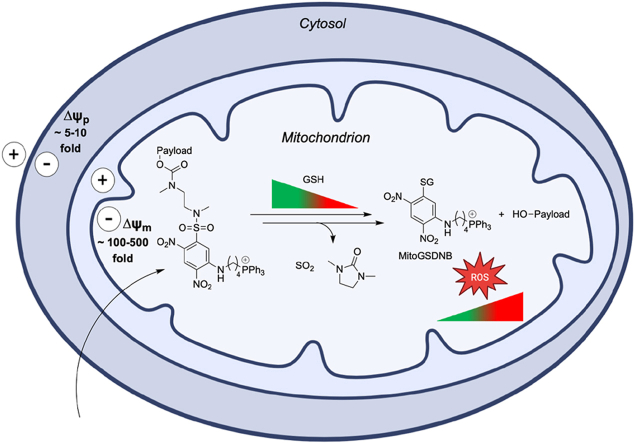

## 1 Introduction

Mitochondrial thiol redox homeostasis is central to cellular health and function, with mitochondrial glutathione (mGSH) serving as a critical defence against oxidative damage and xenobiotic toxicity [1,2]. Synthesised in the cytosol and transported into mitochondria [3,4], mGSH scavenges reactive oxygen species (ROS), electrophilic metabolites and xenobiotics by several mechanisms [1]. The glutathione (GSH)/glutathione disulfide (GSSG) ratio of the mGSH pool is maintained high by the action of glutathione reductase [1], which couples the reduction of mGSSG to mGSH with the oxidation of NADPH to NADP^+^ [1]. The concentration of mGSH in mammalian cells ranges from 1 to 10 mM depending on the tissue [1,5]. A major detoxification mechanism by mGSH uses glutathione-S-transferases (GSTs), which catalyse the reaction of GSH with electrophiles to form glutathione conjugates (GS conjugates) that are then metabolised and excreted [[5], [6], [7], [8]]. Cancer cells often exhibit elevated levels of ROS, which is counterbalanced by upregulated antioxidant defences, including GSH [8] and GSTs [9]. Elevated GSH levels are found in several tumour types, including breast, lung, colon, and ovarian, promoting their growth and protecting against cell death pathways such as ferroptosis [[10], [11], [12], [13], [14]]. The upregulation of GSH and GSTs also contributes to resistance to chemotherapeutic drugs, such as cisplatin [15], doxorubicin [12,16], busulfan [17], and dichloroacetate [18], via GST-catalysed GSH conjugation. GSH overproduction also limits the effectiveness of ROS-based therapies [19].

**Fig. 1:**
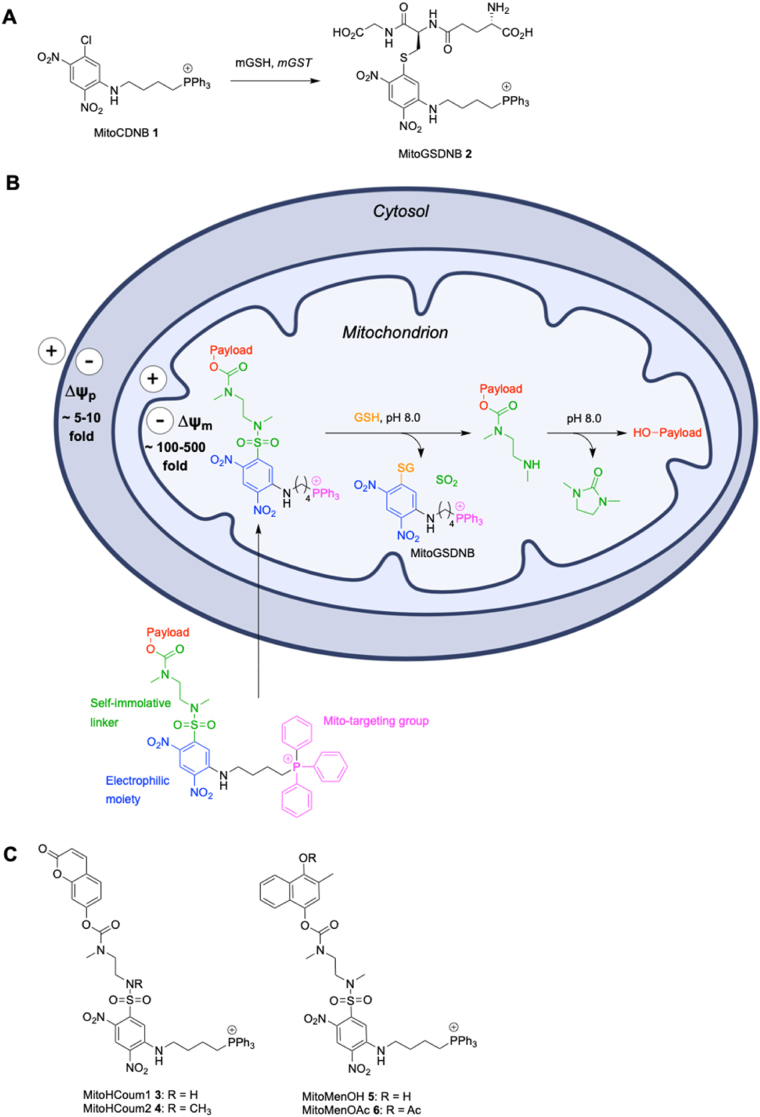
(A) MitoCDNB depletes mitochondrial GSH (mGSH) through conjugation to GSH catalysed by mitochondrial GST (mGST) catalysis. (B) Dual-action prodrug strategy to both deplete mGSH and release a hydroxy-bearing payload. (C) Dual-action mitochondria-targeted prodrugs: MitoHCoum1 **3** and MitoHCoum2 **4** designed to release 7-hydroxycoumarin, while MitoMenOH **5** and MitoMenOAc **6** are designed to release menadiol.

Since mitochondria are an important site of ROS generation, selective depletion of mGSH should sensitise cancer cells to oxidative damage and cell death. In addition, the mGSH pool originates in the cytosol, and import of GSH into mitochondria is slow, meaning targeting mGSH for depletion may be more effective to disrupt cell survival than targeting the cytosolic pool [20,21]. Depleting the mGSH pool can be achieved by using a lipophilic cation to target an electrophilic compound to the mitochondria that then reacts with mGSH to form a GS conjugate. Previously, we developed MitoCDNB **1** (Fig. 1A), a mitochondria-targeted GST-substrate molecule that selectively depletes mGSH and disrupts mitochondrial thioredoxin redox homeostasis in cells and *in vivo* [2]. The lipophilic triphenylphosphonium (TPP) cation drives accumulation of MitoCDNB **1** in the mitochondrial matrix in response to the large negative membrane potential [2]. In the matrix, MitoCDNB **1** reacts with mGSH by GST catalysis to produce the GS-conjugate MitoGSDNB **2**, which is then excreted [2].

Building on this approach, here we seek to design a dual-action mitochondria-targeted prodrug system that combines mGSH depletion independent of GST activity, with the simultaneous release of a ROS-generating phenolic payload. A prodrug that both consumes mGSH and enhances ROS production should enhance cytotoxicity. Additionally, consumption of mGSH should reduce drug detoxification by mGSH. Previously, GST-activated prodrugs of doxorubicin [22] and metformin [23] have been developed where the drug is attached to an electron-poor aromatic ring via a sulfonamide group. However, these prodrugs lacked mitochondria-targeting and required GST catalysis for rapid reaction with GSH. Our dual-action prodrug scaffold has four components; a TPP group for mitochondrial targeting, a mGSH-reactive electrophilic dinitroaryl ring armed with a sulfonamide leaving group, an ethylenediamine-derived self-immolative linker and a phenolic payload (Fig. 1B). To develop prodrugs that would be highly reactive with mGSH without the need for GST catalysis, we first considered attaching the phenolic payload to the electron-poor 2,4-dinitroaryl ring of MitoCDNB **1** via the sulfur of a sulfonate ester. However, the sulfonate ester derivative was unstable. To overcome this, we switched from a sulfonate to a less reactive sulfonamide and included an ethylenediamine linker between the sulfonamide moiety and the carbamate of the phenolic payload. Considering the σ_m_ Hammett parameters as indicative of a group's inductive effect [24], and by extension its propensity for *ipso*-substitution by an S_N_Ar reaction [25], we reasoned that the sulfonamide moiety is more electron-withdrawing than the chloride substituent in MitoCDNB **1**, which should enhance reactivity with mGSH in the absence of GSTs [6]. Reaction of the prodrug with mGSH should produce MitoGSDNB **2**, sulfur dioxide (SO_2_) and the ethylenediamine carbamate-linked payload. High levels of SO_2_ are widely known to cause oxidative damage in cells [26], potentially providing additional toxicity. Once freed from the 2,4-dinitroaryl group by mGSH, the ethylenediamine carbamate-linked payload should undergo intramolecular cyclisation, promoted by the alkaline conditions of the mitochondrial matrix, to release the active payload and a cyclic urea by-product. Cyclisation of an ethylenediamine-derived carbamate is a widely-used self-immolative strategy to release hydroxy-bearing payloads [[27], [28], [29], [30], [31]]. Furthermore, the nitrogen atoms of the linker can be alkylated with various substituents to tune the rate of cyclisation.

To assess this delivery strategy, we first prepared MitoHCoum1 **3** and MitoHCoum2 **4** (Fig. 1C), which should react with mGSH and then release fluorescent 7-hydroxycoumarin. We also prepared a prodrug, MitoMenOH **5**, designed to both deplete mGSH and release the redox cycler menadiol. Menadiol is oxidised by O_2_ to menadione, producing superoxide (O_2_^•-^), which induces apoptosis [[32], [33], [34], [35]], making it an attractive drug to enhance ROS production. We extended this by preparation of MitoMenOAc **6** (Fig. 1C) in which the menadiol was acetylated, necessitating esterase-catalysed hydrolysis to release the active drug, thus providing a further level of potential modulation of efficacy. Here we evaluated the uptake, reactivity and toxicity of these dual-action prodrugs in isolated mitochondria and in cells. This dual-action system provides a versatile platform for depleting mGSH and enhancing oxidative damage by delivering phenolic payloads to the mitochondria within cells.

## 2 Results

### 2.1 Preparation of prodrugs and controls

**Scheme 1:**
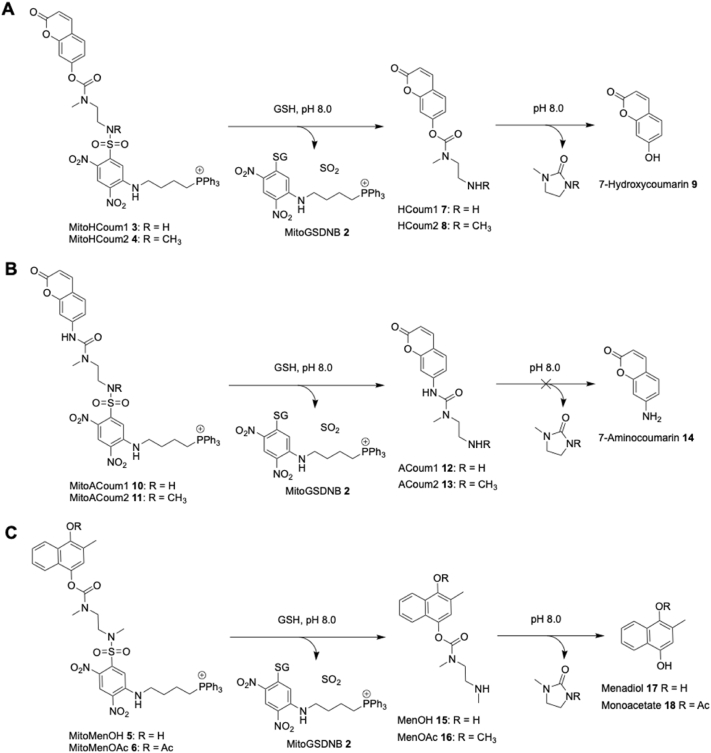
Predicted reactivity of prodrugs and analogues. (A) MitoHCoum1 **3** and MitoHCoum2 **4** react with GSH to generate MitoGSDNB **2** and HCoum1 **7** or HCoum2 **8**, respectively, which in turn release 7-hydroxycoumarin **9** under alkaline conditions. (B) MitoACoum1 **10** and MitoACoum2 **11** react with GSH to generate MitoGSDNB **2** and ACoum1 **12** or ACoum2 **13**, respectively, which are stable to release of 7-aminocoumarin **14** under alkaline conditions. (C) MitoMenOH **5** and MitoMenOAc **6** react with GSH to generate MitoGSDNB **2** and MenOH **15** or MenOAc **16**, respectively, which in turn release menadiol **17** or the monoacetate **18**, respectively, under alkaline conditions.

To assess our dual-action prodrug strategy, we prepared MitoHCoum1 **3** and MitoHCoum2 **4** (Scheme 1A). The attachment of the labile sulfonamide moiety to the highly electron-poor 2,4-dinitrobenzene ring in these compounds should greatly increase the rate of reaction with GSH in the absence of GST catalysis. MitoHCoum1 **3** and MitoHCoum2 **4** should react with GSH to produce MitoGSDNB **2** and the ethylenediamine carbamate intermediates, HCoum1 **7** and HCoum2 **8**, respectively. At mitochondrial matrix pH 8.0, these self-immolative intermediates are predicted to rapidly cyclise and release the payload 7-hydroxycoumarin **9**. To isolate the putative GSH activation step and study its kinetics, we prepared the control compounds MitoACoum1 **10** and MitoACoum2 **11** (Scheme 1B). The ethylenediamine linker only releases hydroxy-bearing payloads [[27], [28], [29], [30], [31]], meaning it should be stable when linked to an amino group. Hence, MitoACoum1 **10** and MitoACoum2 **11** should react with GSH to form the stable compounds ACoum1 **12** and ACoum2 **13**, respectively, and 7-aminocoumarin **14** should not be released. To study the kinetics of the cyclisation step, we prepared the protonated forms of HCoum1 **7**, HCoum2 **8**, ACoum1 **12** and ACoum2 **13**. MitoMenOH **5** and its acetylated analogue, MitoMenOAc **6**, were designed as prodrugs of menadiol **17** (Scheme 1C). These prodrugs were expected to react with GSH to form the ethylenediamine carbamate derivatives MenOH **15** and MenOAc **16**, respectively, which in turn would release menadiol **17** and the monoacetate **18**, respectively. In mitochondria, menadiol **17** should undergo redox cycling to generate O_2_^•-^. In mitochondria, MitoMenOAc **6**, its corresponding intermediate MenOAc **16** or corresponding product monoacetate **18**, should be hydrolysed by esterases to ultimately produce menadiol **17**.

**Scheme 2:**
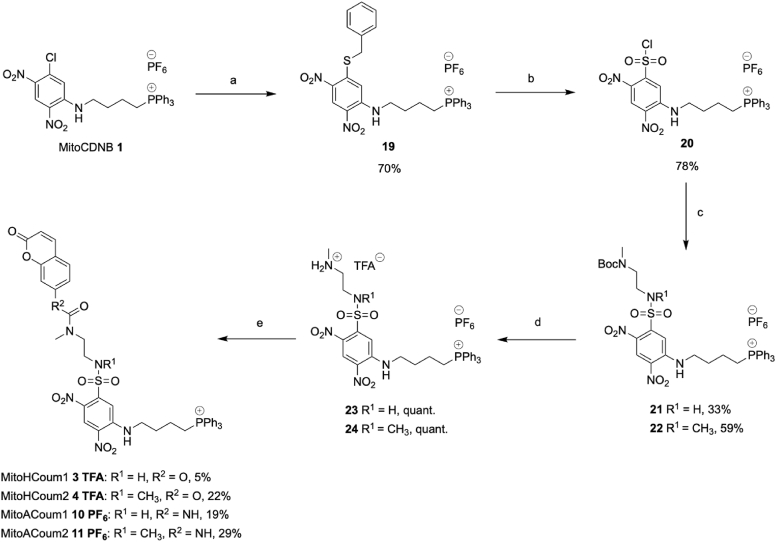
Synthesis of MitoHCoum1 **3**, MitoHCoum2 **4**, MitoACoum1 **10** and MitoACoum2 **11**. a: BnSH, EtN^i^Pr_2_, MeCN, reflux, 24 h. b: 1,3-Dichloro-5,5-dimethylhydantoin, MeCN, AcOH, H_2_O, RT, 24 h. c: Hexyl-1-amine, NEt_3_, DCM, RT, 3 h. d: H_2_N(CH_2_)_2_ N(Me)Boc or MeNH(CH_2_)_2_N(Me)Boc, NEt_3_, DCM, RT, 3h. e: TFA, DCM, RT, 1 h. f: (i) 7-Aminocoumarin **14** or 7-hydroxycoumarin **9**, triphosgene, EtN^i^Pr_2_, RT, 1 h (ii) **23** or **24**, EtN^i^Pr_2_, DCM, RT, 24 h.

MitoHCoum1 **3**, MitoHCoum2 **4**, MitoACoum1 **10** and MitoACoum2 **11** were prepared from MitoCDNB **1** (Scheme 2), which was synthesised as described previously [2]. MitoCDNB **1** was reacted with benzyl mercaptan to obtain sulfide **19** in good yield. Oxidative chlorination of sulfide **19** with 1,3-dichloro-5,5-dimethylhydantoin afforded sulfonyl chloride **20** as the hexafluorophosphate salt in good yield. Sulfonyl chloride **20** was coupled to *N*-Boc-*N*-methylethylenediamine or *N*-Boc-*N*,*N′*-dimethylethylenediamine, which afforded sulfonamides **21** and **22**, respectively. Boc-deprotection of sulfonamides **21** and **22** with trifluoroacetic acid afforded the ammonium compounds **23** and **24**, respectively, as the double hexafluorophosphate trifluoroacetate salts in quantitative yield. Reaction of 7-hydroxycoumarin **9** with triphosgene, followed by addition of ammonium salt **23** or **24**, produced MitoHCoum1 **3** and MitoHCoum2 **4** respectively. These were purified by semi-preparative RP-HPLC and isolated as the trifluoroacetate salts, albeit in poor yield. Reaction of 7-aminocoumarin **14** with triphosgene, followed by addition of ammonium salt **23** or **24** in the presence of base, produced MitoACoum1 **9** and MitoACoum2 **10**, respectively, as hexafluorophosphate salts, also in poor yield.

**Scheme 3:**
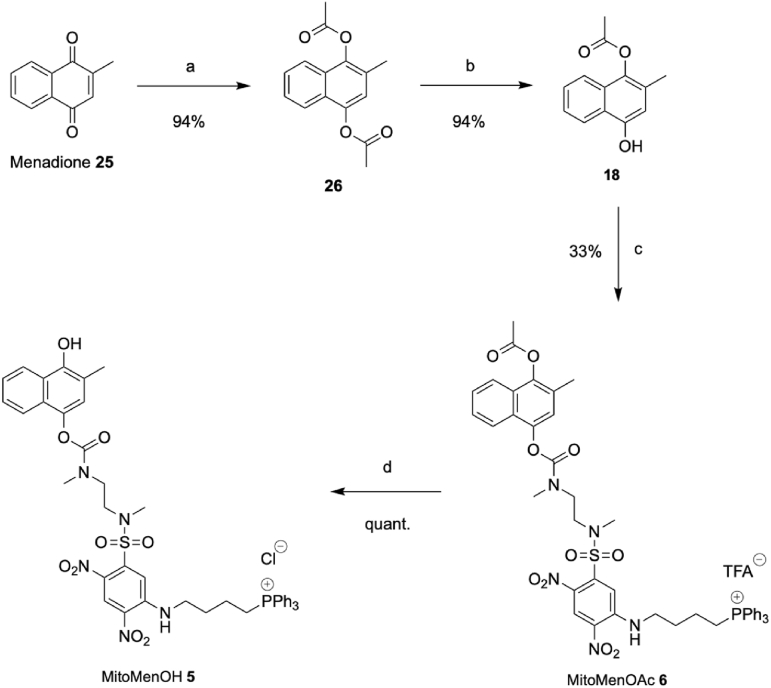
Synthesis of MitoMenOH **5** and MitoMenOAc **6**. a: Ac_2_O, DMAP, Pd/C, EtOAc, H_2_, RT, 24 h. b: ^i^Pr_2_NH, MeOH, H_2_O, RT, 24 h. c: (i) (Cl_3_CO)_2_CO, EtN^i^Pr_2_, RT, 1 h (ii) **24**, EtN^i^Pr_2_, DCM, RT, 24 h. d: HCl (6 M), MeOH, RT, 24 h.

Synthesis of prodrugs of menadiol **17** began from commercially available menadione **25** (Scheme 3). As menadiol **17** spontaneously oxidises to menadione **25**, it was coupled to the prodrug scaffold as the monoacetylated analogue **18**, and then finally deacetylated. Menadione **25** was reduced and acetylated *in situ* to form the diacetate **26** in excellent yield as described previously [36]. Diacetate **26** was hydrolysed under basic conditions to obtain the monoacetate **18** in excellent yield. The monoacetate **18** was coupled to the ammonium scaffold **24** in the presence of base to afford the double prodrug MitoMenOAc **6**, albeit in poor yield. Some of MitoMenOAc **6** was hydrolysed under acidic conditions to obtain the single prodrug, MitoMenOH **5**, in a quantitative yield.

**Scheme 4:**
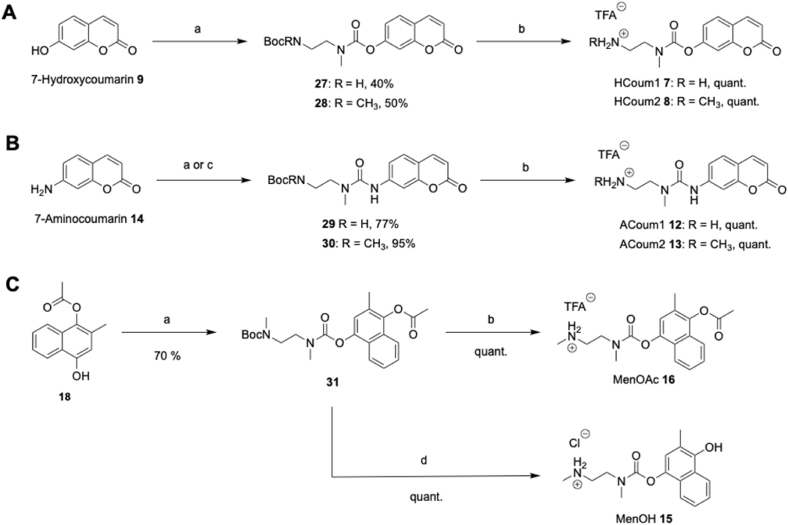
Synthesis of intermediates. (A) Synthesis of HCoum1 **7** and HCoum2 **8**. (B) Synthesis of ACoum1 **12** and ACoum2 **13**. (C) Synthesis of MenOH **15** and MenOAc **16**. a: (i) (Cl_3_CO)_2_CO, EtN^i^Pr_2_, DCM, RT, 1 h, (ii) H_2_N(CH_2_)_2_N(Me)Boc or MeNH(CH_2_)_2_N(Me)Boc, EtN^i^Pr_2_, DCM, RT, 16 h. b: TFA, DCM, RT, 1h. c: (i) COCl_2_/Toluene, NEt_3_, DCM, 0 °C, 1 h, (ii) H_2_N(CH_2_)_2_N(Me)Boc, EtN^i^Pr_2_, DCM, RT, 16 h. d: HCl (6 M), MeOH, RT, 24 h.

The TFA salts, **7•TFA** and **8•TFA**, of payload-linker conjugates HCoum1 **7** and HCoum2 **8** were prepared from commercially-available 7-hydroxycoumarin **9** (Scheme 4A). 7-Hydroxycoumarin **9** was reacted with triphosgene, followed by addition of *N*-Boc-*N*-methylethylenediamine or *N*-Boc-*N*,*N′*-dimethylethylenediamine, which afforded the Boc-protected compounds **27** and **28**, respectively (Fig. 5A). Deprotection with trifluoroacetic acid afforded HCoum1 **7** and HCoum2 **8** as the trifluoroacetic acid salts in quantitative yield. To synthesise ACoum1 **12** and ACoum2 **13**, 7-aminocoumarin **14** was first prepared from 7-hydroxycoumarin **9** in a two-step synthesis (Scheme S1) [36]. 7-Aminocoumarin **14** was reacted with phosgene or triphosgene, followed by *N*-Boc-*N*-methylethylenediamine or *N*-Boc-*N*,*N′*-dimethylethylenediamine, which afforded the Boc-protected compounds **29** and **30**, respectively (Scheme 4B). Deprotection as before gave ACoum1 **12** and ACoum2 **13** as the trifluoroacetic acid salts in quantitative yield. The ethylenediamine carbamate derivatives MenOH **15** and MenOAc **16** were prepared from monoacetate **18** (Scheme 4C). The monoacetate **18** was reacted with triphosgene and *N*-Boc-*N*,*N′*-dimethylethylenediamine which afforded the Boc-protected compound **31** in good yield. Treatment of compound **31** with hydrochloric acid obtained MenOH **15** as the hydrochloric acid salt in quantitative yield. Treatment of compound **31** with trifluoroacetic acid obtained MenOAc **16** as the trifluoroacetic acid salt in quantitative yield. The intermediates MenOH **15** and MenOAc **16** were prepared from monoacetate **18**. The monoacetate **18** was reacted with triphosgene and *N*-Boc-*N*,*N′*-dimethylethylenediamine, which afforded the Boc-protected compound **31** in good yield (Fig. 5C). Treatment of compound **31** with hydrochloric acid obtained MenOH **15** as the ammonium hydrochloric acid salt in quantitative yield. Treatment of compound **31** with trifluoroacetic acid obtained MenOAc **16** as the ammonium trifluoroacetic acid salt in quantitative yield.

### *2.2* *In vitro* reactivity of coumarin prodrugs and analogues analysed by fluorescence

Next, we investigated how quickly a phenolic payload would be released from a TPP-dinitroaryl-linker-payload conjugate under a biologically plausible concentration of 10 mM mGSH (Fig. 1B). MitoHCoum1 **3** and MitoHCoum2 **4** are full constructs designed to release the fluorescent reporter, 7-hydroxycoumarin **9**, which would be used to investigate the overall rate of release (Scheme 1A). The rates of each step in the release mechanism are also important to the delivery since the first GSH-activated step separates an inactive linker-payload conjugate from the TPP targeting group, and only then is the active payload released by cyclative self-immolation of the linker. A very slow second step might allow the linker-payload to redistribute from the mitochondria before releasing the payload. Two sets of compounds were developed to investigate each step independently. MitoACoum1 **9** and MitoACoum2 **10** resemble the full constructs **3** and **4**, but only undergo the first step (Scheme 1B), while HCoum1 **7** and HCoum2 **8** are linker-payload constructs that only undergo the second step (Scheme 1A). We began by investigating these simple one-step reactions.

**Fig. 2:**
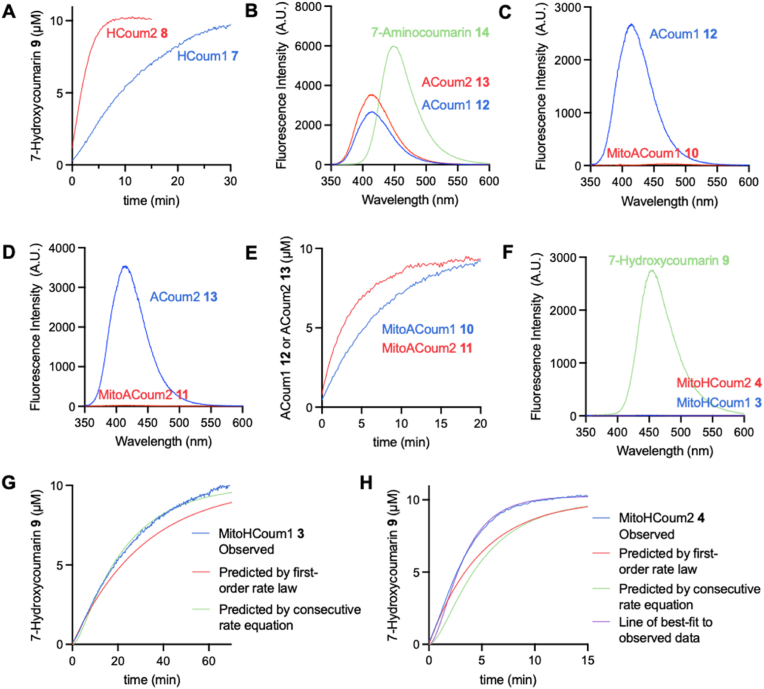
Fluorescence spectra and kinetics of coumarin prodrugs (10 μM) in HEPES pH 8.0 buffer at 30 °C. (A) Reaction of HCoum1 **7** and HCoum2 **8** monitored by measuring formation of 7-hydroxycoumarin **9** (λ_ex_ = 342 nm, λ_em_ = 454 nm). (B) Fluorescence spectra of ACoum1 **12** (λ_ex_ = 330 nm, λ_em_ = 414 nm), ACoum2 **13** (λ_ex_ = 330 nm, λ_em_ = 414 nm) and 7-aminocoumarin **14** (λ_ex_ = 342 nm, λ_em_ = 448 nm). (C) Fluorescence spectra of MitoACoum1 **10** and product ACoum1 **12** (both λ_ex_ = 330 nm, λ_em_ = 414 nm). (D) Fluorescence spectra of MitoACoum2 **11** and product ACoum2 **13** (both λ_ex_ = 330 nm, λ_em_ = 414 nm). (E) Reactions of MitoACoum1 **10** and MitoACoum2 11 with GSH (2 mM) monitored by measuring formation of ACoum1 **12** or ACoum2 13, respectively. (F) Fluorescence spectra of MitoHCoum1 **3**, MitoHCoum2 **4** and product 7-hydroxycoumarin **9** (all λ_ex_ = 342 nm, λ_em_ = 454 nm). (G) Blue line shows the observed reaction of MitoHCoum1 **3** and GSH (10 mM) monitored by measuring formation of 7-hydroxycoumarin **9**. Red line shows the predicted reaction when treating it as first-order, calculated using the integrated rate law for a first-order reaction and *k* = 0.532 × 10^−3^ s^1^ determined for the observed reaction of MitoHCoum1 **3**. Green line shows the predicted reaction calculated using the consecutive reaction equation, *k*_*1*_ = 6.40 × 10^−3^ s^−1^ from the reaction of MitoACoum1 **10** adjusted for 10 mM GSH and *k*_*2*_ = 0.770 × 10^−3^ s^−1^ for the reaction of HCoum1 **7**. (H) Blue line shows the observed reaction of MitoHCoum2 **4** and GSH (10 mM) monitored by measuring formation of 7-hydroxycoumarin **9**. Red line shows the predicted reaction when treating it as first-order, calculated using the integrated rate law for a first-order reaction and *k* = 3.39 × 10^−3^ s^1^ determined for the observed reaction of MitoHCoum2 **4**. Green line shows the predicted reaction calculated using the consecutive reaction equation, *k*_*1*_ = 16.1 × 10^−3^ s^−1^ from the reaction of MitoACoum2 **11** adjusted for 10 mM GSH and *k*_*2*_ = 3.39 × 10^−3^ s^−1^ for the reaction of HCoum2 **8**. Magenta line shows the non-linear line of best-fit to the observed reaction using the consecutive reaction equation, *k*_*1*_ = 16.1 × 10^−3^ s^−1^ from the reaction of MitoACoum2 **11** adjusted for 10 mM GSH and *k*_*2*_ = 6.85 × 10^−3^ s^−1^ calculated by the least-squares method. Kinetics data are means ± SD, N = 3. Fluorescence spectra data are single measurements. (For interpretation of the references to colour in this figure legend, the reader is referred to the Web version of this article.)

To measure the kinetics of cyclisation of the self-immolative ethylenediamine carbamate linker, HCoum1 **7•TFA** and HCoum2 **8•TFA** were incubated at pH 8.0, and release of 7-hydroxycoumarin **9** was monitored by fluorescence (Scheme 1A). The fluorescence of the phenoxide form of 7-hydroxycoumarin **9** was used because this is at a longer wavelength of 454 nm. Initial rates (Fig. S2A and S2B) were converted to concentration per second using the appropriate calibration curve for the 7-hydroxycoumarin **9** (Fig. S1A). HCoum1 **7** and HCoum2 **8** released 7-hydroxycoumarin **9** rapidly (Fig. 2A, Table 1, entries 1 and 2). Apparent first-order rate constants were calculated assuming that the equilibrium between the protonated and unprotonated forms of HCoum1 **7** and HCoum2 **8** was rapid relative to cyclisation. The apparent first-order rate constant for the cyclisation of *N,N-*dimethyl analogue HCoum2 **8** (entry 2) is ∼5-fold greater than that of mono-*N-*methyl analogue HCoum1 **7** (entry 1). This is in broad agreement with Saari et al. [27], who found that the release of 4-hydroxyanisole from carbamate analogues containing an ethylenediamine spacer was ∼8-fold faster when both nitrogen atoms were methylated compared to only a single methyl group on the carbamate nitrogen.

**Table 1:** Rate constants for the reactions of the coumarin analogues at pH 8.0 with or without GSH. Conditions: (a) 10 μM compound, HEPES (0.1 M) pH 8.0 buffer, 30 °C. (b) 10 μM compound, 2 mM GSH, HEPES (0.1 M) pH 8.0 buffer, 30 °C. (c) 10 μM compound, 10 mM GSH, pH 8.0 HEPES (0.1 M) buffer, 30 °C. Data are means ± SD, N = 3.

Entry	Compound	R^1^	R^2^	First order rate constant^(a)^ (x 10^−3^ s^−1^)	Observed pseudo-first order rate constant^(b)^ (x 10^−3^ s^−1^) [GSH] = 2 mM	Calculated second order rate constant (M^−1^ s^−1^)	Calculated pseudo-first order rate constant^(c)^ (x 10^−3^ s^−1^) [GSH] = 10 mM
1	HCoum1 **7**	H	O	0.770 ± 0.085			
2	HCoum2 **8**	CH_3_	O	3.76 ± 0.09			
3	MitoACoum1 **10**	H	NH		1.28 ± 0.30	0.640 ± 0.15	6.40 ± 1.53
4	MitoACoum2 **11**	CH_3_	NH		3.23 ± 0.19	1.61 ± 0.09	16.1 ± 0.9

To measure the kinetics of the GSH-activated release of the linker-payload conjugate, we used the compounds MitoACoum1 **10** and MitoCoum2 **11** that resemble the full constructs but could only perform the first step. MitoACoum1 **10** and MitoACoum2 **11** were treated with GSH at pH 8.0 and their reaction monitored by the fluorescence of the released ethylenediamine urea products, ACoum1 **12** and ACoum2 **13**, respectively (Scheme 1B). ACoum1 **12** and ACoum2 **13** fluoresced at 414 nm, were stable at pH 8.0, and did not release 7-aminocoumarin **14**, which fluoresces at 448 nm (Fig. 2B). In contrast, the compounds MitoACoum1 **10** and MitoACoum2 **11** were not fluorescent at pH 8.0 (Fig. 2C and D). The reaction of MitoACoum1 **10** or MitoACoum2 **11** with 10 mM GSH, to mimic the mitochondrial matrix, was too fast to measure, so 2 mM GSH was used instead. Initial rates for reaction of MitoACoum1 **10** and MitoACoum2 **11** (Fig. S2C and S2D) were converted to concentration per second using the calibration curves for ACoum1 **11** and ACoum2 **12**, respectively (Fig. S1B and S1C). An increase in fluorescence at 414 nm indicated that MitoACoum1 **10** and MitoACoum2 **11** reacted with GSH to produce ACoum1 **12** and ACoum2 **13**, respectively (Fig. 2E–Table 1, entries 3 and 4). MitoACoum2 **11** (entry 4) reacted with GSH ∼2.5-fold faster than MitoACoum1 **10** (entry 3). This difference in reactivity may be plausibly attributed to deprotonation of about 60% of the secondary sulfonamide MitoACoum1 **10**, so that only 40% of the compound is reactive towards S_N_Ar at pH 8.0. The addition step to generate the Meisenheimer intermediate is rate-determining in the step-wise S_N_Ar reaction [25] and the sulfonamidate anion is much less inductively electron-withdrawing. A pKa of 7.8 for MitoACoum1 **10** is reasonable given that the similarly electron-poor pentafluorbenzenesulfonamide has a pKa of 8.2 [37,38]. In contrast, since MitoACoum2 **11** is a tertiary sulfonamide, it cannot form an intramolecular hydrogen bond.

In preparation for interpreting the kinetics for the full constructs **3** and **4**, we used the data from the one-step reactions to predict whether either step would be rate-determining when the concentration of GSH was 10 mM. The second order rate constants for MitoACoum1 **10** and MitoACoum2 **11** (Table 1, entries 3 and 4) were calculated from the pseudo-first order rate constant for reaction with 2 mM GSH, and these were used to calculate a pseudo-first-order rate constant for the reaction at 10 mM GSH. At this concentration of GSH, the pseudo-first-order rate constant of MitoACoum1 **10** is ∼8.3-fold greater than the first-order rate constant of HCoum1 **7**. Similarly, the pseudo-first-order rate constant of MitoACoum2 **11** is ∼4.3-fold greater than the first-order rate constant of HCoum2 **8**. Therefore, in both cases, we expected that GSH-activated release of the linker-payloads, **7** and **8**, from the full constructs, **3** and **4**, would be significantly faster than cyclisation of the linker-payload intermediates, **7** and **8**, meaning the second step would likely contribute most to determining the overall rate of reaction.

To measure the overall reactions of MitoHCoum1 **3** and MitoHCoum2 **4**, the compounds were reacted with GSH at pH 8.0 and monitored by fluorescence of the final product 7-hydroxycoumarin **9** (Scheme 1A). As before, initial rates (Fig. S2E and S2F) were converted to concentration per second using the appropriate calibration curve for the 7-hydroxycoumarin **9** (Fig. S1A). MitoHCoum1 **3** and MitoHCoum2 **4** were not fluorescent at pH 8.0 (Fig. 2F). Both compounds were treated with 10 mM GSH to mimic physiological conditions in the mitochondrial matrix, and to ensure that the first step, GSH-activated release of the linker-payload construct, would be pseudo-first-order. MitoHCoum1 **3** and MitoHCoum2 **4** reacted with 10 mM GSH at pH 8.0 to release 7-hydroxycoumarin **9** via the two-step reaction (Fig. 2G and H, blue lines). Initially, reactions of MitoHCoum1 **3** and MitoHCoum2 **4** were interpreted using a simple consecutive reaction model. In this model a reactant **A** converts to an intermediate **B** in a first-order reaction, which in turn converts to product **C** in another first-order reaction. This gives the rate equation:

(1) $$C_{c}=C_{0}\left[1-\frac{\left({k_{2}e}^{-k_{1}t}-{k_{1}e}^{-k_{2}t}\right)}{\left(k_{2}-k_{1}\right)}\right]$$

**Scheme 5:**
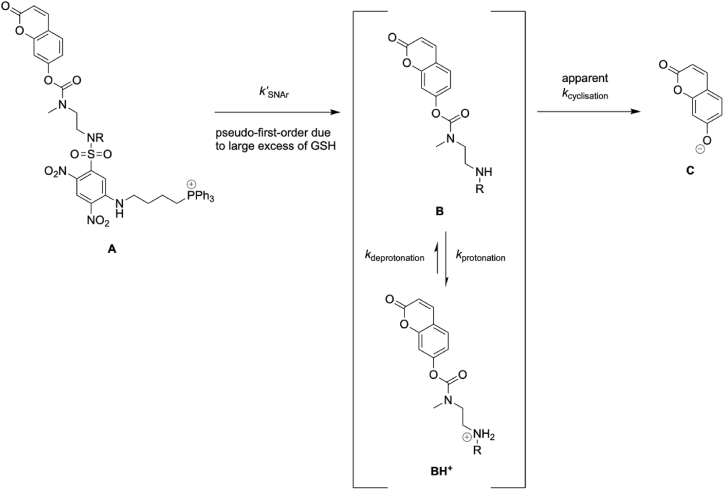
Consecutive reaction model applied to MitoHCoum1 **3** and MitoHCoum2 **4**.

Here, the reactant **A** is MitoHCoum1 **3** or MitoHCoum2 **4**, and the intermediate **B** is the amine form of the linker-payload construct, HCoum1 **7** or HCoum2 **8** (Scheme 5). Two key assumptions were made. We assumed that the pseudo-first-order rate constant for MitoACoum1 **10** and MitoACoum2 **11** were the same as the pseudo-first-order rate constants for MitoHCoum1 **3** or MitoHCoum2 **4**, respectively, and so could be used as the rate constants for **A** converting to **B**. We also assumed that establishing the equilibrium between **BH^+^** and **B** would be faster than the cyclisation, so that the apparent rate of **B** converting to **C** would not be affected by whether **B** was formed from **A** or from **BH^+^**. This would mean that the apparent first order rate constants for HCoum1 **7•TFA** or HCoum2 **8•TFA** (corresponding to **BH^+^**) could be used for the cyclisation step in the consecutive reaction model. Gratifyingly, there is a near-perfect match between the observed reaction of MitoHCoum1 **3** (Fig. 2G, blue line) and the consecutive reaction model when the pseudo-first-order rate constant for MitoACoum1 **10** is used as *k’*_SNAr_ and the apparent rate constant for cyclisation HCoum1 **7•TFA** is used for *k*_cyclisation_ (Fig. 2G, green line). Furthermore, the consecutive reaction model is better than using an apparent first order rate constant calculated by applying first-order kinetics to the initial rate of reaction of MitoHCoum1 **3** (Fig. 2G, red line, *k*_*apparent*_ = 0.532 ± 0.019 × 10^−3^ s^−1^). MitoHCoum2 **4** releases the 7-hydroxcoumarin **9** payload extremely quickly, with reaction complete in under 10 min. It is so fast that when MitoACoum2 **4** is used as *k’*_SNAr_ and the apparent rate constant for cyclisation HCoum2 **8•TFA** is used for *k*_cyclisation_, the simple consecutive reaction model (Fig. 2H, green line) underestimates the observed rate of fluorophore release (Fig. 2H, blue line). Given that the apparent *k*_cyclisation_ of HCoum2 **8•TFA** is ∼5-fold greater than that of HCoum1 **7•TFA**, we reasoned that in the reaction of MitoHCoum2 **4**, where non-protonated HCoum2 **8** is generated in the first step, the cyclisation competes to some extent with protonation at pH 8.0. The consequence of this would be that the second assumption, in which the rate of establishing an equilibrium is fast relative to cyclisation, breaks down, and the apparent *k*_cyclisation_ of HCoum2 **8•TFA** is too low to model the kinetics effectively. Following this reasoning, we used MitoACoum2 **11** as *k’*_SNAr_ and modelled a curved line of best-fit to the observed data of MitoHCoum2 **4** by the least-squares method (Fig. 2H, magenta line), which returned *k*_cyclisation_ = 6.85 × 10^−3^ s^−1^. This refined application of the consecutive reaction model was far better than using an apparent first-order rate constant calculated by applying first-order kinetics to the initial rate of reaction of MitoHCoum2 **4** (Fig. 2H, red line, *k*_*apparent*_ = 3.39 ± 0.01 × 10^−3^ s^−1^).

Overall, the kinetics analysis of MitoHCoum1 **3** and MitoHCoum2 **4** demonstrate that these prodrug scaffolds rapidly release a phenolic payload under conditions that mimic those in the mitochondrial matrix (10 mM GSH, pH 8.0). MitoHCoum2 **4** reacts particularly quickly with almost complete release of 7-hydroxycoumarin **9** in 10 min. The reactions of both compounds could be well-modelled as consecutive reactions. For MitoCoum1 **3**, using the determined rate constants for model compounds **10** and **7** in the consecutive reaction model gave an excellent fit to observed kinetics with *k’*_*SNAr*_ = 6.40 × 10^−3^ s^−1^ and *k*_*cyclisation*_ = 7.70 × 10^−4^ s^−1^. MitoCoum2 **4** reacted faster than expected from the kinetics of model compounds **11** and **8**. However, an excellent fit was obtained using the determined rate constant for compound **11** as *k’*_*SNAr*_ = 1.61 × 10^−2^ s^−1^, and a *k*_*cyclisation*_ = 6.0 × 10^−3^ s^−1^. Thus, for both MitoHCoum1 **3** and MitoHCoum2 **4**, the cyclisation step is slower than the S_N_Ar and so contributes most to determining the rate of payload release. However, the rapid release of the payload from MitoCoum2 **4** was promising, and this compound and the menadione derivatives with the same linker, MitoMenOH **5** and MitoMenOAc **6**, were taken for evaluation in biological systems.

### *2.3* *In vitro* reactivity of prodrugs analysed by RP-HPLC

**Fig. 3:**
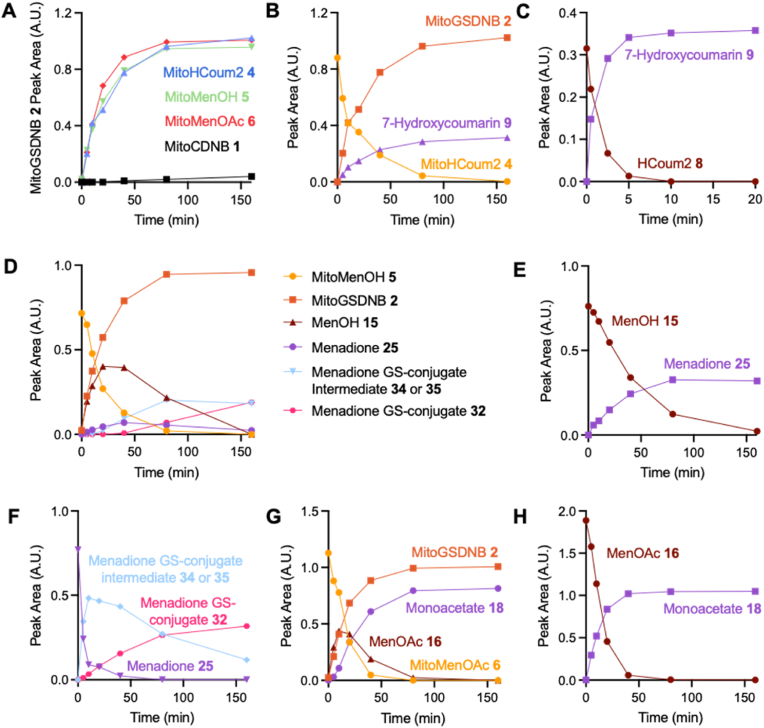
Reactions of prodrugs and intermediates (10 μM) with or without GSH (100 μM) at pH 8.0 and 37 °C analysed by RP-HPLC. (A) Relative rates of formation of MitoGSDNB **2** from MitoCDNB **1**, MitoHCoum2 **4**, MitoMenOH **5** and MitoMenOAc **6**. Absorbance was measured at 220 nm. (B) Reaction of MitoHCoum2 **4** with GSH at pH 8.0. Absorbance was measured at 220 nm. The intermediate HCoum2 **8** was not detected. (C) Reaction of HCoum2 **8** at pH 8.0. Absorbance was measured at 220 nm. D) Reaction of MitoMenOH **5** with GSH at pH 8.0. Absorbance was measured at 220 nm for MitoMenOH **5**, MitoGSDNB **2** and MenOH **15**, and at 254 nm for menadione **25**, the menadione GS-conjugate intermediate and menadione GS-conjugate. (E) Reaction of MenOH **15** at pH 8.0. Absorbance was measured at 220 nm for MenOH **15** and at 254 nm for menadione **25**. (F) Reaction of menadione **25** with GSH at pH 8.0. Absorbance was measured at 254 nm. (G) Reaction of MitoMenOAc **6** with GSH at pH 8.0. Absorbance was measured at 220 nm. (H) Reaction of MenOAc **16** at pH 8.0. Absorbance was measured at 220 nm. Data are single measurements.

**Scheme 6:**
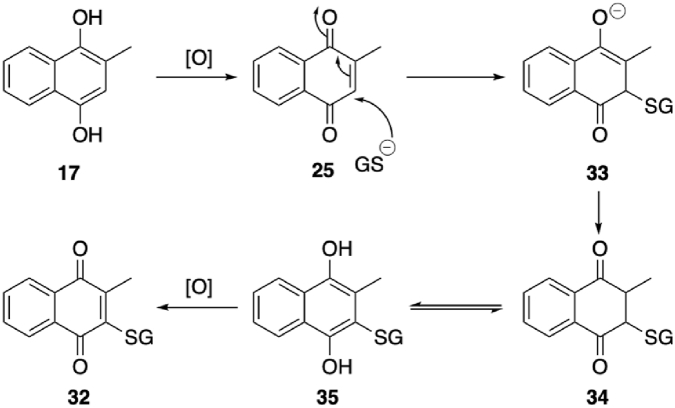
Proposed mechanism of reaction of menadione **25** with GSH to form GS-menadione conjugate **32**.

The reactions of MitoHCoum2 **4**, MitoMenOH **5** and MitoMenOAc **6** with GSH at pH 8.0 to form MitoGSDNB **2** were compared to that of MitoCDNB **1** by RP-HPLC. The compounds were incubated with only a 10-fold excess of GSH to facilitate assessment of the formation of MitoGSDNB **2**, the intermediates and the products. The formation of MitoGSDNB **2** by reaction of MitoHCoum2 **4**, MitoMenOH **5** and MitoMenOAc **6** was significantly faster than that of MitoCDNB **1** (Fig. 3A) in the absence of GSTs. This is due to the higher electron-withdrawal of the sulfonamide moiety compared to the chloride substituent. The reaction of MitoHCoum2 **4** with GSH produced MitoGSDNB **2** and 7-hydroxycoumarin **9** (Fig. 3B), but the putative intermediate HCoum2 **8** was not detected, presumably due to rapid conversion of HCoum2 **8** to 7-hydroxycoumarin **9**, which was confirmed by the reaction of HCoum2 **8** at pH 8.0 (Fig. 3C). Reaction of MitoMenOH **5** with GSH produced MitoGSDNB **2** and the ethylenediamine carbamate intermediate MenOH **15**, the latter of which fragmented to release menadiol **17**, which spontaneously oxidised to form menadione **25** (Fig. 3D). The menadione **25** signal was weak due to reaction with GSH to form the GS-menadione conjugate **32**, confirmed by LCMS/MS (Scheme 6, Fig. S3). This would be expected to form via spontaneous oxidation of the released menadiol **16** to menadione **25**, followed by Michael addition of GSH to give enolate **33**. Rapid protonation would then give the 2,3-dihydronaphthoquinone **34**, which would be in equilibrium with its tautomeric GS-menadiol conjugate **35** [39]. The latter would then oxidize to the GS-menadione conjugate **32.** Indeed, RP-HPLC suggested that this reaction proceeded via a relatively long-lived intermediate, which is likely either diketone **34** or the GS-menadiol conjugate **35**. Spontaneous oxidation of MenOH **15** to menadione **25** at pH 8.0 in the absence of GSH was demonstrated independently (Fig. 3E). Treatment of menadione **25** with GSH at pH 8.0 confirmed GS-conjugation (Fig. 3F) in line with literature [[40], [41], [42], [43]]. The GS-conjugate itself can also undergo redox cycling to generate O_2_^•-^ [42].

Reaction of MitoMenOAc **6** with GSH produced MitoGSDNB **2** and ethylenediamine carbamate intermediate MenOAc **16**, the latter of which fragmented to release the monoacetate **18** (Fig. 3G). Conversion of MenOAc **16** to monoacetate **18** at pH 8.0 was also demonstrated over this timescale (Fig. 3H). The monoacetate **18** was stable at pH 8.0, suggesting that rapid hydrolysis to form menadiol **17** requires esterase activity. The model compound of the ethylenediamine carbamate intermediate, HCoum2 **8**, reacted significantly faster than MenOAc **16**, which in turn reacted faster than MenOH **15**, with half-lives of ∼2 min, 15 min and 35 min, respectively. The reactivity trend can be explained by the fact that HCoum2 **8** is more electron-withdrawing by inductive effects than MenOAc **16**, which in turn is more electron-withdrawing than MenOH **15**. Collectively, these results confirm that the dual-action prodrug scaffold sequesters GSH, via formation of MitoGSDNB **2**, and releases a phenolic payload. These compounds are highly reactive with GSH owing to the dinitrobenzene ring and the sulfonamide moiety, which means that they do not require GST catalysis unlike MitoCDNB **1** and previous sulfonamide prodrugs of doxorubicin [22] and metformin [23].

### 2.4 Uptake and reaction of prodrugs in isolated mitochondria

**Fig. 4:**
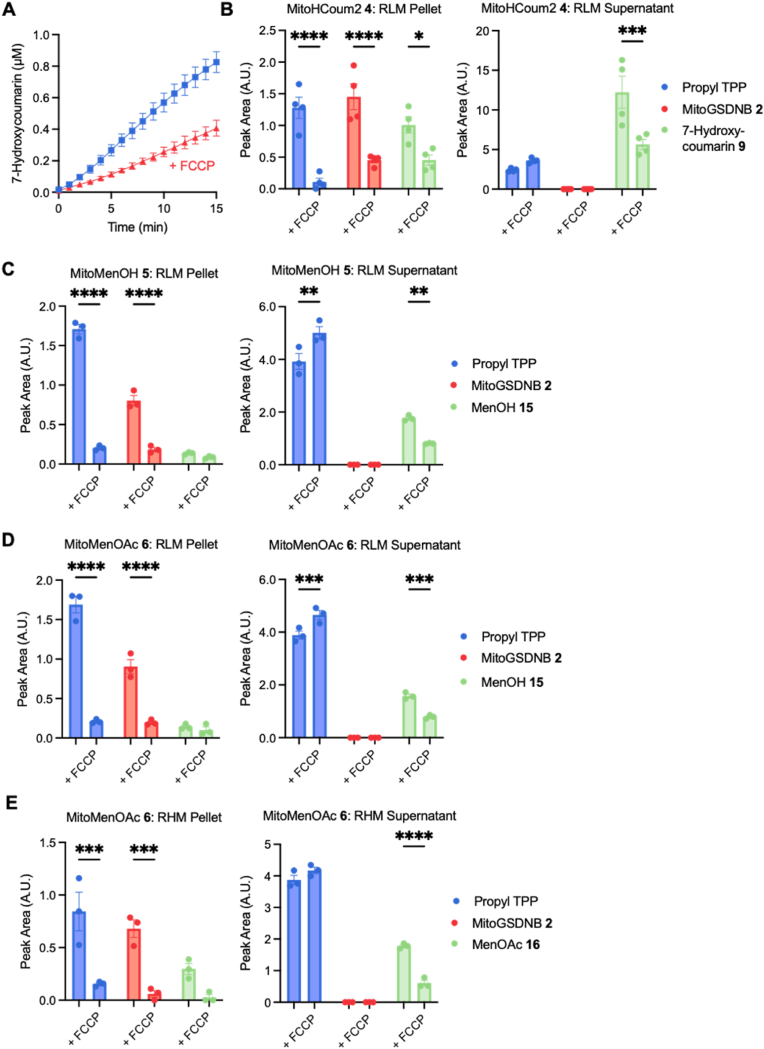
Uptake and reactions of prodrugs with isolated rat liver mitochondria (RLM) or rat heart mitochondria (RHM). (A) MitoHCoum2 **4** (5 μM) was incubated with RLM (1 mg protein/mL) supplemented with succinate (10 mM) and rotenone (10 μM) ± FCCP (1 μM) and the fluorescence of 7-hydroxycoumarin **9** was measured on a plate reader. (B) MitoHCoum2 **4** (5 μM) was incubated with RLM (1 mg protein/mL) supplemented with glutamate (10 mM) and malate (10 mM) ± FCCP (1 μM) and the pellet and supernatant were analysed by RP-HPLC. (C) MitoMenOH **5** (5 μM) was incubated with RLM (1 mg protein/mL) supplemented with glutamate (10 mM) and malate (10 mM) ± FCCP (1 μM) and the pellet and supernatant were analysed by RP-HPLC. D) MitoMenOAc **6** (5 μM) was incubated with RLM (1 mg protein/mL) supplemented with glutamate (10 mM) and malate (10 mM) ± FCCP (1 μM) and the pellet and supernatant were analysed by RP-HPLC. (E) MitoMenOAc **6** (5 μM) was incubated with RHM (1 mg protein/mL) supplemented with glutamate (10 mM) and malate (10 mM) ± FCCP (1 μM) and the pellet and supernatant were analysed by RP-HPLC. Data are means ± SEM, N ≥ 3. p values were calculated using a two-way ANOVA test. ∗p < 0.05, ∗∗p < 0.01, ∗∗∗p < 0.001, ∗∗∗∗p < 0.0001.

MitoHCoum2 **4**, MitoMenOH **5** and MitoMenOAc **6** should accumulate within energised mitochondria owing to their TPP group. Inside the mitochondria they should react with the high local mGSH concentration (1-10 mM [1,5]) depleting mGSH to produce MitoGSDNB **2** and a short-lived intermediate, which then fragments to release the phenolic payload. To assess uptake and reactivity, MitoHCoum2 **4** was incubated with rat liver mitochondria and the formation of 7-hydroxycoumarin **9** was measured by fluorescence (Fig. 4A and S4 for calibration curve). The reaction showed a steady increase in 7-hydroxycoumarin **9** over time that was slowed by dissipation of the membrane potential with the uncoupler 4-(trifluoromethoxy)phenylhydrazone (FCCP). To confirm uptake and reactivity within isolated mitochondria, MitoHCoum2 **4**, MitoMenOH **5** and MitoMenOAc **6** were incubated with liver or heart mitochondria, the mitochondria were pelleted, and both the supernatants and pellets were analysed by RP-HPLC. Mitochondrial uptake of unreactive propyl TPP was used as a control to confirm membrane potential generation. The bulk of MitoHCoum2 **4**, MitoMenOH **5** and MitoMenOAc **6** was associated with the mitochondrial pellet with or without FCCP (Figs. S5A, S5B, S5C and S5D), while propyl TPP uptake was significantly decreased by FCCP. This is consistent with the highly lipophilic prodrugs strongly associating with mitochondrial membranes irrespective of membrane potential.

**Fig. 5:**
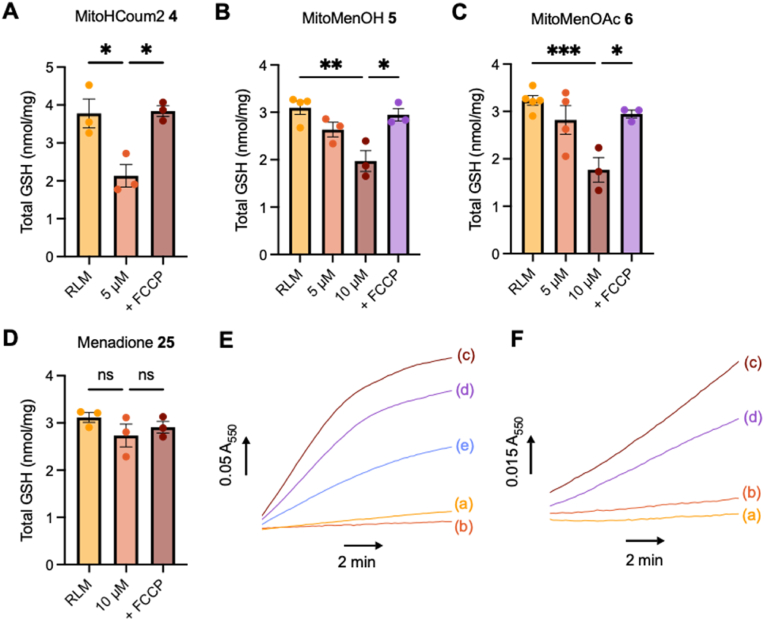
Total GSH content of RLM incubated with the prodrugs and measurement of O_2_^•-^ production by BHM supplemented with menadione **25**. (A) MitoHCoum2 **4** (5 μM) was incubated with RLM (1 mg protein/mL) supplemented with glutamate (10 mM) and malate (10 mM) ± FCCP (1 μM) and the matrix levels of GSH were measured. (B) MitoMenOH **5** (5 or 10 μM) was incubated with RLM (1 mg protein/mL) supplemented with glutamate (10 mM) and malate (10 mM) ± FCCP (1 μM) and the matrix levels of GSH were measured. (C) MitoMenOAc **6** (5 or 10 μM) was incubated with RLM (1 mg protein/mL) supplemented with glutamate (10 mM) and malate (10 mM) ± FCCP (1 μM) and the matrix levels of GSH were measured. (D) Menadione **25** (10 μM) was incubated with RLM (1 mg protein/mL) supplemented with glutamate (10 mM) and malate (10 mM) ± FCCP (1 μM) and the matrix levels of GSH were measured. (E) Reduction of cyt c_acet_ by BHM (100 μg protein/mL) supplemented with myxothiazol (0.4 μM), KCN (2 mM) and either (a) NADH (1 mM), (b) menadione **25** (50 μM), (c) NADH (1 mM) and menadione **25** (50 μM), (d) NADH (1 mM), menadione **25** (50 μM) and rotenone (20 μM), or (e) NADH (1 mM), menadione **25** (50 μM) and SOD (100 U/mL). (F) Reduction of cyt c_acet_ by BHM (100 μg protein/mL) supplemented with rotenone (20 μM), myxothiazol (0.4 μM), KCN (2 mM) and either (a) succinate (1 mM), (b) menadione **25** (50 μM), (c) succinate (1 mM) and menadione **25** (50 μM), or (d) succinate (1 mM), menadione **25** (50 μM) and SOD (100 U/mL). Total GSH data (A), (B), (C) and (D) are means ± SEM, N ≥ 3. p values were calculated using a one-way ANOVA test. ∗p < 0.05, ∗∗p < 0.01, ∗∗∗p < 0.001. O_2_^•-^ production data (E) and (F) are means of N = 3 independent experiments.

For MitoHCoum2 **4**, MitoGSDNB **2** and 7-hydroxycoumarin **9** were detected within mitochondria (Fig. 4B). FCCP decreased the two products, supporting the membrane potential-dependent uptake seen earlier (Fig. 4A). The intermediate HCoum2 **8** was not observed due to its short half-life (Fig. 3C). MitoGSDNB **2** was only detected in the pellet because it is formed within mitochondria and is membrane-impermeant. In contrast, ∼10-fold more 7-hydroxycoumarin **9** was detected in the supernatant than in the pellet, indicating that it rapidly diffuses out of the mitochondria. While it is also possible that the intermediate HCoum2 **8** could diffuse out of the mitochondria and fragment in the cytosol, this is less likely because it would be predominantly in its charged protonated form at pH 8 and its half-life is very short.

For MitoMenOH **5**, MitoGSDNB **2** and the intermediate MenOH **15** were detected, and their amounts were decreased by FCCP (Fig. 4C). Neither menadione **25** nor the menadione GS-conjugate were detected, consistent with the long half-life of MenOH **15** at pH 8.0 (Fig. 3E). Approximately 10-fold more MenOH **15** was detected in the supernatant compared to the pellet, consistent with its diffusion out of the mitochondria.

When using liver mitochondria, MitoMenOAc **6** was rapidly hydrolysed to MitoMenOH **5** by cytosolic esterases, associated with the mitochondrial preparation, prior to accumulation (Fig. S5C). As a result, the reaction emulated that of MitoMenOH **5**; only MitoGSDNB **2** and MenOH **15** (but not MenOAc **16**) were detected (Fig. 4D). When MitoMenOAc **6** was incubated with heart mitochondria, which contain far fewer contaminating esterases, no hydrolysis was observed, but MitoMenOAc **6** still associated with the outside of the mitochondrial pellet (Fig. S5D), so only the intermediate and products are presented. MitoGSDNB **2** and MenOAc **16** were detected in the pellet and FCCP decreased their production, indicating membrane potential-dependent uptake of MitoMenOAc **6** (Fig. 4E). A higher proportion of the intermediate was present in the supernatant than in the pellet, reflecting diffusion out of the mitochondria. Neither the monoacetate **18** nor menadione **25** were detected likely because the half-life of MenOAc **16** at pH 8.0 is ∼15 min (Fig. 3H).

Next, we assessed mGSH depletion by MitoHCoum2 **4**, MitoMenOH **5** and MitoMenOAc **6**. At 5 μM MitoHCoum2 **3** depleted over 40 % of mGSH after 15 min (Fig. 5A), while depletion by MitoMenOH **5** (Fig. 5B) and MitoMenOAc **6** (Fig. 5C) was minimal. This is consistent with the relative levels of MitoGSDNB **2** measured in the mitochondrial pellets (Fig. 4B, C and 4D). At 10 μM MitoMenOH **5** depleted mGSH by over 30 % (Fig. 5B) and MitoMenOAc **6** depleted mGSH by over 40 % (Fig. 5C). In all cases, mGSH depletion was attenuated by FCCP. 10 μM menadione **25** had a negligible effect on mGSH levels (Fig. 5D). Therefore, the prodrugs accumulate within isolated mitochondria where they deplete mGSH via formation of MitoGSDNB **2**.

### 2.5 Menadione generates superoxide in incubation with mitochondrial membranes

To evaluate menadione's potential to redox cycle it was incubated with bovine heart mitochondrial membranes (BHM) and O_2_^•-^ production was monitored via reduction of acetylated cytochrome *c* (cyt *c*_acet_). Menadione **25** undergoes reduction to menadiol **17** at complex I [32], which subsequently reacts with O_2_ to produce O_2_^•-^ and regenerate menadione **25**. BHM alone produced negligible O_2_^•-^ when supplemented with NADH (Fig. 5E, trace a) or menadione **25** (Fig. 5E, trace b). When BHM was supplemented with both NADH and menadione **25** (Fig. 5E, trace c), there was significant reduction of cyt *c*_acet_. Rotenone only slightly decreased the rate of reduction of cyt *c*_acet_ (Fig. 5E, trace d), confirming that menadione **25** interacts at the flavin mononucleotide (FMN) site rather than at the Q-binding site. Superoxide dismutase (SOD), which dismutates O_2_^•-^ to O_2_ and H_2_O_2_, markedly decreased the rate of reduction of cyt *c*_acet_ (Fig. 5E, trace e).

BHM alone produced negligible O_2_^•-^ when supplemented with succinate (Fig. 5F, trace a) or menadione **25** (Fig. 5F, trace b). When BHM was supplemented with both succinate and menadione **25** (Fig. 5F, trace c), there was reduction of cyt *c*_acet_, which was attenuated by SOD (Fig. 5F, trace d). The rate of reduction of cyt *c*_acet_ was ∼9-fold lower with succinate than with NADH. Thus menadione **25** primarily generates O_2_^•-^ through redox cycling following its reduction by NADH at the FMN site of complex I. Accordingly, the release of menadiol **17** by MitoMenOH **5** and MitoMenOAc **6** should promote O_2_^•-^ generation within cells.

### 2.6 Uptake and reaction of prodrugs in cells

**Fig. 6:**
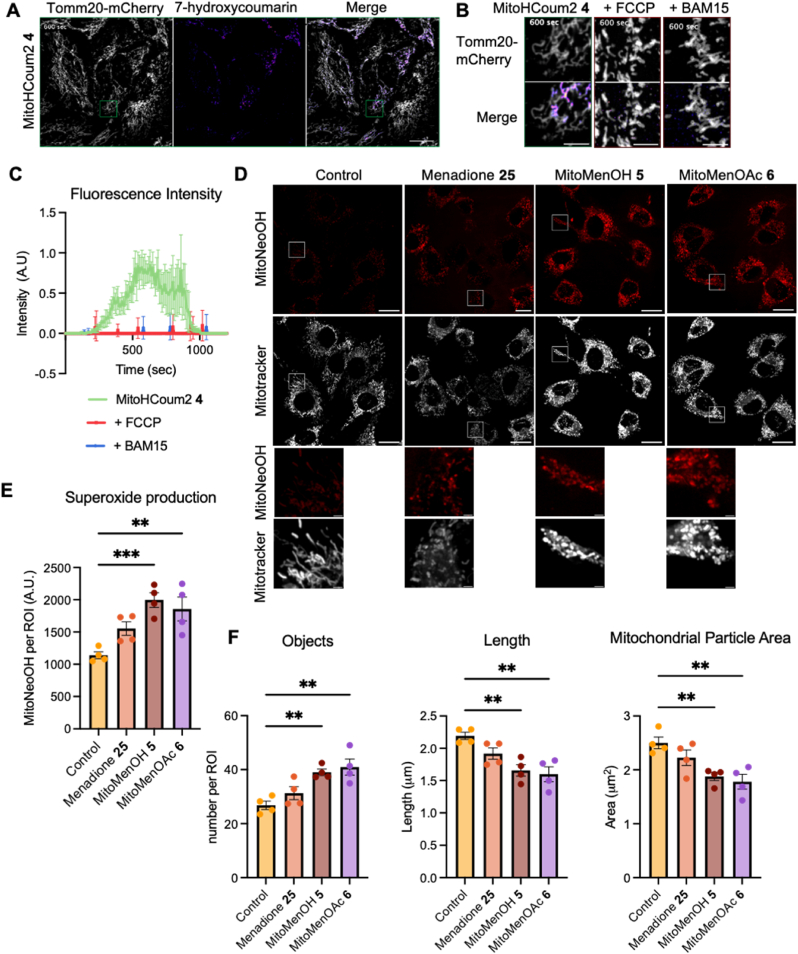
Time-course fluorescence of HeLa cells expressing Tomm20-mCherry incubated with MitoHCoum2 **4** and mitochondrial O_2_^•-^ production in C2C12 cells incubated with MitoMenOH **5** or MitoMenOAc **6** measured by live-cell confocal microscopy. (A) Cells were incubated with MitoHCoum2 **4** (3 μM) and the fluorescence of 7-hydroxycoumarin **9** measured by taking images every 10 s. The image presented is at 600 s (10 min). (B) Inset images of the experiment described in (A) ± pretreatment with FCCP or BAM15. Scale bars on all representative images are 20 μm and 2 μm for inset. (C) Fluorescence intensity of 7-hydroxycoumarin **9** measured over time, shown as absolute intensity normalised to Tomm20-mCherry fluorescence. Values are displayed in arbitrary units (A.U.) per region of interest (225 μm^2^) for each condition. (D) C2C12 cells were incubated with MitoMenOH **5** (3 μM) or MitoMenOAc **6** (3 μM) for 15 min and then with MitoNeoD (5 μM) and MitoTracker Deep Red (5 nM) for 20 min. Fluorescence intensity of MitoNeoOH, the oxidised product of MitoNeoD, was measured by live-cell confocal microscopy. Representative images show MitoNeoOH and MitoTracker Deep Red FM. (E) Fluorescence intensity of MitoNeoOH measured as absolute intensity with arbitrary units (A.U.) per region of interest (225 μm^2^) for each condition. (F) Mitochondrial object number, length and area per region of interest (225 μm^2^) for each condition. Data are means ± SEM of N = 3 for experiments (A), (B) and (C), and N = 4 for experiments (D), (E) and (F). p values were calculated using the one-way ANOVA test. ∗p < 0.05, ∗∗p < 0.01, ∗∗∗p < 0.001. (For interpretation of the references to colour in this figure legend, the reader is referred to the Web version of this article.)

To determine whether MitoHCoum2 **4**, MitoMenOH **5** and MitoMenOAc **6** accumulate within the mitochondria in cells, HeLa cells stably expressing Tomm20-mCherry to visualise mitochondria were incubated with MitoHCoum2 **4** and assessed by confocal fluorescence microscopy. MitoHCoum2 4 is unreactive in culture media unless supplemented with 10% fetal bovine serum (FBS) (which contains glutathione and esterases), in which case we observed slow breakdown (Fig. S6). After 1 h there was less than 10% conversion to the fluorescent payload. Therefore, MitoHCoum2-treated cells did not include FBS in the culture media. Following addition of MitoHCoum2 **4**, the fluorescence increased rapidly, reaching a maximum at ∼10 min after addition before decreasing to baseline by ∼20 min, as seen in the full image (Fig. 6A) and its corresponding 225 μm^2^ inset (Fig. 6B) taken at 10 min from the time-course video (Video 1), as well as in the fluorescence intensity time-course (Fig. 6C). The fluorescence signal colocalised with the mitochondria marker Tomm20-mCherry, indicating that MitoHCoum2 **4** accumulates in mitochondria and releases 7-hydroxycoumarin **9**. The subsequent loss of fluorescence indicates that 7-hydroxycoumarin **9** is diluted by diffusion out of mitochondria as was the case with isolated mitochondria (Fig. 4B). The uncouplers FCCP or BAM15 abolished fluorescence as seen in the insets (Fig. 6B) taken at 10 min from the time-course videos (Videos 2 and 3, respectively), as well as in the fluorescence intensity time-course (Fig. 6C), demonstrating that mitochondrial uptake of MitoHCoum2 **4** is membrane potential–dependent. These findings confirm that MitoHCoum2 **4** accumulates selectively in the mitochondria in cells and there, releases its payload.

We did not directly assess mGSH depletion with compounds **4**–**6** in cells due to the difficulty of rapidly isolating sufficient mitochondria from cultured cells without loss of GSH and in large enough amounts to assay accurately. However, we have demonstrated that compounds **4**–**6** rapidly react with GSH to form GS-adducts and so release their payloads (Figs. 2H and 3), that they accumulate in RLM and deplete mGSH in a membrane-potential-dependent manner (Fig. 4, Fig. 5), and that treating cells with MitoHCoum2 **4** leads to rapid localized release of its payload in the mitochondria (Fig. 6B and C). Selective depletion of mGSH by compounds **4**–**6** in cells is congruent with these results, and we have previously demonstrated such selective depletion in cultured cells with MitoCDNB **1** [2], which is a close structural analogue of compounds **4**–**6**.

To evaluate mitochondrial uptake of MitoMenOH **5** and MitoMenOAc **6** in cells, we assessed the release of menadione **25** indirectly by monitoring mitochondrial O_2_^•-^ production using MitoNeoD. MitoNeoD is a TPP-conjugated sensor that is oxidised by O_2_^•-^ to produce the fluorescent product MitoNeoOH [44]. C2C12 cells were incubated with MitoMenOH **5** or MitoMenOAc **6**, and then with MitoNeoD and MitoTracker Deep Red, labelling mitochondria. Both MitoMenOH **5** and MitoMenOAc **6** increased mitochondrial O_2_^•-^ production (Fig. 6D and E). Mitochondrial morphology was also analysed by measuring the number, length and area of mitochondria in randomly selected regions of interest (ROIs). Both MitoMenOH **5** and MitoMenOAc **6** resulted in fragmentation of the mitochondrial network (Fig. 6F). Together these findings demonstrate the membrane potential-driven uptake into cells followed by the release of the payload, which in the case of menadione **25** elevated O_2_^•-^ production.

### 2.7 Cytotoxicity of prodrugs

**Fig. 7:**
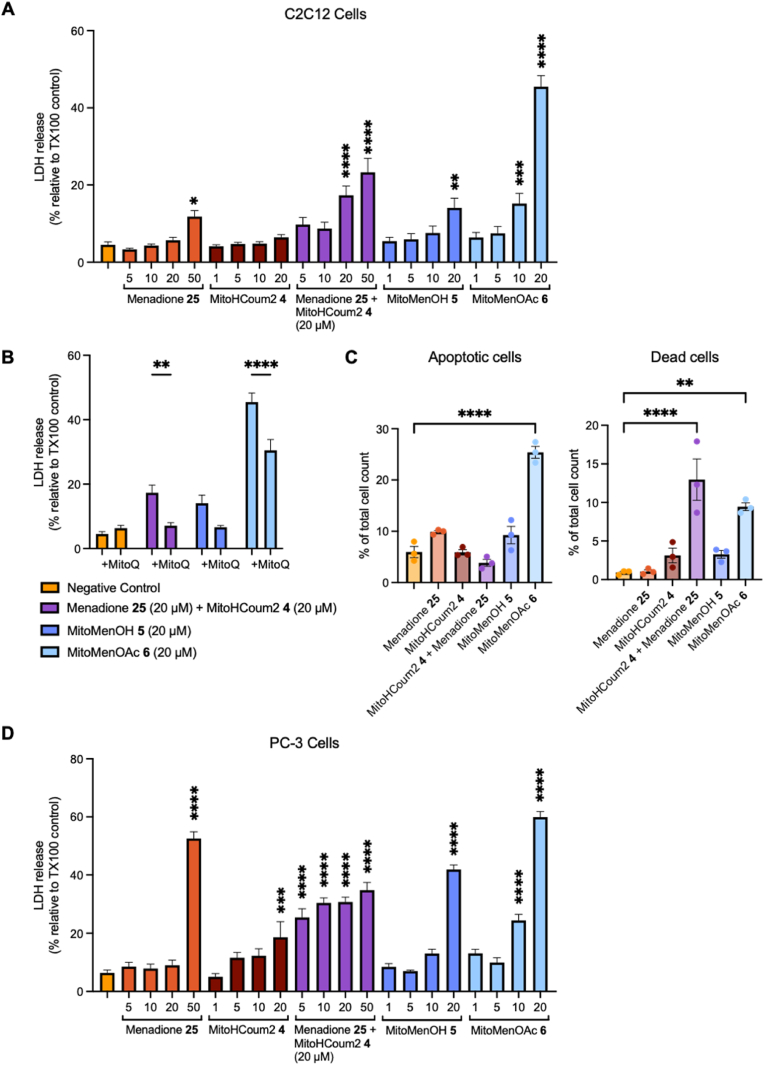
Cytotoxicity of prodrugs. (A) LDH release from C2C12 cells incubated with menadione **25**, MitoHCoum2 **4**, MitoMenOH **5** or MitoMenOAc **6** at different concentrations for 3 h, measured using the LDH Cytotoxicity Assay kit (Roche). (B) LDH release from C2C12 cells incubated with 20 μM of compound in the presence or absence of 1 μM MitoQ. (C) C2C12 cells were incubated with 20 μM of compound for 3 h, and cell viability was measured by flow cytometry using the Dead Cell Apoptosis Kit with Annexin V FITC and Propidium Iodide (PI) (Invitrogen). (D) LDH release from PC-3 cells incubated with menadione **25**, MitoHCoum2 **4**, MitoMenOH **5** or MitoMenOAc **6** at different concentrations for 3 h. In all experiments, the low-control consisted of 2 μL EtOH, equivalent to that used in all treatment conditions. LDH release data were normalised to a 1 % Triton X-100 addition, corresponding to 100 % LDH release. All data are presented as means ± SEM of N = 3. For experiments (A), (C) and (D), p values were calculated relative to the untreated cells (column 1) using the one-way ANOVA test. In experiment (B), p values were calculated by comparing equivalent conditions ± MitoQ using the two-way ANOVA test. ∗p < 0.05, ∗∗p < 0.01, ∗∗∗p < 0.001, ∗∗∗∗p < 0.0001.

To determine whether the combination of mGSH depletion and increased mitochondrial ROS generation led to enhanced cell death, we evaluated the cytotoxicity of the prodrugs towards C2C12 cells by measuring lactate dehydrogenase (LDH) release from dead cells. Treatment with menadione **25** below 50 μM resulted in negligible cell death (Fig. 7A). Incubation with MitoHCoum2 **4**, which depletes mGSH but does not release an oxidant, did not result in higher cell death compared to the untreated control (Fig. 7A). However, when 20 μM MitoHCoum2 **4** was supplemented with 20 μM or 50 μM menadione **25** (Fig. 7A), there was an increase in cell death relative to the untreated control and to the equivalent doses of the individual compounds. Addition of 1 μM MitoQ, a mitochondria-targeted antioxidant [45], attenuated LDH release (Fig. 7B), indicating that cell death was dependent on ROS production. MitoMenOH **5** and MitoMenOAc **6**, which both deplete mGSH and release menadione **25**, demonstrated cytotoxicity at 20 μM (Fig. 7A), which was also attenuated by addition of MitoQ (Fig. 7B). 20 μM MitoMenOH **5** demonstrated comparable cytotoxicity to 20 μM MitoHCoum2 supplemented with 20 μM menadione, while 20 μM MitoMenOAc **6** was significantly more cytotoxic. These results indicate that mGSH depletion and mitochondrial delivery of menadione **25** act synergistically to drive cytotoxicity. Interestingly, MitoMenOAc **6** was over two-fold more cytotoxic than MitoMenOH **5**. This perhaps reflects the shorter half-life of MenOAc **16** to produce monoacetate **18** than that of MenOH **15** to produce menadione **25**. The monoacetate **18** could potentially produce O_2_^•-^ production by redox cycling with its semiubiquinone form.

To corroborate our LDH release data, we also assessed C2C12 cell viability after incubation with 20 μM of our compounds by flow cytometry (Fig. 7C). Live, apoptotic and dead cells were distinguished using Annexin V FITC and Propidium Iodide (PI) staining (Fig. S7). Incubation with menadione **25**, MitoHCoum2 **4** or MitoMenOH **5** did not increase the proportion of apoptotic or dead cells relative to the untreated control (Fig. 7C). Notably, differences in cell number and media volume between the LDH release and flow cytometry experiments result in an approximately 10-fold lower amount of compound (in moles) per cell in the flow cytometry experiments. This disparity could account for the cytotoxicity of MitoMenOH **5** observed in the LDH assay but not detected by flow cytometry. When MitoHCoum2 **4** was supplemented with menadione **25**, there was a marked increase in the number of dead cells, but not apoptotic cells (Fig. 7C). Incubation with MitoMenOAc **6** resulted in a significant increase in both apoptotic and dead cells (Fig. 7C). These results suggest that MitoMenOAc **6** induces apoptosis by mGSH depletion and ROS generation.

We extended our investigation to the prostate cancer PC-3 cells, which contain higher GSH levels compared to most other cells, linked to their rapid growth [46]. Here, 20 μM MitoHCoum2 **4** was sufficient for cytotoxicity, suggesting that the PC-3 cells were more susceptible to mGSH depletion than the C2C12 cells (Fig. 7D). Similarly to the C2C12 cells, when 20 μM MitoHCoum2 **4** was supplemented with menadione **25** (Fig. 7D), cell death increased relative to the equivalent doses of the individual compounds (with the exception of 50 μM menadione). At 20 μM, MitoMenOH **5** and MitoMenOAc **6** were ∼two-fold and ∼three-fold more cytotoxic than MitoHCoum2 **4**, respectively, and significantly more cytotoxic than menadione **25** (Fig. 7D). Additionally, prodrugs **5** and **6** were more cytotoxic than MitoHCoum2 **4** supplemented with menadione **25** (Fig. 7D). These results demonstrate that cytotoxicity is enhanced when mGSH depletion is combined with menadione **25** release.

## 3 Conclusion

Overall, we have developed a new class of dual-action mitochondria-targeted prodrugs that deplete mGSH and simultaneously release an active phenolic payload within the mitochondria in cells. mGSH depletion combined with menadione **25** delivery is cytotoxic towards prostate cancer cells that upregulate GSH, illustrating the anti-cancer potential of MitoMenOH **5** and MitoMenOAc **6**. Although the design does not currently incorporate a cancer cell-targeting group, many cancer cells exhibit elevated GSH and a higher membrane potential [[10], [11], [12], [13], [14],[47], [48], [49]], which could result in greater uptake and reaction of prodrugs based on this scaffold compared to normal cells. The synthetic design incorporates the payload in the final step, allowing for straightforward preparation of prodrugs of several different payloads. We have proven here that the rate of cyclisation of the ethylenediamine linker can be altered by modifying the substituents on the nitrogen atoms, but it should also be possible to tune the rate of GSH reaction of the scaffold by modifying substituents on the 2,4-dinitrobenzene ring similar to our previous work [50]. Additionally, the ethylenediamine linker could be replaced with another linker, such as 4-amino benzyl alcohol to release amine-bearing payloads from a carbamate. We believe that this highly adaptable scaffold will be of value to the scientific community interested in targeting cancer by GSH depletion and ROS-based therapy.

## 4 Experimental

### 4.1 Chemical synthesis

All reagents and starting materials were obtained from commercial suppliers. All reactions were performed under an inert argon atmosphere in oven- or flame-dried flasks. Anhydrous solvents were purified using a PurSolv 500 MD solvent purification system and added via syringe. Column chromatography was performed either manually using fluorochem 60 Å (particle size 40– 63 μm) silica gel or, using a Biotage® IsoleraTM One Flash Chromatography system with Biotage® SNAP Ultra, Biotage® KIP or Agela silica gel cartridges (4, 10, 20, 35, 40 or 70 g). Purification and reaction kinetics by HPLC was performed using a Shimadzu LC-20AD Liquid Chromatograph fitted with an SPD-20A UV-VIS detector with absorbance measured at 254 and 280 nm. The column used for purification was a Luna Omega PS C18 (250 × 10 mm), with a solvent flow of 5 mL/min. The column used for analysis was a Luna Omega PS (250 × 4.6 mm), with a solvent flow of 1 mL/min. The mobile phase consisted of a mixture of HPLC-grade H_2_O (0.1 % TFA) and HPLC-grade acetonitrile. Melting points were determined using a Gallenkamp MFB.595.010 M equipped with an internal thermometer. Infrared spectra were recorded using a Shimadzu FTIR-8400S spectrometer. ^1^H, ^13^C and ^31^P NMR spectra were recorded on either 400 MHz (Bruker AVIII, Bruker AVIII HD or Bruker DPX) or 500 MHz (BrukerAVIII HD) spectrometers operating at 400, 101, and 162 MHz, respectively. Chemical shift values are reported in ppm relative to tetramethylsilane (*δ*_H_ 0.00 and *δ*_C_ 0.00) and referenced to the relevant deuterated solvent residual peaks. NMR data are reported in the format: chemical shift (integral, multiplicity [s = singlet, d = doublet, dd = doublet of doublets, t = triplet, td = triplet of doublets, q = quartet, quin = qn, m = multiplet, sept = septet, br = broad], coupling constant in Hz, assignment). Coupling constants are as observed and are not corrected to match other signals. ^13^C NMR data are reported to 2 d.p. to distinguish between observed signals that differ by less than 0.1 ppm. Assignment of ^1^H and ^13^C NMR signals was supported by analysis of DEPT, COSY and HSQC experiments. Mass spectra were recorded using a Bruker MicroTOFq High Resolution Mass Spectrometer coupled to a time-of-flight (TOF) analyser for positive or negative mode electrospray (ESI^+/−^) or atmospheric pressure chemical ionisation (APCI^+/−^).

#### 4.1.1 (4‐{[5’‐({2’’‐[Methyl({[(2‴‐oxo‐2H‐chromen‐7‴‐yl)oxy]carbonyl})amino]ethyl}sulfamoyl)‐2,4‐dinitrophenyl]amino}butyl)triphenylphosphonium chloride (MitoHCoum1) 3

Triphosgene (15.3 mg, 0.0524 mmol, 0.333 eq.) and anhydrous *N,N*-diisopropylethylamine (26.9 μL, 0.154 mmol, 1.00 eq.) were added to a solution of 7-hydroxycoumarin (25.0 mg, 0.154 mmol, 1.00 eq.) in dichloromethane (3.0 mL) and the reaction was stirred at room temperature for 1 h under argon. A solution of **20** (113 mg, 0.154 mmol, 1.00 eq.) in anhydrous dichloromethane (3 mL) was added, followed by anhydrous *N,N*-diisopropylethylamine (53.7 μL, 0.308 mmol, 2.00 eq.), and the reaction was stirred at room temperature for an additional 24 h under argon. The reaction was diluted with dichloromethane (15 mL) and washed with an aqueous solution of HCl (15 mL, 1 M). The aqueous layer was extracted with dichloromethane (2 × 15 mL). The organic layers were combined, dried with MgSO_4_ and the solvent was removed under reduced pressure. The crude material was purified by several rounds of column chromatography: first by normal phase column chromatography [SiO_2_, dichloromethane:methanol 0-20%], then by semi-preparative RP-HPLC [0.1% TFA/water: acetonitrile, 35-50%], and finally another round of normal phase column chromatography [SiO_2_, dichloromethane:methanol 0-20%] to afford **3** as a yellow hygroscopic foam (9.0 mg, 5%). NMR spectroscopy indicated a 1.2:1 mixture of diastereomers (rotamers) A:B. Ʋ_max_ (ATR): 1719 (C=O), 1613 (Aryl C=C) cm^−1^. ^1^H NMR (400 MHz, CDCl_3_): *δ*_H_ 8.94 (1H, t, *J* = 6.0 Hz, O_2_SNH^A^), 8.69 (1H, s, H-3′^B^), 8.67 (1H, t, *J* = 6.0 Hz, O_2_SNH^B^), 8.59 (1H, s, H-3′^A^), 8.48 (1H, t, *J* = 5.6 Hz, CH_2_N*H*^B^), 8.42 (1H, t, *J* = 5.8 Hz, CH_2_N*H*^A^), 8.08 (1H, s, H-6′^A^), 8.02 (1H, s, H-6′^B^), 7.88 – 7.61 (16H, m, Ph^A and B^, and H-4’’’^A and B^), 7.41 (1H, s, H-8’’’^B^), 7.39 (1H, s, H-8’’’^A^), 7.19 – 7.07 (4H, m, H-5’’’^A and B^, and H-6’’’^A and B^), 6.31 (1H, d, *J* = 9.4 Hz, H-3’’’^B^), 6.27 (1H, d, *J* = 9.4 Hz, H-3’’’^A^), 3.99 – 3.80 (4H, m, CH_2_-1^A and B^, and CH_2_-4^A and B^), 3.70 (2H, t, *J* = 6.1 Hz, CH_2_-2″^A^), 3.58 (2H, t, *J* = 5.6 Hz, CH_2_-2″^B^), 3.52 – 3.37 (4H, m, CH_2_-1″^A and^ ^B^), 3.22 (3H, s, CH_3_^B^), 3.08 (3H, s, CH_3_^A^), 2.05 (2H, m, CH_2_-2^A and B^), 1.81 (2H, m, CH_2_-3^A and B^). ^13^C NMR (101 MHz, CDCl_3_): *δ*_C_ 160.82 (C^A^), 160.79 (C^B^), 154.63 (C^B^), 154.57 (C^A^), 154.19 (C^B^), 153.82 (C^A^), 147.05 (C^B^), 146.95 (C^A^), 143.38 (CH^A or B^), 143.33 (CH^A or B^), 142.33 (C^A^), 142.11 (C^B^), 135.20 (d, *J* = 3.0 Hz, 3 x CH^A and B^), 133.83 (d, *J* = 10.1 Hz, 6 x CH^A and B^), 130.62 (d, *J* = 12.6 Hz, 6 x CH^A and B^), 130.07 (C^A and B^), 128.43 (CH^A or B^), 128.40 (CH^A or B^), 125.75 (CH^B^), 125.23 (CH^A^), 119.07 (CH^A^), 119.04 (CH^B^), 118.18 (d, *J* = 86.1 Hz, 3 x C^A and B^), 117.71 (CH^A^), 117.49 (CH^B^), 116.08 (C^A^), 116.04 (C^B^), 115.45 (CH^A or B^), 115.35 (CH^A or B^), 110.85 (CH^A^), 110.77 (CH^B^), 50.34 (CH_2_^B^), 49.79 (CH_2_^A^), 42.65 (CH_2_^A and B^), 41.84 (CH_2_^A and B^), 36.72 (CH_3_^B^), 36.01 (CH_3_^A^), 29.41 (d, *J* = 18.0 Hz, CH_2_^A and B^), 22.05 (d, *J* = 53.6 Hz, CH_2_^A and B^), 19.55 (d, *J* = 4.0 Hz, CH_2_^A and B^). ^31^P NMR (162 MHz, CD_3_CN): *δ*_P_ 24.47 (1P, s, PPh_3_^A or B^), 24.45 (1P, s, PPh_3_^A or B^). HRMS (ESI^+^): C_41_H_39_N_5_O_10_PS requires 824.2150 found 824.2161 (M^+^). Assignment of ^1^H and ^13^C NMR signals was supported by analysis of COSY, HSQC and DEPT experiments. Two signals corresponding to two C carbons were not identified in the ^13^C NMR spectrum, perhaps due to coincidence with other signals.

#### 4.1.2 [4‐({5‐[Methyl({2‐[methyl({[(2‐oxo‐2H‐chromen‐7-yl)oxy]carbonyl})amino]ethyl})sulfamoyl]‐2,4-dinitrophenyl}amino)butyl] triphenylphosphonium trifluoroacetate (MitoHCoum2) 4

Triphosgene (15.3 mg, 0.0524 mmol, 0.333 eq.) and anhydrous *N,N*-diisopropylethylamine (26.9 μL, 0.154 mmol, 1.00 eq.) were added to a solution of 7-hydroxycoumarin (25.0 mg, 0.154 mmol, 1.00 eq.) in anhydrous dichloromethane (1.5 mL) and the reaction was stirred at room temperature for 1 h under argon. A solution of **21** (138 mg, 0.154 mmol, 1.00 eq.) in anhydrous dichloromethane (1.5 mL) was added, followed by anhydrous *N,N*-diisopropylethylamine (53.7 μL, 0.308 mmol, 2.00 eq.), and the reaction was stirred at room temperature for an additional 24 h under argon. The reaction was diluted with dichloromethane (15 mL) and washed with an aqueous solution of HCl (15 mL, 1 M). The aqueous layer was extracted with dichloromethane (2 × 15 mL). The organic layers were combined, dried with MgSO_4_ and the solvent was removed under reduced pressure. The crude material was purified by column chromatography [SiO_2_, dichloromethane:methanol 0-20%], followed by semi-preparative RP-HPLC [0.1% TFA/water: acetonitrile, 35-45%] to afford **4** as a yellow hygroscopic foam (27.9 mg, 22 %). NMR spectroscopy indicated a 1.1:1 mixture of diastereomers (rotamers) A:B. Ʋ_max_ (ATR): 1719 (C=O), 1615 (Aryl C=C) cm^−1^. ^1^H NMR (400 MHz, CD_3_CN): *δ*_H_ 8.69 (1H, s, H-3′^A^), 8.68 (1H, s, H-3′^B^), 8.44 – 8.35 (1H, m, NH^A and B^), 7.90 – 7.79 (5H, m, Ph^A and B^), 7.74 – 7.62 (11H, m, Ph^A and B^ and H-4’’’^A and B^), 7.58 (1H, d, *J* = 8.4 Hz, H-5’’’^B^), 7.57 (1H, d, *J* = 8.4 Hz, H-5’’’^A^), 7.37 (1H, s, H-6′^A^), 7.34 (1H, s, H-6′^B^), 7.15 – 7.09 (2H, m, H-8’’’^A and B^ and H-6’’’^A or B^), 7.08 (1H, dd, *J* = 8.4, 2.2 Hz, H-6’’’^A or B^), 6.33 (1H, d, *J* = 9.6 Hz, H-3’’’^B^), 6.32 (1H, d, *J* = 9.5 Hz, H-3’’’^A^), 3.69 – 3.45 (6H, m, H-4^A and B^, H-1″^A and B^ and H-2″^A and B^), 3.26 – 3.14 (2H, m, H-1^A and B^), 3.10 (3H, s, O_2_SNCH_3_^A or B^ or O_2_CNCH_3_^A or B^), 2.98 (6H, m, O_2_SNCH_3_^A and B^ and O_2_CNCH_3_^A or B^, or, O_2_SNCH_3_^A or B^ and O_2_CNCH_3_^A and B^), 1.92 – 1.81 (2H, m, H-2^A and B^), 1.80 – 1.66 (2H, m, H-3^A and B^). ^13^C NMR (101 MHz, CD_3_CN): 161.23 (C^A and B^), 155.54 (C^A and B^), 155.21 (C^A or B^), 155.14 (C^A or B^), 155.08 (C^A or B^), 154.63 (C^A or B^), 147.45 (C^A and B^), 144.41 (CH^A and B^), 140.41 (C^A or B^), 140.35 (C^A or B^), 136.17 (d, *J* = 3.1 Hz, 6 x CH^A and B^), 135.42 (C^A or B^), 135.35 (C^A or B^), 134.62 (d, *J* = 10.0 Hz, 6 x CH^A and B^), 131.73 (C^A and B^), 131.28 (d, *J* = 12.6 Hz, 6 x CH^A and B^), 129.85 (CH^A and B^), 126.98 (CH^A and B^), 119.63 (CH^A or B^), 119.32 (CH^A or B^), 119.06 (d, *J* = 86.4 Hz, 6 x C^A and B^), 118.48∗ (CH^A or B^), 118.17∗ (CH^A or B^), 117.46 (C^B^), 117.36 (C^A^), 116.43 (CH^B^), 116.36 (CH^A^), 111.02 (CH^A or B^), 110.92 (CH^A or B^), 49.42 (CH_2_^B^), 48.72 (CH_2_^A^), 47.55 (CH_2_^A or B^), 47.50 (CH_2_^A or B^), 42.94 (CH_2_^A and B^), 35.82 (CH_3_^A^), 35.77 (CH_3_^B^), 35.64 (CH_3_^B^), 35.47 (CH_3_^A^), 29.89 (d, *J* = 16.8 Hz, CH_2_^A and B^), 22.35 (d, *J* = 52.2 Hz, CH_2_^B^), 22.34 (d, *J* = 52.2 Hz, CH_2_^A^), 20.44 (CH_2_^A and B^). ^31^P NMR (162 MHz, CD_3_CN): *δ*_P_ 23.62 (1P, s, PPh_3_). ^19^F NMR (377 MHz, CD_3_CN): *δ*_F_ −76.59 (3F, s, TFA). HRMS (ESI^+^): C_42_H_41_N_5_O_10_PS requires 838.2306 found 838.2208 (M^+^). Assignment of ^1^H and ^13^C NMR signals was supported by analysis of COSY, HSQC and DEPT experiments. ∗Signals were hidden behind the solvent signal in the ^13^C NMR spectrum, but were observed in the DEPT spectrum. The signals corresponding to the TFA^−^ anion were not observed in the ^13^C NMR spectrum.

#### 4.1.3 {4‐[(5’‐{[2’’‐({[(4‴‐Hydroxy‐3‴‐methylnaphthalen‐1‴‐yl)oxy]carbonyl}(methyl)amino)ethyl](methyl)sulfamoyl}‐2′,4′‐dinitrophenyl)amino]butyl}triphenylphosphonium chloride (MitoMenOH) 5

An aqueous solution of HCl (0.4 mL, 6 M) was added to a solution of **6** (75.0 mg, 0.0757 mmol) in methanol (0.4 mL) and the reaction was stirred at reflux for 24 h under argon. The reaction was allowed to cool to room temperature and the solvent was removed under reduced pressure to afford **5** as an orange hygroscopic foam (66.9 mg, quant.). NMR spectroscopy indicated a 1:1 mixture of diastereomers (rotamers) A:B. Ʋ_max_ (ATR): 1715 (C=O), 1614 (Aryl C=C) cm^−1^. ^1^H NMR (400 MHz, CD_3_CN): *δ*_H_ 8.67 (1H, s, H-3′^A or B^), 8.61 (1H, s, H-3′^A or B^), 8.47 (1H, s, OH^A or B^), 8.36 – 8.26 (2H, m, NH^A or B^ and H-5’’’^A and B^ or H-8’’’^A and B^), 8.21 (1H, s, OH^A or B^), 8.10 (1H, t, *J* = 4.7 Hz, NH^A or B^), 7.86 – 7.59 (16H, m, Ph^A and B^ and H-5’’’^A and B^ or H-8’’’^A and B^), (7.41 – 7.32 (2H, m, H-6’’’^A and B^ and H-7’’’^A and B^), 7.31 (1H, s, H-6′^A or B^), 7.24 (1H, s, H-6′^A or B^), 7.00 (1H, s, H-2’’’^A or B^), 6.94 (1H, s, H-2’’’^A or B^), 3.70 – 3.62 (2H, m, CH_2_-1″^A and B^ or CH_2_-2″^A or B^), 3.57 – 3.50 (2H, m, CH_2_-1″^A and B^ or CH_2_-2″^A or B^), 3.45 – 3.35 (2H, m, CH_2_-1^A or B^), 3.28 (1H, q, *J* = 6.0 Hz, CH_2_-4^A or B^), 3.26 – 3.15 (5H, m, CH_2_-1^A or B^ and O_2_SNCH_3_^A or B^ or O_2_CNCH_3_^A or B^), 3.09 (3H, s, O_2_SNCH_3_^A or B^ or O_2_CNCH_3_^A or B^), 3.05 (2H, q, *J* = 6.1 Hz, CH_2_-4^A or B^), 3.02 (3H, s, O_2_SNCH_3_^A or B^ or O_2_CNCH_3_^A or B^), 2.97 (3H, s, O_2_SNCH_3_^A or B^ or O_2_CNCH_3_^A or B^), 2.36 (2H, s, PhC*H*_*3*_^A or B^), 2.35 (2H, s, PhC*H*_*3*_^A or B^), 1.72 – 1.61 (2H, m, CH_2_-2), 1.60 – 1.48 (2H, m, CH_2_-3). ^13^C NMR (101 MHz, CD_3_CN): *δ*_C_ 156.13 (C^A or B^), 155.80 (C^A or B^), 148.84 (C^A or B^), 148.66 (C^A or B^), 147.32 (C^A or B^), 147.04 (C^A or B^), 140.85 (C^A or B^), 140.63 (C^A and B^), 140.25 (C^A or B^), 135.99 (d, *J* = 3.0 Hz, 3 x CH^A and B^), 135.22 (C^A or B^), 134.94 (C^A or B^), 134.73 (d, *J* = 10.1 Hz, 6 x CH^A or B^), 134.68 (d, *J* = 10.0 Hz, 6 x CH^A or B^), 131.52 (C^A or B^), 131.38 (C^A or B^), 131.19 (d, *J* = 12.6 Hz, 6 x CH^A and B^), 127.62 (C^A or B^), 127.51 (C^A or B^), 127.30 (C^A or B^), 127.17 (C^A or B^), 127.11 (CH^A or B^), 126.94 (CH^A or B^), 126.31 (CH^A or B^), 126.28 (CH^A or B^), 126.08 (CH^A and B^), 123.80 (CH^A or B^), 123.55 (CH^A or B^), 122.63 (CH^A or B^), 122.47 (CH^A or B^), 122.24 (CH^A or B^), 121.75 (CH^A or B^), 119.90 (C^A or B^), 119.51 (C^A or B^), 119.25 (d, *J* = 86.3 Hz, 3 x C^A or B^), 119.21 (d, *J* = 86.4 Hz, 3 x C^A or B^), 118.71 (CH^A or B^), 118.68 (CH^A or B^), 48.81 (CH_2_^A or B^), 48.63 (CH_2_^A or B^), 47.70 (CH_2_^A or B^), 47.65 (CH_2_^A or B^), 42.64 (CH_2_^A or B^), 42.57 (CH_2_^A or B^), 36.11 (CH_3_^A or B^), 36.07 (CH_3_^A or B^), 36.05 (CH_3_^A or B^), 35.90 (CH_3_^A or B^), 29.46 (d, *J* = 16.5 Hz, CH_2_^A or B^), 29.36 (d, *J* = 16.8 Hz, CH_2_^A or B^), 22.19 (d, *J* = 51.4 Hz, CH_2_^A or B^), 22.01 (d, *J* = 51.7 Hz, CH_2_^A or B^), 20.20 (CH_2_^A and B^), 17.23 (CH_3_^A and B^). ^31^P NMR (162 MHz, CD_3_CN): *δ*_P_ 23.86 (1P, s, PPh_3_^A and B^). HRMS (ESI^+^): C_44_H_45_N_5_O_9_PS requires 850.2670 found 850.2680 (M^+^). Assignment of ^1^H and ^13^C signals was supported by analysis of COSY, HSQC and DEPT experiments. ∗Signals were hidden behind the solvent signal in the ^13^C NMR spectrum but were observed in the DEPT spectrum from which their shift was estimated.

#### 4.1.4 (4‐{[5’‐({2’’‐[({[4‴‐(Acetyloxy)‐3‴‐methylnaphthalen‐1‴‐yl]oxy}carbonyl(methyl) amino]ethyl}(methyl)sulfamoyl)‐2′,4′-dinitrophenyl]amino}butyl) triphenylphosphonium trifluoroacetate (MitoMenOAc) 6

Triphosgene (50.2 mg, 0.169 mmol, 0.330 eq.) and anhydrous *N,N*-diisopropylethylamine (88.7 μL, 0.508 mmol, 1.00 eq.) were added to a solution of **15** (110 mg, 0.508 mmol, 1.00 eq.) in anhydrous dichloromethane (2.5 mL). The reaction was stirred at room temperature for 1 h under argon. A solution of **21** (454 mg, 0.508 mmol, 1.00 eq.) and anhydrous *N,N*-diisopropylethylamine (177 μL, 1.02 mmol, 2.00 eq.) in dichloromethane (2.5 mL) was added to the reaction, which was stirred at room temperature for an additional 3 h under argon. The reaction was diluted with dichloromethane (20 mL). The organic layer was washed with a saturated solution of NH_4_Cl (20 mL), and the aqueous layer was extracted with dichloromethane (2 × 15 mL). The organic layers were combined, dried with MgSO_4_ and the solvent was removed under reduced pressure. The crude material was purified by semi-preparative RP-HPLC [0.1% TFA/water: acetonitrile, 10-90%] to afford **6** as an orange hygroscopic foam (179 mg, 36%). NMR spectroscopy indicated a 1.3:1 mixture of diastereomers (rotamers) A:B. Ʋ_max_ (ATR): 1719 (C=O), 1685 (C=O) cm^−1^. ^1^H NMR (400 MHz, CD_3_CN): *δ*_H_ 8.69 (1H, s, H-3′^A^), 8.65 (1H, s, H-3′^B^), 8.39 (1H, t, *J* = 5.9 Hz, NH^A^), 8.25 (1H, t, *J* = 5.8 Hz, NH^B^), 8.00 – 7.77 (5H, m, H-5’’’^A and B^, H-8’’’^A and B^ and Ph^A and B^), 7.74 – 7.60 (12H, m, Ph^A and B^), 7.57 – 7.42 (2H, m, H-6’’’^A and B^ and H-7’’’^A and B^), 7.32 (1H, s, H-6′^B^), 7.32 (1H, s, H-6′^A^), 7.19 (1H, s, H-2’’’^A^), 7.18 (1H, s, H-2’’’^B^), 3.75 (2H, t, *J* = 6.0 Hz, CH_2_-2″^A or B^), 3.67 (2H, t, *J* = 5.8 Hz, CH_2_-2″^A or B^), 3.63 – 3.51 (2H, m, CH_2_-1″^A and B^), 3.41 (2H, q, *J* = 6.4 Hz, CH_2_-4^A or B^), 3.30 (2H, q, *J* = 6.3 Hz, CH_2_-4^A or B^), 3.26 (3H, s, O_2_SNCH_3_^A^ or O_2_CNCH_3_^A^), 3.23 – 3.08 (2H, m, CH_2_-1^A and B^), 3.06 (3H, s, O_2_SNCH_3_^B^ or O_2_CNCH_3_^B^), 3.02 (3H, s, S O_2_SNCH_3_^B^ or O_2_CNCH_3_^B^), 3.00 (3H, s, O_2_SNCH_3_^A^ or O_2_CNCH_3_^A^), 2.46 (3H, s, O_2_CCH_3_^A and B^), 2.27 (3H, s, PhC*H*_*3*_^A and B^), 1.75 (2H, p, *J* = 6.6 Hz, CH_2_-2^A and B^), 1.64 (2H, p, *J* = 7.8 Hz, CH_2_-3^A and B^). ^13^C NMR (101 MHz, CD_3_CN): *δ*_C_ 170.51 (C^B^), 170.41 (C^A^), 155.92 (C^A^), 155.36 (C^B^), 147.41 (C^A^), 147.25 (C^B^), 145.99 (C^A^), 145.91 (C^B^), 142.86 (C^A or B^), 142.78 (C^A or B^), 140.48 (C^B^), 140.39 (C^A^), 136.14 (d, *J* = 2.9 Hz, 3 x CH^A and B^), 135.40 (C^A^), 135.30 (C^B^), 134.61 (d, *J* = 10.0 Hz, 6 x CH^A and B^), 131.68 (C^A^), 131.64 (C^B^), 131.26 (d, *J* = 12.6 Hz, 6 x CH^A and B^), 128.67 (C^A and B^), 128.21 (CH^A and B^), 127.93 (C^B^), 127.80 (C^A^), 127.71 (C^A or B^), 127.67 (C^A or B^), 127.11 (CH^A and B^), 127.05 (CH^B^), 126.99 (CH^A^), 122.99 (CH^A^), 122.67 (CH^B^), 122.18 (CH^B^), 122.06 (CH^A^), 122.02 (CH^B^), 121.99 (CH^A^), 119.08 (d, *J* = 86.8 Hz, 3 x C^A and B^), 118.41∗ (CH^B^), 118.29∗ (CH^A^), 49.22 (CH_2_^B^), 48.79 (CH_2_^A^), 47.66 (CH_2_^B^), 47.52 (CH_2_^A^), 42.75 (CH_2_^A^), 42.67 (CH_2_^B^), 35.96 (CH_3_^B^), 35.89 (CH_3_^A^), 35.87 (CH_3_^B^), 35.64 (CH_3_^A^), 29.65 (d, *J* = 16.7 Hz, CH_2_^A and B^), 22.46 (d, *J* = 52.1 Hz, CH_2_^A or B^), 22.38 (d, *J* = 51.8 Hz, CH_2_^A or B^), 20.87 (CH_3_^B^), 20.86 (CH_3_^A^), 20.31 (CH_2_^A and B^), 16.50 (CH_3_^A and B^). ^31^P NMR (162 MHz, CD_3_CN): *δ*_P_ 23.69 (1P, s, PPh_3_^A^), 23.67 (1P, s, PPh_3_^B^). ^19^F NMR (377 MHz, CD_3_CN): *δ*_F_ −75.18 (3F, s, TFA). HRMS (ESI^+^): C_46_H_47_N_5_O_10_PS requires 892.2776 found 892.2791 (M^+^). Assignment of ^1^H and ^13^C NMR signals was supported by analysis of COSY, HSQC and DEPT experiments. ∗Signals were hidden behind the solvent signal in the ^13^C NMR spectrum but were observed in the DEPT spectrum. The signals corresponding to the TFA^−^ anion were not observed in the ^13^C NMR spectrum.

#### 4.1.5 2‐Oxo‐2H‐chromen‐7‐yl N‐(2‐azaniumylethyl)‐N‐methylcarbamate trifluoroacetate (HCoum1) 7

Trifluoroacetic acid (0.4 mL) was added to a solution of **23** (15.0 mg, 0.0414 mmol, 1.00 eq.) in anhydrous dichloromethane (0.4 mL) and the reaction was stirred at room temperature for 1 h under argon. The solvent was removed under reduced pressure to afford **7** as a white hygroscopic foam (16.1 mg, quant.). NMR spectroscopy indicated a 1.8:1 mixture of diastereomers (rotamers) A:B. Ʋmax (ATR): 1691 (C=O), 1619 (Aryl C=C) cm-1. 1H NMR (400 MHz, CD_3_OD): *δ*_H_ 7.96 (1H, d, *J* = 9.6 Hz, H-4^A and B^), 7.67 (1H, d, *J* = 8.5 Hz, H-5^A and B^), 7.29 – 7.23 (1H, m, H-8^A and B^), 7.19 (1H, dd, *J* = 8.5, 2.2 Hz, H-6^A and B^), 6.41 (1H, d, *J* = 9.6 Hz, H-3^A and B^), 3.80 (2H, t, *J* = 6.1 Hz, CH_3_NC*H*_*2*_^B^), 3.68 (2H, t, *J* = 5.8 Hz, CH_3_NC*H*_*2*_^A^), 3.26 (2H, t, *J* = 6.2 Hz, NH_3_C*H*_*2*_^B^), 3.21 (2H, t, *J* = 5.8 Hz, NH_3_C*H*_*2*_^A^), 3.18 (3H, s, CH_3_^A^), 3.07 (3H, s, CH_3_^B^). ^13^C NMR (101 MHz, CD_3_OD): *δ*_C_ 162.40 (C^A and B^), 156.47 (C^A and B^), 155.84 (C^A and B^), 155.33 (C^A and B^), 145.10 (CH^A and B^), 130.11 (CH^A and B^), 119.86 (CH^B^), 119.73 (CH^A^), 118.01 (C^A and B^), 116.46 (CH^B^), 116.39 (CH^A^), 111.35 (CH^B^), 111.26 (CH^A^), 39.01 (CH_2_^A and B^), 38.87 (CH_2_^A and B^), 35.51 (CH_3_^A and B^). ^19^F NMR (377 MHz, CD_3_OD): *δ*_F_ −76.87 (3F, s, TFA). HRMS (ESI^+^): C_13_H_15_N_2_O_4_ requires 263.1026 found 263.1027 (M^+^). Assignment of ^1^H and ^13^C NMR signals was supported by analysis of COSY, HSQC and DEPT experiments. The signals corresponding to the TFA^−^ anion were not observed in the ^13^C NMR spectrum.

#### 4.1.6 2‐Oxo‐2H‐chromen‐7‐yl N‐methyl‐N‐[2‐(methylazaniumyl)ethyl]carbamate trifluoroacetate (HCoum2) 8

Trifluoroacetic acid (0.74 mL) was added to a solution of **24** (23.8 mg, 0.0736 mmol, 1.00 eq.) in anhydrous dichloromethane (0.7 mL) and the reaction was stirred at room temperature for 1 h under argon. The solvent was removed under reduced pressure to afford **8** as a white hygroscopic solid (23.7 mg, quant.). NMR spectroscopy indicated a 3:1 mixture of diastereomers (rotamers) A:B. Ʋ_max_ (ATR): 1725 (C=O), 1700 (C=O), 1618 (Aryl C=C) cm^−1^. ^1^H NMR (400 MHz, CDCl_3_): *δ*_H_ 9.38 (2H, br s, NH_2_^B^), 9.20 (2H, br s, NH_2_^A^), 7.68 (1H, d, *J* = 9.6 Hz, H-4^A and B^), 7.46 (1H, d, *J* = 8.5 Hz, H-5^A and B^), 7.18 (1H, d, *J* = 2.2 Hz, H-8^A and B^), 7.11 (1H, dd, *J* = 8.5, 2.2 Hz, H-6^A and B^), 6.37 (1H, d, *J* = 9.5 Hz, H-4^A and B^), 3.79 [2H, t, *J* = 5.4 Hz, O_2_CN(CH_3_)C*H*_*2*_^B^], 3.68 [2H, t, *J* = 5.4 Hz, O_2_CN(CH_3_)C*H*_*2*_^A^], 3.28 – 3.16 (2H, m, CH_3_NH_2_*CH*_*2*_^A and B^), 3.11 (3H, s, O_2_CNCH_3_^A^), 3.00 (3H, s, O_2_CNCH_3_^B^), 2.70 (3H, s, NH_2_CH_3_^B^), 2.67 (3H, s, NH_2_CH_3_^A^). ^19^F NMR (377 MHz, CDCl_3_): *δ*_F_ −75.66 (3F, s, TFA). ^13^C NMR (101 MHz, CDCl_3_): *δ*_C_ 160.61 (C^A and B^), 155.15 (C^A and B^), 154.44 (C^A and B^), 153.69 (C^A and B^), 143.29 (CH^B^), 143.10 (CH^A^), 128.50 (CH^A and B^) 118.68 (CH^A^), 118.46 (CH^B^), 116.44 (C^A and B^), 115.84 (CH^A^), 115.60 (CH^B^), 110.56 (CH^A^), 110.31 (CH^B^), 47.27 (CH_2_^A^), 47.12 (CH_2_^B^), 46.03 (CH_2_^A^), 45.60 (CH_2_^B^), 35.38 (CH_3_^B^), 35.13 (CH_3_^A^), 33.47 (CH_3_^B^), 33.28 (CH_3_^A^). HRMS (ESI^+^): C_14_H_17_N_2_O_4_ requires 277.1183 found 277.1175 (M^+^). Assignment of ^1^H and ^13^C NMR signals was supported by analysis of COSY, HSQC and DEPT experiments. The signals corresponding to the TFA^−^ anion were not observed in the ^13^C NMR spectrum.

#### 4.1.7 [4‐({5’‐[(2’’‐{Methyl[(2‴‐oxo‐2H‐chromen‐7‴‐yl)carbamoyl]amino}ethyl)sulfamoyl]‐2,4‐dinitrophenyl}amino)butyl]triphenylphosphonium hexafluorophosphate (MitoACoum1) 10

Anhydrous phosgene solution (15% wt.% in toluene) (89 μL, 0.124, 1.10 eq.) and triethylamine (31.7 μL, 0.226 mmol, 2.00 eq.) were added to a solution of 7-aminocoumarin **14** (18.2 mg, 0.113 mmol, 1.00 eq.) in anhydrous dichloromethane (1.1 mL) and the reaction was stirred at 0 °C for 1 h under argon. The solvent was removed under reduced pressure. To the solid residue was added a solution of **20** (88.3 mg, 0.113, 1.00 eq.) in anhydrous acetonitrile (1.1 mL), followed by triethylamine (31.7 μL, 0.226 mmol, 2.00 eq.) and the reaction was stirred at room temperature for 24 h under argon. The solvent was removed under reduced pressure and the solid residue was re-dissolved in dichloromethane (15 mL). The organic layer was washed with an aqueous solution of HCl (15 mL, 1 M) and the aqueous layer was extracted with dichloromethane (3 × 10 mL). The organic layers were combined, dried with MgSO_4_ and the solvent was removed under reduced pressure. The crude material was purified by column chromatography [SiO_2_, dichloromethane:methanol 0-30%] to afford **10** as a yellow hygroscopic foam (20.7 mg, 19%). Ʋ_max_ (ATR): 1615 (C=O) cm^−1^. ^1^H NMR (400 MHz, CD_3_CN): *δ*_H_ 8.61 (1H, s, H-3′), 8.42 (1H, t, *J* = 5.8 Hz, CH_2_NH), 7.89 – 7.78 (3H, m, Ph), 7.74 (1H, d, *J* = 9.5 Hz, H-4‴), 7.72 – 7.59 (12H, m, Ph), 7.51 (1H, s, H-6′), 7.48 (1H, d, *J* = 2.1 Hz, H-8‴), 7.36 (1H, d, *J* = 8.5 Hz, H-5‴), 7.19 (1H, s, OCNH), 7.12 (1H, dd, *J* = 8.5, 2.1 Hz, H-6‴), 6.47 (1H, t, *J* = 4.8 Hz, O_2_SNH), 6.21 (1H, d, *J* = 9.4 Hz, H-3‴), 3.53 (2H, q, *J* = 6.6 Hz, CH_2_-4), 3.47 – 3.40 (4H, m, CH_2_-1″ and CH_2_-2″), 3.32 – 3.18 (2H, m, CH_2_-1), 2.90 (3H, s, CH_3_), 1.90 (2H, m, CH_2_-2), 1.82 – 1.69 (2H, m, CH_2_-3). ^13^C NMR (101 MHz, CD_3_CN): *δ*_C_ 161.79 (C), 155.96 (C), 155.75 (C), 148.03 (C), 144.61 (CH), 144.55 (C), 141.66 (C), 136.16 (d, *J* = 3.1 Hz, 3 x CH), 134.64 (d, *J* = 10.0 Hz, 6 x CH), 134.15 (C), 131.27 (d, *J* = 12.6 Hz, 6 x CH), 131.24 (C), 129.27 (CH), 127.90 (CH), 119.05 (d, *J* = 86.6 Hz, 3 x C), 118.25 (CH), 115.98 (CH), 114.59 (C), 114.38 (CH), 106.28 (CH), 49.26 (CH_2_), 43.08 (CH_2_), 42.72 (CH_2_), 35.90 (CH_3_), 29.82 (d, *J* = 16.9 Hz, CH_2_), 22.38 (d, *J* = 52.2 Hz, CH_2_), 20.47 (d, *J* = 3.8 Hz, CH_2_). ^31^P NMR (162 MHz, CD_3_CN): *δ*_P_ 23.64 (1P, s, PPh_3_), −144.62 (1P, sept, *J* = 706.3 Hz, PF_6_). ^19^F NMR (377 MHz, CD_3_CN): *δ*_F_ −72.91 (6F, d, *J* = 707.3 Hz, PF_6_). HRMS (ESI^+^): C_41_H_40_N_6_O_9_PS requires 823.2310 found 823.2314 (M^+^). Assignment of ^1^H and ^13^C NMR signals was supported by analysis of COSY, HSQC and DEPT experiments.

#### 4.1.8 [4‐({5’‐[Methyl(2’’‐{methyl[(2‴‐oxo‐2H‐chromen‐7‴-yl)carbamoyl]amino}ethyl) sulfamoyl]‐2,4-dinitrophenyl}amino)butyl]triphenylphosphonium hexafluorophosphate (MitoACoum2) 11

Triphosgene (6.1 mg, 0.0207 mmol, 0.333 eq.) and anhydrous *N,N*-diisopropylethylamine (10.8 μL, 0.0621 mmol, 1.00 eq.) were added to a solution of 7-aminocoumarin **14** (10.0 mg, 0.0621 mmol, 1.00 eq.) in anhydrous dichloromethane (0.6 mL) and the reaction was stirred at room temperature for 1 h under argon. A solution of **21** (55.5 mg, 0.0621 mmol, 1.00 eq.) in anhydrous dichloromethane (0.6 mL) was added, followed by anhydrous *N,N*-diisopropylethylamine (21.6 μL, 0.124 mmol, 2.00 eq.), and the reaction was stirred at room temperature for an additional 24 h under argon. The reaction was diluted with dichloromethane (15 mL) and washed with an aqueous solution of HCl (15 mL, 1 M). The aqueous layer was extracted with dichloromethane (2 × 15 mL). The organic layers were combined, dried with MgSO_4_ and the solvent was removed under reduced pressure. The crude material was purified by column chromatography [SiO_2_, dichloromethane:methanol 0-30%] to afford **11** as a yellow hygroscopic foam (16.8 mg, 29 %). Ʋ_max_ (ATR): 1727 (C=O), 1614 (Aryl C=C) cm^−1^. ^1^H NMR (400 MHz, CD_3_CN): *δ*_H_ 8.63 (1H, s, H-3′), 8.22 (1H, t, *J* = 5.8 Hz, CH_2_*NH*), 7.87 – 7.80 (3H, m, Ph), 7.76 (1H, d, *J* = 9.5, H-3‴), 7.72 – 7.64 (12H, m, Ph), 7.60 (1H, d, *J* = 2.1 Hz, H-8‴), 7.39 (1H, d, *J* = 8.5 Hz, H-5‴), 7.36 (1H, s, H-6′), 7.19 (1H, s, CONH), 7.15 (1H, dd, *J* = 8.5, 2.1 Hz, H-6‴), 6.21 (1H, d, *J* = 9.5 Hz, H-4′), 3.61 – 3.51 (4H, m, CH_2_-1″ and CH_2_-2″), 3.48 (2H, q, *J* = 6.5 Hz, CH_2_-4), 3.28 – 3.18 (2H, m, CH_2_-1), 3.07 (3H, s, O_2_SNCH_3_ or OCNCH_3_), 2.96 (3H, s, O_2_SNCH_3_ or OCNCH_3_), 1.87 (2H, qn, *J* = 7.0 Hz, CH_2_-2), 1.80 – 1.68 (2H, m, CH_2_-3). ^13^C NMR (101 MHz, CD_3_CN): *δ*_C_ 161.88 (C), 155.81 (C), 155.78 (C), 147.34 (C), 144.73 (C), 144.71 (CH), 140.67 (C), 136.16 (d, *J* = 3.1 Hz, 3 x CH), 135.02 (C), 134.64 (d, *J* = 10.1 Hz, 6 x CH), 131.39 (C), 131.27 (d, *J* = 12.6 Hz, 6 x CH), 129.28 (CH), 126.94 (CH), 119.48 (d, *J* = 85.7 Hz, 3 x C), 118.86 (CH), 116.18 (CH), 114.57 (C), 114.30 (CH), 106.44 (CH), 48.97 (CH_2_), 47.53 (CH_2_), 43.02 (CH_2_), 36.56 (CH_3_), 35.59 (CH_3_), 29.90 (d, *J* = 17.0 Hz, CH_2_), 22.39 (d, *J* = 52.1 Hz, CH_2_), 20.49 (d, *J* = 3.8 Hz, CH_2_). ^31^P NMR (162 MHz, CD_3_CN): *δ*_P_ 23.62 (1P, s, PPh_3_), −144.63 (1P, sept, *J* = 706.0 Hz, PF_6_). ^19^F NMR (377 MHz, CD_3_CN): *δ*_F_ −72.93 (6F, d, *J* = 707.4 Hz, PF_6_). HRMS (ESI^+^): C_42_H_42_N_6_O_9_PS requires 837.2466 found 837.2477 (M^+^). Assignment of ^1^H and ^13^C NMR signals was supported by analysis of COSY, HSQC and DEPT experiments.

#### 4.1.9 3‐(2‐Azaniumylethyl)‐3‐methyl‐1‐(2‐oxo‐2H‐chromen‐7‐yl)urea trifluoroacetate (ACoum1) 12

Trifluoroacetic acid (0.6 mL) was added to a solution of **29** (20.0 mg, 0.0553 mmol, 1.00 eq.) in anhydrous dichloromethane (0.6 mL) and the reaction was stirred at room temperature for 0.5 h under argon. The solvent was removed under reduced pressure to afford **12** as a white hygroscopic foam (14.5 mg, quant.). Ʋ_max_ (ATR): 2757 – 3257 (N^+^-H), 1748 (C=O), 1649 (Aryl C=C), 1598 (Aryl C=C) cm^−1^.^1^H NMR (400 MHz, D_2_O): *δ*_H_ 7.87 (1H, d, *J* = 9.5 Hz, H-4), 7.47 (1H, d, *J* = 8.5 Hz, H-5), 7.31 (1H, d, *J* = 2.0 Hz, H-8), 7.20 (1H, dd, *J* = 8.5, 2.1 Hz, H-6), 6.30 (1H, d, *J* = 9.4 Hz, H-3), 3.69 (2H, t, *J* = 5.9 Hz, NH_3_C*H*_*2*_), 3.24 (2H, t, *J* = 6.0 Hz, CH_3_NHC*H*_*2*_), 3.09 (3H, s, CH_3_). ^13^C NMR (101 MHz, D_2_O): *δ*_C_ 164.39 (C), 162.75 (q, *J* = 35.7 Hz, C), 157.33 (C), 153.67 (C), 145.57 (CH), 142.48 (C), 128.61 (CH), 117.24 (CH), 116.22 (q, *J* = 291.3 Hz, C), 114.35 (C), 112.55 (CH), 107.06 (CH), 46.08 (CH_2_), 37.82 (CH_2_), 34.65 (CH_3_). ^19^F NMR (377 MHz, D_2_O): *δ*_F_ −75.63 (3F, s, TFA). HRMS (ESI^+^): C_13_H_16_N_3_O_3_ requires 262.1186 found 262.1191 (M + H)^+^. Assignment of ^1^H and ^13^C NMR signals was supported by analysis of COSY, HSQC and DEPT experiments.

#### 4.1.10 3‐Methyl‐3‐[2‐(methylazaniumyl)ethyl]‐1‐(2‐oxo‐2H‐chromen‐7‐yl)urea trifluoroacetate (ACoum2) 13

Trifluoroacetic acid (2 mL) was added to a solution of **30** (75.0 mg, 0.200 mmol, 1.00 eq.) in anhydrous dichloromethane (2 mL) and the reaction was stirred at room temperature for 1 h under argon. The solvent was removed under reduced pressure to afford **13** as a white hygroscopic foam (74.0 mg, quant.). Ʋ_max_ (ATR): 3138 (N^+^-H), 3067 (N^+^-H), 1682 (C=O), 1608 (Aryl C=C) cm^−1^. ^1^H NMR (400 MHz, D_2_O): *δ*_H_ 7.85 (1H, d, *J* = 9.5 Hz, H-4), 7.45 (1H, d, *J* = 8.5 Hz, H-5), 7.30 (1H, d, *J* = 2.0 Hz, H-8), 7.18 (1H, dd, *J* = 8.5, 2.1 Hz, H-6), 6.29 (1H, d, *J* = 9.5 Hz, H-3), 3.70 (2H, t, *J* = 5.7 Hz, CH_3_NH_2_C*H*_*2*_), 3.28 [2H, t, *J* = 5.7 Hz, CON(CH_3_)C*H*_*2*_], 3.09 (3H, s, NH_2_C*H*_*3*_), 2.76 (3H, s, CONCH_3_). ^13^C NMR (101 MHz, D_2_O): *δ*_C_ 164.50 (C), 162.81 (q, *J* = 35.7 Hz, C), 157.55 (C), 153.78 (C), 145.64 (CH), 142.44 (C), 128.69 (CH), 117.57 (CH), 116.24 (q, *J* = 291.5 Hz, C), 114.58 (CH), 112.76 (CH), 107.47 (CH), 47.45 (CH_2_), 45.13 (CH_2_), 34.58 (CH_3_), 33.07 (CH_3_). ^19^F NMR (377 MHz, D_2_O): *δ*_F_ −75.63 (3F, s, TFA). HRMS (ESI^+^): C_14_H_18_N_3_O_3_ requires 276.1343 found 276.1345 (M + H)^+^. Assignment of ^1^H and ^13^C NMR signals was supported by analysis of COSY, HSQC and DEPT experiments.

#### 4.1.11 4‐Hydroxy‐3‐methylnaphthalen‐1‐yl *N*‐methyl‐*N*‐[2-(methylazaniumyl)ethyl] carbamate chloride (MenOH) 15

An aqueous solution of HCl (0.3 mL, 6 M) was added to a solution of **31** (25.0 mg, 0.0581 mmol) in methanol (0.3 mL) and the reaction was stirred at reflux for 24 h under argon. The reaction was allowed to cool to room temperature and the solvent was removed under reduced pressure to afford **15** as a white hygroscopic foam (18.5 mg, quant.). NMR spectroscopy indicated a 2.3:1 mixture of diastereomers (rotamers) A:B. Ʋ_max_ (ATR): 1696 (C=O) cm^−1^. ^1^H NMR (400 MHz, CD_3_OD): *δ*_H_ 8.26 – 8.16 (1H, m, H-8^A and B^), 7.84 – 7.71 (1H, m, H-5^A and B^), 7.50 – 7.42 (2H, m, H-6^A and B^ and H-7^A and B^), 7.11 (1H, s, H-2^A and B^), 3.98 (2H, t, *J* = 6.0 Hz, CH_2_-1′^B^), 3.73 (2H, t, *J* = 5.8 Hz, CH_2_-1′^A^), 3.41 (1H, t, *J* = 5.5 Hz, CH_*2*_-2′^B^), 3.32 – 3.28 (5H, m, CH_*2*_-1′^A^ and O_2_CNCH_3_^A^), 3.10 (3H, s, O_2_CNCH_3_^B^), 2.82 (3H, s, NH_2_C*H*_*3*_^B^), 2.74 (3H, s, NH_2_C*H*_*3*_^A^) 2.40 (3H, s, PhC*H*_*3*_^A and B^). ^13^C NMR (101 MHz, CD_3_OD): *δ*_C_ 158.11 (C^A and B^), 148.97 (C^A and B^), 140.80 (C^A and B^), 127.81 (C^A and B^), 127.55 (C^A and B^), 126.60 (CH^A and B^), 126.36 (CH^A and B^), 123.27 (CH^B^), 123.18 (CH^A^), 122.78 (CH^B^), 122.61 (CH^A^), 121.91 (CH^A^), 121.83 (CH^B^), 118.98 (C^A and B^), 48.64∗ (CH_2_^A^), 48.45∗ (CH_2_^B^), 47.24 (CH_2_^A^), 47.00 (CH_2_^B^), 35.59 (CH_3_^A and B^), 34.12 (CH_3_^B^), 34.01 (CH_3_^A^), 16.43 (CH_3_^A and B^). HRMS (ESI^+^): C_16_H_21_N_2_O_3_ requires 289.1547 found 289.1556 (M^+^). Assignment of ^1^H and ^13^C NMR signals was supported by analysis of COSY, HSQC and DEPT experiments. ∗Signals were hidden behind the solvent signal in the ^13^C NMR spectrum but were observed in the DEPT spectrum.

#### 4.1.12 4‐Acetoxy‐3‐methylnaphthalen‐1‐yl *N*‐methyl‐*N‐*[2’-(methylazaniumyl)ethyl] carbamate trifluoroacetate (MenOAc) 16

Trifluoroacetic acid (2 mL) was added to a solution of **31** (6.5 mg, 0.152 mmol, 1.00 eq.) in anhydrous dichloromethane (2 mL) and the reaction was stirred at room temperature for 1 h under argon. The solvent was removed under reduced pressure to afford **16** as a white hygroscopic foam (6.5 mg, quant.). NMR spectroscopy indicated a 2.5:1 mixture of diastereomers (rotamers) A:B. ^1^H NMR (400 MHz, CD_3_OD): *δ*_H_ 7.99 – 7.86 (1H, m, H-8^A and B^), 7.84 (1H, s, H-5^A^), 7.82 (1H, s, H–5^B^), 7.63 – 7.48 (2H, m, H-6^A and B^ and H-7^A and B^), 7.28 (1H, s, H-2^A^), 7.26 (1H, s, H–2^B^), 3.99 (2H, t, *J* = 5.7 Hz, CH_2_-1′^B^), 3.75 (2H, t, *J* = 5.7 Hz, CH_2_-1′^A^), 3.43 (2H, t, *J* = 5.6 Hz, CH_2_-2′^B^), 3.36 – 3.27 (5H, m, CH_2_-2′^A^ and O_2_CNCH_3_^A^), 3.11 (3H, s, O_2_CNCH_3_^B^), 2.83 (3H, s, NH_2_C*H*_*3*_^B^)), 2.75 (3H, s, NH_2_C*H*_*3*_^A^), 2.49 (3H, s, O_2_CCH_3_^A and B^), 2.32 (3H, s, PhC*H*_*3*_^A and B^). HRMS (ESI^+^): C_18_H_23_N_2_O_4_ requires 331.1652 found 331.1657 (M + H)^+^.

#### 4.1.13 2-Methyl-1,4-naphthohydroquinone monoacetate 18

Following the patent procedure of Sunny Pharmtech [36], anhydrous diisopropylamine (0.273 mL, 1.94 mmol, 1.00 eq.) was added to a suspension of **26** (500 mg, 1.94 mmol, 1.00 eq.) in methanol (2.5 mL) and water (0.3 mL). The reaction was stirred at room temperature for 3 h under argon. The reaction was diluted with ethyl acetate (30 mL) and washed with an aqueous solution of HCl (30 mL, 1 M). The aqueous layer was extracted with ethyl acetate (20 mL). The organic layers were combined and washed with brine (50 mL). The organic layer was separated again, dried with MgSO_4_ and the solvent was removed under reduced pressure. The crude material was purified by column chromatography [SiO_2_, hexane: ethyl acetate 5–40%] to afford **18** as a brown solid (392 mg, 94%). Mp: 118 – 120 °C. ^1^H NMR (400 MHz, CDCl_3_): *δ*_H_ 8.02 (1H, d, *J* = 8.0 Hz, H-8), 7.66 (1H, d, *J* = 8.3 Hz, H-5), 7.54 – 7.37 (2H, m, H-7 and H-6), 6.49 (1H, s, H-3), 5.44 (1H, bs, OH), 2.48 (3H, s, O_2_CCH_3_), 2.22 (3H, s, PhC*H*_*3*_). ^13^C NMR (101 MHz, CDCl_3_): *δ*_C_ 170.17 (C), 149.39 (C), 137.86 (C), 127.82 (C), 127.19 (CH), 126.57 (C), 124.88 (CH), 123.90 (C), 122.19 (CH), 120.63 (CH), 111.11 (CH), 20.80 (CH_3_), 16.52 (CH_3_). Mass spectrometry by ESI ^±^ failed to identify the product. The ^1^H NMR data is in agreement with that previously reported [36].

#### 4.1.14 (4‐{[5’‐(Benzylsulfanyl)‐2′,4′‐dinitrophenyl]amino}butyl)triphenylphosphonium hexafluorophosphate 19

Benzyl mercaptan (349 μL, 2.98 mmol, 2.00 eq.) and anhydrous *N,N*-diisopropylethylamine (519 μL, 2.98 mmol, 2.00 eq.) were added to a solution of MitoCDNB **1** as the hexafluorophosphate salt in anhydrous acetonitrile (30 mL) and the reaction was stirred at reflux for 24 h under argon. The reaction was allowed to cool to room temperature and the solvent was removed under reduced pressure. The solid residue was re-dissolved in dichloromethane (30 mL), washed with a saturated solution of NH_4_Cl (25 mL) and the aqueous layer was extracted with dichloromethane (2 × 30 mL). The organic layers were combined, dried with MgSO_4_ and the solvent was removed under reduced pressure. The crude material was purified by column chromatography [SiO_2_, dichloromethane:methanol 0–20%] to afford **19** as an orange hygroscopic foam (806 mg, 70%). Ʋ_max_ (ATR): 1605 (Aryl C=C), 1576 (NO_2_) cm^−1^. ^1^H NMR (400 MHz, CD_3_CN): *δ*_H_ 9.03 (1H, s, H-3′), 8.30 (1H, m, NH), 7.85 (3H, m, Ph), 7.71 – 7.62 (12H, m, Ph), 7.50 – 7.43 (2H, m, Ph), 7.38 – 7.29 (2H, m, Ph), 7.29 – 7.23 (1H, m, Ph), 6.58 (1H, s, H-6’), 4.30 (2H, s, SCH_2_), 3.33 (2H, q, *J* = 6.4 Hz, CH_2_-4), 3.22 – 3.10 (2H, m, CH_2_-1), 1.78 (2H, qn, *J* = 6.8 Hz, CH_2_-2), 1.66 (2H, qn, *J* = 7.9 Hz, CH_2_-3). ^13^C NMR (101 MHz, CD_3_CN): *δ*_C_ 147.62 (C), 147.34 (C), 136.20 (d, *J* = 3.0 Hz, 3 x CH), 135.95 (C), 134.61 (d, *J* = 10.0 Hz, 6 x CH), 134.26 (C), 131.29 (d, *J* = 12.6 Hz, 6 x CH), 130.01 (CH), 129.96 (d, *J* = 9.8 Hz, CH), 128.87 (CH), 127.54 (CH), 119.05 (d, *J* = 86.5 Hz), 110.10 (CH), 42.69 (CH_2_), 37.90 (CH_2_), 29.65 (d, *J* = 16.6 Hz, CH_2_), 22.34 (d, *J* = 52.2 Hz, CH_2_), 20.46 (d, *J* = 3.9 Hz, CH_2_). ^31^P NMR (162 MHz, CD_3_CN): *δ*_P_ 23.69 (1P, s, PPh_3_), −144.62 (1P, sept, *J* = 705.8, PF_6_). HRMS (ESI^+^): C_35_H_33_N_3_O_4_PS requires 622.1940 found 622.1937 (M^+^). Assignment of ^1^H and ^13^C NMR signals was supported by analysis of COSY, HSQC and DEPT experiments.

#### 4.1.15 (4‐{[5’‐(Chlorosulfonyl)‐2′,4′‐dinitrophenyl]amino}butyl)triphenylphosphonium hexafluorophosphate 20

Adapting the procedure of Xin et al. [3,51]-dichloro-5,5-dimethylhydantoin (142 mg, 0.719 mmol, 2.00 eq.) was added to a solution of **19** (276 mg, 0.359 mmol, 1.00 eq.) in a solvent mixture of acetonitrile: acetic acid: water (2 mL: 0.1 mL: 0.1 mL) and the reaction was stirred at room temperature for 24 h under argon. The solvent was removed under reduced pressure and the residue was redissolved in dichloromethane (30 mL). The organic layer was washed with water (30 mL) and the aqueous layer was extracted with dichloromethane (2 × 20 mL). The organic layers were combined, dried with MgSO_4_ and the solvent was removed under reduced pressure. The crude material was purified by column chromatography [SiO_2_, dichloromethane:methanol 0–20%] to afford **20** as an orange hygroscopic foam (209 mg, 78%). Ʋ_max_ (ATR): 1615 (Aryl C=C), 1541 (NO_2_) cm^−1^. ^1^H NMR (400 MHz, CD_3_CN): *δ*_H_ 8.89 (1H, s, H-3′), 8.61 (1H, m, NH), 7.87 (3H, m, Ph), 7.77 – 7.62 (13H, m, H-6’ and Ph), 3.57 (2H, q, *J* = 6.6 Hz, CH_2_-4), 3.27 – 3.15 (2H, m, CH_2_-1), 1.92 – 1.85 (2H, m, CH_2_-2), 1.80 – 1.68 (2H, m, CH_2_-3). ^13^C NMR (101 MHz, CD_3_CN): *δ*_C_ 147.90 (C), 142.38 (C), 136.22 (d, *J* = 3.0 Hz, 3 x CH), 134.66 (d, *J* = 10.0 Hz, 6 x CH), 131.31 (d, *J* = 12.6 Hz, 6 x CH), 128.43 (CH), 119.68 (C), 119.09 (d, *J* = 86.5 Hz, C), 43.26 (CH_2_), 29.86 (d, *J* = 17.0 Hz, CH_2_), 22.41 (d, *J* = 52.1 Hz, CH_2_), 20.45 (d, *J* = 4.0 Hz, CH_2_). ^31^P NMR (162 MHz, CD_3_CN): *δ*_P_ 23.61 (1P, s, PPh_3_), −144.63 (1P, sept, *J* = 704.2 Hz, PF_6_). HRMS (ESI^+^): C_28_H_26_ClN_3_O_6_PS requires 598.0963 found 598.0850 (M^+^). Assignment of ^1^H and ^13^C NMR signals was supported by analysis of COSY, HSQC and DEPT experiments. Two signals corresponding to C were not identified in the ^13^C spectrum, perhaps due to coincidence with another signal.

#### 4.1.16 [4‐({5’‐[(2’’‐{[*N*-Boc](methyl)amino}ethyl)sulfamoyl]‐2,4-dinitrophenyl}amino)butyl]triphenylphosphonium trifluoroacetate 21

A solution of *N*-boc-*N*-methylethylenediamine (11.7 mg, 0.0672 mmol, 1.00 eq.) and triethylamine (18.8 μL, 0.134 mmol, 2.00 eq.) in anhydrous dichloromethane (0.4 mL) was added to a solution of **20** (50.0 mg, 0.0672 mmol, 1.00 eq.) in anhydrous dichloromethane (0.4 mL) and the reaction was stirred at room temperature for 3 h under argon. The reaction was diluted with dichloromethane (10 mL) and washed with an aqueous solution of HCl (10 mL, 1 M). The aqueous layer was extracted with dichloromethane (3 × 10 mL). The organic layers were combined, dried with MgSO_4_ and the solvent was removed under reduced pressure. The crude material was purified by column chromatography [SiO_2_, dichloromethane:methanol 0-20%], followed by semi-preparative RP-HPLC [0.1% TFA/water: acetonitrile, 30-70%] to obtain **21** as the trifluoroacetate salt as a yellow hygroscopic foam (18.9 mg, 33%). Ʋ_max_ (ATR): 1683 (C=O), 1614 (Aryl C=C) cm^−1^. ^1^H NMR (400 MHz, CDCl_3_): *δ*_H_ 8.87 (1H, s, H-3′), 8.58 (1H, t, *J* = 5.5 Hz, NH), 7.82 (3H, m, Ph), 7.75 – 7.59 (12H, m, Ph), 7.57 (1H, s, H-6′), 3.65 – 3.55 (2H, m, CH_2_-4), 3.45 – 3.33 (4H, m, CH_2_-1 and CH_2_-2″), 3.27 (2H, q, *J* = 5.5 Hz, CH_2_-1″), 2.83 (3H, s, NHC*H*_*3*_), 2.09 – 2.01 (2H, m, CH_2_-2), 1.88 – 1.76 (2H, m, CH_2_-3), 1.42 (9H, s, 3 x CH_3_). ^13^C NMR (101 MHz, CDCl_3_): *δ*_C_ 160.19 (C), 146.94 (C), 140.97 (C), 135.57 (d, *J* = 3.0 Hz, 3 x CH), 134.13 (C), 133.47 (d, *J* = 10.0 Hz, 6 x CH), 130.78 (d, *J* = 12.6 Hz, 6 x CH), 130.57 (C), 127.21 (CH), 117.74 (d, *J* = 86.3 Hz, 3 x C), 117.59 (CH), 48.21 (CH_2_) 42.36 (2 x CH_2_), 35.19 (CH_3_), 29.20 (d, *J* = 17.0 Hz, CH_2_), 28.43 (3 x CH_3_), 22.12 (d, *J* = 52.4 Hz, CH_2_), 19.81 (d, *J* = 3.8 Hz, CH_2_). ^31^P NMR (162 MHz, CDCl_3_): *δ*_P_ 23.72 (1P, s, PPh_3_). ^19^F NMR (377 MHz, CDCl_3_): *δ*_F_ −75.76 (3F, s, TFA). HRMS (ESI^+^): C_36_H_43_N_5_O_8_PS requires 736.2564 found 736.2589 (M^+^). Assignment of ^1^H and ^13^C NMR signals was supported by analysis of COSY, HSQC and DEPT experiments. The signal corresponding to *C*(*t*-Bu) was not identified in the ^13^C NMR spectrum, perhaps due to coincidence with the solvent signal. The signals corresponding to the TFA^−^ anion were not observed in the ^13^C NMR spectrum.

#### 4.1.17 [4‐({5’‐[(2’’‐{[(*tert*‐butoxy)carbonyl](methyl)amino}ethyl)(methyl)sulfamoyl]‐2′,4′‐dinitrophenyl}amino)butyl]triphenylphosphonium hexafluorophosphate 22

*Tert*-butyl methyl(2-(methylamino)ethyl)carbamate (97.7 mg, 0.519 mmol, 0.900 eq.) and triethylamine (162 μL, 1.15 mmol, 2.00 eq.) were added to a solution of **20** (429 mg, 0.576 mmol, 1.00 eq) in anhydrous dichloromethane (5.8 mL) and the reaction was stirred at room temperature for 3 h under argon. The reaction was diluted with dichloromethane (15 mL) and washed with an aqueous solution of HCl (15 mL, 1 M). The aqueous layer was extracted with dichloromethane (3 × 15 mL). The organic layers were combined, dried with MgSO_4_ and the solvent was removed under reduced pressure. The crude material was purified by column chromatography [SiO_2_, dichloromethane:methanol 0-20%] to afford **22** as an orange hygroscopic foam (303 mg, 59%). NMR spectroscopy indicated a 1:1 mixture of diastereomers (rotamers) A:B. Ʋ_max_ (ATR): 1685 (Aryl C=C), 1616 (Aryl C=C) cm^−1^. ^1^H NMR (400 MHz, CDCl_3_): *δ*_H_ 8.61 (1H, s, H-3′^A and B^), 8.40 (1H, t, *J* = 5.3 Hz, NH^A and B^), 7.84 – 7.59 (15H, m, Ph^A and B^), 7.37 (1H, s, H-6′^A or B^), 7.35 (1H, s, H-6′^A or B^), 3.52 (2H, q, *J* = 6.2 Hz, CH_2_-4^A and B^), 3.49 – 3.37 (4H, m, CH_2_-1″^A and B^ and CH_2_-2″^A and B^), 3.31 – 3.20 (2H, m, CH_2_-1^A and B^), 2.98 (3H, s, O_2_SNCH_3_^A and B^ or BocNC*H*_*3*_^A and B^), 2.84 (3H, s, O_2_SNCH_3_^A and B^ or BocNC*H*_*3*_^A and B^), 2.05 (2H, qn, *J* = 7.7 Hz, CH_2_-2^A and B^), 1.90 – 1.74 (2H, m, CH_2_-3^A and B^), 1.43 (9H, s, 3 x CH_3_^A or B^), 1.41 (9H, s, 3 x CH_3_^A or B^). ^13^C NMR (101 MHz, CDCl_3_): *δ*_C_ 155.99 (C^A or B^), 155.61 (C^A or B^), 146.49 (C^A and B^), 140.50 (C^A or B^), 140.40 (C^A or B^), 135.48 (d, *J* = 3.0 Hz, 3 x CH^A and B^), 134.72 (C^A and B^), 133.49 (d, *J* = 9.9 Hz, 6 x CH^A and B^), 130.79 (d, *J* = 12.6 Hz, 6 x CH^A and B^), 130.33 (C^A and B^), 126.04 (CH^A and B^), 117.76 (CH^A^ ^or B^), 117.71 (d, *J* = 86.0 Hz, 3 x C^A and B^), 117.62 (CH^A or B^), 79.98 (C^A or B^), 79.65 (C^A or B^), 48.62 (CH_2_^A or B^), 48.10 (CH_2_^A or B^), 47.08 (CH_2_^A or B^), 46.30 (CH_2_^A or B^), 42.46 (CH_2_^A and B^), 35.39 (CH_3_^A or^ ^B^), 35.24 (CH_3_^A or B^), 34.89 (CH_3_^A or B^), 34.49 (CH_3_^A or B^), 29.25 (d, *J* = 16.8 Hz, CH_2_^A and B^), 28.49 (3 x CH_3_^A and B^), 22.07 (d, *J* = 52.6 Hz, CH_2_^A and B^), 19.92 (d, *J* = 3.5 Hz, CH_2_^A and B^). ^31^P NMR (162 MHz, CDCl_3_): *δ*_P_ 23.63 (1P, s, PPh_3_), −144.38 (sept, *J* = 713.2 Hz, PF_6_). HRMS (ESI^+^): C_37_H_45_N_5_O_8_PS requires 750.2721 found 750.2734 (M^+^). Assignment of ^1^H and ^13^C NMR signals was supported by analysis of COSY, HSQC and DEPT experiments.

#### 4.1.18 {4‐[(5’‐{[2’’‐(Methylamino)ethyl]sulfamoyl}‐2′,4′‐dinitrophenyl)amino]butyl}triphenylphosphonium bistrifluoroacetate 23

Trifluoroacetic acid (0.9 mL) was added to a solution of **21** (100 mg, 0.120 mmol, 1.00 eq.) in anhydrous dichloromethane (0.9 mL) and the reaction was stirred at room temperature for 1 h under argon. The solvent was removed under reduced pressure and the crude material purified by column chromatography [SiO_2_, dichloromethane:methanol 0-30%] to afford **23** as a yellow hygroscopic foam (79.8 mg, 91%). Ʋ_max_ (ATR): 1671 (Aryl C=C), 1616 (Aryl C=C) cm^−1^. ^1^H NMR (400 MHz, CD_3_CN): *δ*_H_ 9.52 (2H, br s, CH_3_N*H*_*2*_), 8.75 (1H, s, H-3′), 8.49 (2H, t, *J* = 5.9 Hz, PhN*H*), 8.27 (1H, br s, O_2_SN*H*), 7.89 – 7.79 (3H, m, Ph), 7.75 – 7.64 (12H, m, Ph), 7.58 (1H, s, H-6′), 3.60 (2H, q, *J* = 6.5 Hz, CH_2_-4), 3.46 (2H, t, *J* = 5.6 Hz, CH_2_-1″), 3.34 – 3.22 (2H, m, CH_2_-1), 3.14 (2H, t, *J* = 5.6 Hz, CH_2_-2″), 2.61 (3H, s, CH_3_), 1.90 (2H, m, CH_2_-2), 1.82 – 1.67 (2H, m, CH_2_-3). ^13^C NMR (101 MHz, CD_3_CN): *δ*_C_ 148.02 (C), 141.23 (C), 136.12 (d, *J* = 3.0 Hz, 3 x CH), 135.08 (C), 134.64 (d, *J* = 10.1 Hz, 6 x CH), 131.57 (C), 131.25 (d, *J* = 12.6 Hz, 6 x CH), 127.48 (CH), 119.16 (d, *J* = 86.5 Hz, 3 x C), 50.10 (CH_2_), 42.89 (CH_2_), 40.80 (CH_2_), 33.73 (CH_3_), 29.92 (d, *J* = 16.9 Hz, CH_2_), 22.25 (d, *J* = 52.0 Hz, CH_2_), 20.33 (d, *J* = 3.8 Hz, CH_2_). ^31^P NMR (162 MHz, CD_3_CN): *δ*_P_ 23.77 (1P, s, PPh_3_). ^19^F NMR (377 MHz, CD_3_CN): *δ*_F_ −75.85 (6F, s, 2 x TFA). HRMS (ESI^+^): C_31_H_35_N_5_O_6_PS requires 636.2040 found 636.2066 (M^+^). Assignment of ^1^H and ^13^C NMR signals was supported by analysis of COSY, HSQC and DEPT experiments. The signals corresponding to the TFA^−^ anions were not observed in the ^13^C NMR spectrum.

#### 4.1.19 {4‐[(5’‐{Methyl[2’’‐(methylazaniumyl)ethyl]sulfamoyl}‐2′,4′‐dinitrophenyl)amino]butyl}triphenylphosphonium trifluoroacetate hexafluorophosphate 24

Trifluoroacetic acid (1.7 mL) was added to a solution of **22** (303 mg, 0.338 mmol, 1.00 eq.) in anhydrous dichloromethane (1.7 mL) and the reaction was stirred at room temperature for 1 h under argon. The solvent was removed under reduced pressure to afford **24** as a yellow hygroscopic foam (286 mg, quant.). Ʋ_max_ (ATR): 1616 (Aryl C=C) cm^−1^. ^1^H NMR (400 MHz, CD_3_CN): *δ*_H_ 8.70 (1H, s, H-3′), 8.43 (1H, t, *J* = 5.9 Hz, PhN*H*), 7.92 – 7.82 (3H, m, Ph), 7.73 – 7.63 (12H, m, Ph), 7.55 (2H, s, CH_3_N*H*_*2*_), 7.29 (1H, s, H-6′), 3.61 (2H, t, *J* = 5.7 Hz, CH_2_-1″), 3.55 (2H, q, *J* = 6.3 Hz, CH_2_-2″), 3.27 – 3.15 (4H, m, CH_2_-1 and CH_2_-4), 3.03 (2H, s, O_2_SNCH_3_), 2.70 (3H, t, *J* = 5.4 Hz, NH_2_C*H*_*3*_), 1.92 – 1.85 (2H, m, CH_2_-2), 1.75 (2H, qn, *J* = 8.1 Hz, CH_2_-3). ^13^C NMR (101 MHz, CD_3_CN): *δ*_C_ 160.07 (q, *J* = 37.6 Hz, C), 147.51 (C), 139.50 (C), 136.19 (d, *J* = 3.0 Hz, 3 x CH), 135.44 (C), 134.64 (d, *J* = 10.0 Hz, 6 x CH), 131.94 (C), 131.28 (d, *J* = 12.6 Hz, 6 x CH), 127.07 (CH), 119.09 (d, *J* = 86.5 Hz, 3 x C), 118.20 (CH), 47.48 (CH_2_), 47.43 (CH_2_), 42.90 (CH_2_), 36.31 (CH_3_), 34.33 (CH_3_), 29.84 (d, *J* = 17.0 Hz, CH_2_), 22.35 (d, *J* = 52.2 Hz, CH_2_), 20.41 (d, *J* = 3.8 Hz, CH_2_). ^31^P NMR (162 MHz, CDCl_3_): *δ*_P_ 23.65 (1P, s, PPh_3_), −144.62 (1P, sept, *J* = 707.6 Hz, PF_6_). ^19^F NMR (377 MHz, CD_3_CN): *δ*_F_ −72.88 (6F, d, *J* = 707.6 Hz, PF_6_), −76.67 (3F, s TFA). HRMS (ESI^+/−^): C_32_H_38_N_5_O_6_PS requires 651.2269 found 586.2587 corresponding to the aniline C_32_H_37_N_5_O_4_P likely due to fragmentation during ionisation. Assignment of ^1^H and ^13^C NMR signals was supported by analysis of COSY, HSQC and DEPT experiments. The signal corresponding to CF_3_*C*OO^−^ of the TFA^−^ anion was not observed in the ^13^C NMR spectrum.

#### 4.1.20 2-Methyl-1,4-naphthohydroquinone diacetate 26

Following the patent procedure by Sunny Pharmtech [36], Ac_2_O (2.75 mL, 29.1 mmol, 2.50 eq.), DMAP (142 mg, 1.16 mmol, 0.100 mmol) and Pd/C (62.0 mg, 0.580 mmol, 0.0500 eq.) were added to a solution of menadione **25** (2.00 g, 11.6 mmol, 1.00 eq.) in ethyl acetate. The reaction was stirred at room temperature for 24 h under H_2_. The reaction was filtered through Celite under vacuum and washed with ethyl acetate (50 mL). The filtrate was washed with brine (2 × 50 mL). The organic layer was separated, dried with MgSO_4_ and the solvent removed under reduced pressure to afford **26** as a white solid (2.81 g, 94%). Mp: 108 – 110 °C. ^1^H NMR (400 MHz, CDCl_3_): *δ*_H_ 7.84 – 7.70 (2H, m, H-6 and H-7), 7.56 – 7.41 (2H, m, H-5 and H-8), 7.15 (1H, s, H-3), 2.48 (3H, s, O_2_CCH_3_), 2.46 (3H, s, O_2_CCH_3_), 2.33 (3H, s, PhC*H*_*3*_). ^13^C NMR (101 MHz, CDCl_3_): *δ*_C_ 169.57 (C), 169.04 (C), 144.26 (C), 142.28 (C), 128.05 (C), 127.24 (CH), 126.65 (C), 126.31 (C), 126.18 (CH), 121.61 (CH), 121.31 (CH), 120.87 (CH), 21.15 (CH_3_), 20.74 (CH_3_), 16.66 (CH_3_). HRMS (ESI^+^): C_15_H_18_NO_4_ requires 276.1230 found 276.1210 (M^+^). The ^1^H NMR data is in agreement with that previously reported [36].

#### 4.1.21 2‐Oxo‐2H‐chromen‐7‐yl N‐(2‐{[(*tert*‐butoxy)carbonyl]amino}ethyl)‐N-methylcarbamate 27

Triphosgene (30.2 mg, 0.102 mmol, 0.330 eq.) and anhydrous *N,N*-diisopropylethylamine (53.7 μL, 0.308 mmol, 1.00 eq.) were added to a solution of 7-hydroxycoumarin (50.0 mg, 0.308 mmol, 1.00 eq.) in an anhydrous solvent mixture of tetrahydrofuran: dichloromethane (1:1, 6 mL). The reaction was stirred at room temperature for 1 h under argon. A solution of *N*-boc-*N*-methylethylenediamine (64.5 mg, 0.370 mmol, 1.20 eq.) and anhydrous *N,N*-diisopropylethylamine (64.4 μL, 0.370 mmol, 1.20 eq.) were added to the reaction, which was stirred at room temperature for an additional 1 h under argon. The solvent was removed under reduced pressure and the solid residue was re-dissolved in dichloromethane (20 mL). The organic layer was washed with a saturated solution of NH_4_Cl (20 mL) and the aqueous layer was extracted with dichloromethane (2 × 15 mL). The organic layers were combined, dried with MgSO_4_ and the solvent was removed under reduced pressure. The crude material was purified by column chromatography [SiO_2_, hexane:ethyl acetate 10-80%] to afford **29** as a white hygroscopic foam (45.2 mg, 40%). NMR spectroscopy indicated a 1.2:1 mixture of diastereomers (rotamers) A:B. Ʋ_max_ (ATR): 3393 (N–H), 1726 (C=O), 1696 (C=O), 1617 (Aryl C=C) cm^−1^. ^1^H NMR (400 MHz, CDCl_3_): *δ*_H_ 7.68 (1H, d, *J* = 9.5 Hz, H-4^A and B^), 7.46 (1H, d, *J* = 8.5 Hz, H-5^A and B^), 7.21 – 7.11 (1H, m, H-8^A and B^), 7.10 (1H, m, H-6^A^), 7.08 (1H, m, H–6^B^) 6.37 (1H, d, *J* = 9.5 Hz, H-3^A and B^), 3.56 (2H, t, *J* = 6.0 Hz, CH_3_NC*H*_*2*_^B^), 3.48 (2H, t, *J* = 6.1 Hz, CH_3_NC*H*_*2*_^A^), 3.42 – 3.33 (2H, m, BocNHC*H*_*2*_^A and B^), 3.14 (3H, s, CH_3_^A^), 3.05 (3H, s, CH_3_^B^), 1.43 (9H, s, 3 x CH_3_^A and B^). ^13^C NMR (101 MHz, CDCl_3_): *δ*_C_ 160.55 (C^A^), 160.62 (C^B^), 156.15 (C^A or B^), 155.92 (C^A or B^), 154.81 (C^A and B^), 154.40 (C^B^), 154.16 (C^A^), 154.15 (C^A^), 153.90 (C^B^), 143.07 (CH^A and B^), 128.46 (CH^A and B^), 118.71 (CH^B^), 118.61 (CH^A^), 116.39 (C^B^), 116.31 (C^A^), 115.78 (CH^B^), 115.85 (CH^A^), 110.62 (CH^B^), 110.44 (CH^A^), 79.96 (C^B^), 79.68 (C^A^), 49.27 (CH_2_^A^), 49.13 (CH_2_^B^), 38.80 (CH_2_^B^), 38.64 (CH_2_^A^), 35.57 (CH_3_^A^), 35.50 (CH_3_^B^), 28.52 (3 x CH_3_^A^), 28.50 (3 x CH_3_^B^). HRMS (ESI^+^): C_18_H_22_N_2_O_6_Na requires 385.1370 found 385.1367 (M + Na)^+^. Assignment of ^1^H and ^13^C NMR signals was supported by analysis of COSY, HSQC and DEPT experiments.

#### 4.1.22 2‐Oxo‐2H‐chromen‐7‐yl N-methyl-N‐(2‐{[(*tert*‐butoxy)carbonyl]amino}ethyl)‐N-methylcarbamate 28

Triphosgene (61.0 mg, 0.206 mmol, 0.330 eq.) and anhydrous *N,N*-diisopropylethylamine (107.4 μL, 0.616 mmol, 1.00 eq.) were added to a solution of 7-hydroxycoumarin (100 mg, 0.616 mmol, 1.00 eq.) in anhydrous dichloromethane (6 mL). The reaction was stirred at room temperature for 1 h under argon. A solution of *tert*-butyl methyl(2-(methylamino)ethyl)carbamate (139 mg, 0.740 mmol, 1.20 eq.) and anhydrous *N,N*-diisopropylethylamine (129 μL, 0.740 mmol, 1.20 eq.) in anhydrous dichloromethane (6 mL) was added to the reaction, which was stirred at room temperature for an additional 1 h under argon. The solvent was removed under reduced pressure and the solid residue was re-dissolved in dichloromethane (20 mL). The organic layer was washed with a saturated solution of NH_4_Cl (20 mL), and the aqueous layer was extracted with dichloromethane (2 × 15 mL). The organic layers were combined, dried with MgSO_4_ and the solvent was removed under reduced pressure. The crude material was purified by column chromatography [SiO_2_, hexane:ethyl acetate 10-80%] to afford **28** as a white hygroscopic foam (115 mg, 50%). NMR spectroscopy indicated a mixture of four diastereomers A:B:C:D, but the ^1^H and ^13^C NMR spectra were too complex to differentiate between them and determine their ratio with confidence. Ʋ_max_ (ATR): 1724 (C=O), 1685 (C=O) cm^−1^. ^1^H NMR (400 MHz, CO(CD_3_)_2_): *δ*_H_ 7.97 (1H, d, *J* = 9.6 Hz, H-4^A, B, C and D^), 7.68 (1H, d, *J* = 8.4 Hz, H-5^A, B, C and D^), 7.21 – 7.11 (2H, m, H-6^A, B, C and D^ and H-8^A, B, C and D^), 6.37 (1H, d, *J* = 9.6 Hz, H-3^A, B, C and D^), 3.72 – 3.39 [4H, m, BocNC*H*_*2*_^A, B, C and D^ and CO_2_N(CH_3_)C*H*_*2*_^A, B, C and D^], 3.20 – 2.99 (3H, m, BocNCH_3_^A, B, C and D^ or O_2_CNCH_3_^A, B, C and D^), 2.98 – 2.85 (3H, m, BocNCH_3_^A, B, C and D^ or O_2_CNCH_3_^A, B, C and D^), 1.50 – 1.40 (9H, m, 3 x CH_3_^A, B, C and D^). HRMS (ESI^+^): C_19_H_24_N_2_O_6_Na requires 399.1527 found 399.1530 (M + Na)^+^. Assignment of ^1^H NMR signals was supported by analysis of COSY, HSQC and DEPT experiments. The ^13^C NMR spectra was too complex to interpret with confidence.

#### 4.1.23 *Tert*‐butyl N‐(2‐{methyl[(2‐oxo‐2H‐chromen‐7‐yl)carbamoyl]amino}ethyl)carbamate 29

Anhydrous phosgene solution (15 wt % in toluene) (244 μL, 0.341 mmol, 1.10 eq.) and triethylamine (87.0 μL, 0.620 mmol, 2.00 eq.) were added to a solution of 7-aminocoumarin **14** (50.0 mg, 0.310 mmol, 1.00 eq.) in anhydrous dichloromethane (3.1 mL) at 0 °C. The reaction was stirred at 0 °C for 1 h under argon. The solvent was removed under reduced pressure. The solid residue was re-dissolved in anhydrous acetonitrile (3.1 mL) and triethylamine (87.0 μL, 0.620 mmol, 2.00 eq.) and *N*-boc-*N*-methylethylenediamine (54.0 mg, 0.341 mmol, 1.10 eq.) were added. The reaction was stirred at room temperature for 18 h under argon. The solvent was removed under reduced pressure and the solid residue re-dissolved in dichloromethane (20 mL). The organic layer was washed with an aqueous solution of HCl (20 mL, 1 M) and the aqueous layer was extracted with dichloromethane (2 × 15 mL). The organic layers were combined, dried with MgSO_4_ and the solvent was removed under reduced pressure. The crude material was purified by column chromatography [SiO_2_, dichloromethane:methanol 0-20%] to afford **29** as a white hygroscopic foam (86.1 mg, 77%). Ʋ_max_ (ATR): 1667 (C=O) cm^−1^. ^1^H NMR (400 MHz, CDCl_3_): *δ*_H_ 8.28 (1H, s, ArNH), 7.63 – 7.48 (3H, m, H-4, H-6 and H-8), 7.29 (1H, d, *J* = 8.5 Hz, H-5), 6.18 (1H, d, *J* = 9.4 Hz, H-3), 5.03 (1H, s, BocNH), 3.40 (2H, t, *J* = 7.2 Hz, CH_3_NC*H*_*2*_), 3.27 – 3.19 (2H, m, BocNHC*H*_*2*_), 2.98 (3H, s, NCH_3_), 1.40 (9H, s, 3 x CH_3_). ^13^C NMR (101 MHz, CDCl_3_): *δ*_C_ 161.80 (C), 157.32 (C), 155.28 (C), 155.14 (C), 144.32 (C), 143.62 (CH), 128.19 (CH), 115.84 (CH), 113.66 (C), 113.58 (CH), 106.39 (CH), 80.71 (C), 48.13 (CH_2_), 39.67 (CH_2_), 35.35 (CH_3_), 28.53 (3 x CH_3_). HRMS (ESI^+^): C_18_H_23_N_3_O_5_Na requires 384.1530 found 384.1532 (M + Na)^+^. Assignment of ^1^H and ^13^C NMR signals was supported by analysis of COSY, HSQC and DEPT experiments.

#### 4.1.24 *Tert*‐butyl N‐methyl‐N‐(2‐{methyl[(2‐oxo‐2H‐chromen‐7‐yl)carbamoyl]amino}ethyl) carbamate 30

Triphosgene (30.7 mg, 0.103 mmol, 0.330 eq.) and anhydrous *N,N*-diisopropylethylamine (81.0 μL, 0.465 mmol, 1.50 eq.) were added to a solution of 7-aminocoumarin **14** (50.0 mg, 0.310 mmol, 1.00 eq.) in anhydrous dichloromethane (3.1 mL). The reaction was stirred at room temperature for 1 h under argon. A solution of *tert*-butyl methyl(2-(methylamino)ethyl)carbamate (64.0 mg, 0.341 mmol, 1.10 eq.) and anhydrous *N,N*-diisopropylethylamine (81.0 μL, 0.465 mmol, 1.50 eq.) in anhydrous dichloromethane (3.1 mL) was added to the reaction, which was stirred at room temperature for an additional 3 h under argon. The reaction was diluted with dichloromethane (20 mL). The organic layer was washed with a saturated solution of NH_4_Cl (20 mL) and the aqueous layer was extracted with dichloromethane (2 × 15 mL). The organic layers were combined, dried with MgSO_4_ and the solvent was removed under reduced pressure. The crude material was purified by column chromatography [SiO_2_, hexane:ethyl acetate 10-100%] to afford **30** as a white hygroscopic foam (112 mg, 95%). Ʋ_max_ (ATR): 1697 (C=O), 1663 (Aryl C=C), 1620 (Aryl C=C) cm^−1^. ^1^H NMR (400 MHz, CDCl_3_): *δ*_H_ 7.68 (1H, br s, H-8), 7.65 – 7.56 (2H, m, H-4 and H-6), 7.33 (1H, d, *J* = 8.5 Hz, H-5), 6.22 (1H, d, *J* = 9.4 Hz, H-3), 3.49 – 3.41 (2H, m, BocNC*H*_*2*_ or CH_3_NC*H*_*2*_), 3.40 – 3.31 (2H, m, BocNC*H*_*2*_ or CH_3_NC*H*_*2*_), 3.04 (3H, s, OCNCH_3_ or BocNCH_3_), 2.93 (3H, s, OCNCH_3_ or BocNCH_3_), 1.48 (9H, s, 3 x CH_3_). ^13^C NMR (101 MHz, CDCl_3_): *δ*_C_ 161.76 (C), 156.89 (C), 155.15 (2 x C), 144.59 (C), 143.57 (CH), 128.11 (CH), 115.74 (CH), 113.50 (C), 113.45 (CH), 106.29 (CH), 80.96 (C), 48.25 (CH_2_), 46.60 (CH_2_), 36.22 (CH_3_), 35.51 (CH_3_), 28.54 (3 x CH_3_). HRMS (ESI^+^): C_19_H_26_N_3_O_5_ requires 376.1867 found 376.1863 (M + H)^+^. Assignment of ^1^H and ^13^C NMR signals was supported by analysis of COSY, HSQC and DEPT experiments.

#### 4.1.25 4‐{[(2’‐{[(*Tert*‐butoxy)carbonyl](methyl)amino}ethyl)(methyl)carbamoyl]oxy}‐2‐methylnaphthalen‐1‐yl acetate 31

Triphosgene (22.9 mg, 0.0770 mmol, 0.330 eq.) and anhydrous *N,N*-diisopropylethylamine (40.2 μL, 0.231 mmol, 1.00 eq.) were added to a solution of **18** (50.0 mg, 0.231 mmol, 1.00 eq.) in anhydrous dichloromethane (1.2 mL) and the reaction was stirred at room temperature for 1 h under argon. A solution of *tert*-butyl methyl(2-(methylamino)ethyl)carbamate (43.5 mg, 0.231 mmol, 1.00 eq.) and *N,N*-diisopropylethylamine (40.2 μL, 0.231 mmol, 1.00 eq.) in anhydrous dichloromethane (1.2 mL) was added to the reaction, which was stirred at room temperature for an additional 1 h under argon. The reaction was diluted with dichloromethane (20 mL). The organic layer was washed with a saturated solution of NH_4_Cl (20 mL), and the aqueous layer was extracted with dichloromethane (2 × 15 mL). The organic layers were combined, dried with MgSO_4_ and the solvent was removed under reduced pressure. The crude material was purified by column chromatography [SiO_2_, hexane:ethyl acetate 10-50%] to afford **31** as a colourless oil (70.1 mg, 70%). NMR spectroscopy indicated a 2:1:1 mixture of diastereomers (rotamers) A:B:C. Ʋ_max_ (ATR): 1719 (C=O), 1685 (C=O) cm^−1^. ^1^H NMR (400 MHz, CDCl_3_): *δ*_H_ 7.93 – 7.79 (1H, m, H-5^A, B and C^), 7.77 – 7.69 (1H, m, H-8^A, B and C^), 7.55 – 7.42 (2H, m, H-6^A, B and C^ and H-7^A, B and C^), 7.19 (1H, s, H–3^B and C^), 7.17 (1H, s, H-3^A^), 3.77 – 3.41 (4H, m, CH_2_-1′^A, B and C^ and CH_2_-2′^A, B and C^), 3.28 (3H, s, O_2_CNCH_3_^B and C^ or BocNCH_3_^B and C^), 3.10 (3H, s, O_2_CNCH_3_^A^ or BocNCH_3_^A^), 2.96 (3H, s, O_2_CNCH_3_^A^ or BocNCH_3_^A^), 2.95 – 2.88 (3H, m, O_2_CNCH_3_^B and C^ or BocNCH_3_^B and C^), 2.47 (3H, s, O_2_CCH_3_^A, B and C^), 2.32 (3H, s, PhC*H*_*3*_^A, B and C^), 1.56 – 1.43 (9H, m, 3 x CH_3_^A, B and C^). HRMS (ESI^+^): C_23_H_34_N_3_O_6_ requires 448.2442 found 448.2451 (M + NH_4_)^+^. Assignment of ^1^H and ^13^C NMR signals was supported by analysis of COSY, HSQC and DEPT experiments. The ^13^C NMR spectra was too complex to interpret.

### *4.2* *In vitro* kinetics by fluorescence

UV-Vis absorbance spectra, fluorescence spectra and fluorescence kinetics were measured for the coumarin-containing compounds: MitoHCoum1 **3**, MitoHCoum2 **4**, HCoum1 **7**, HCoum2 **8**, MitoACoum1 **10**, MitoACoum2 **11**, ACoum1 **12**, ACoum2 **13**, 7-hydroxycoumarin **9** and 7-aminocoumarin **14**. Measurements were performed at 30 °C using a Horiba Duetta spectrometer and a quartz cuvette with a pathlength of 1 cm. UV-Vis absorbance spectra were recorded for 100 μM compound in 0.1 M HEPES pH 8.0 buffer between 220 nm and 800 nm. Fluorescence emission spectra were recorded for 10 μM compound, using the wavelength of maximum absorbance as the excitation wavelength, and measuring between 350 nm and 750 nm. Calibration curves of concentration versus fluorescence were created for 7-hydroxycoumarin **9**, ACoum1 **12** and ACoum2 **13** at pH 8.0. For HCoum1 **7**, HCoum2 **8**, ACoum1 **12** and ACoum2 **13**, fluorescence assays were performed in triplicate on 10 μM compound at pH 8.0 and 30 °C. For MitoHCoum1 **3**, MitoHCoum2 **4**, MitoACoum1 **10** and MitoACoum2 **11**, assays were performed in triplicate under the same conditions but in the presence of excess GSH to ensure that the reactions observed pseudo-first order behaviour. For the reactions of MitoHCoum1 **3** and MitoHCoum2 **4**, the assays were performed using 10 mM GSH to emulate the concentration in the mitochondrial matrix. For the reactions of MitoACoum1 **10** and MitoACoum2 **11** with 10 mM GSH, the reaction rate was too fast to obtain an accurate initial rate, so 2 mM GSH was used instead. The initial rate of MitoHCoum1 **3** was measured between 120 and 300 s where the reaction was linear after an initial period of lag due to the GSH activation step. No lag was observed in the reaction of MitoHCoum2 **4**, allowing the initial rate to be measured between 0 and 180 s. The reaction was monitored by measuring the initial rate of formation of the appropriate coumarin by fluorescence. Fluorescence intensity was recorded every 10 s for at least 60 s, and the initial rate data (in arbitrary units per second, A.U. s^−1^) was converted to μM s^−1^ using the appropriate calibration curve. For each of the triplicate measurements, the initial rate data was plotted against time to produce a linear line. The first (or pseudo-first) order rate constant was determined by dividing the gradient of the line by the concentration of the compound. The second-order rate constant was calculated by dividing the pseudo-first order rate constant by the concentration of GSH. Errors in the rate constants were calculated as the standard deviation of three rate constants determined from triplicate measurements. Predicted graphs were generated on Microsoft Excel, using the first-order rate law or the consecutive reaction model (Equation (1)). To calculate a predicted value for *k*_*cyclisation*_ in the reaction of MitoHCoum2 **4**, the observed data and the value for *k*_*SNAr*_ (obtained from the observed data for MitoACoum2 **11**) were fitted to the consecutive reaction model. The least-squares method and the Data Solver function were used to generate a non-linear line of best fit to the observed data.

### *4.3* *In vitro* kinetics by RP-HPLC

MitoCDNB **1**, MitoGSDNB **2**, MitoHCoum1 **3**, MitoHCoum2 **4**, MitoMenOH **5**, MitoMenOAc **6**, HCoum1 **7**, HCoum2 **8**, MitoACoum1 **10**, MitoACoum2 **11**, ACoum1 **12**, ACoum2 **13**, MenOH **15**, MenOAc **16**, monoacetate **18** were stored as 10 mM stocks in ethanol (EtOH) at −20 °C. 10 μM standards were prepared in 0.1 M HEPES pH 8.0 buffer acidified to pH 4.0 by addition of HCl. For the assays, 10 μM compound was incubated at pH 8.0 and 37 °C**,** and 100 μM GSH was added. At appropriate time-points, 1 mL aliquots were transferred from the reactions into fresh tubes on ice and quenched by acidification as above. The standards and samples were measured by RP-HPLC using a C18 column (Jupiter 300A, Phenomenex) attached to a Widepore C18 guard column (Phenomenex), all driven by a Gilson 321 pump. A flow rate of 1 mL/min was used with a gradient of 0.1 % (v/v) TFA in water (Buffer A) and 0.1 % (v/v) TFA in acetonitrile (MeCN) (Buffer B) at: (% B). 0-2 min; 5 %, 2-17 min; 5-100 %, 17-20 min; 100 %, 20-22 min; 100-5%, 22-26 min, 5%. Absorbance was measured at 220 or 254 nm using a UV-Visible detector (Gilson 151). Fluorescence was measured at λ_ex_ = 342 nm and λ_em_ = 454 nm using a fluorescence detector (Shimadzu RF-10A). All peak areas were calculated using the Trilution LC v3.0.26.0 software (Gilson) and divided by 10^6^ before processing in GraphPad Prism 10.

### 4.4 Mitochondrial isolation from tissue

For isolated mitochondrial studies, livers and hearts were prepared from rats. In both cases mitochondria were isolated by homogenisation using a dounce homogeniser followed by differential centrifugation at 4 °C. Liver mitochondria were prepared in STE buffer (250 mM sucrose, 10 mM Tris, 1 mM EGTA, pH 7.4). Heart mitochondria were isolated using STE buffer supplemented with 0.1 % (w/v) fatty acid free bovine serum albumin (BSA). Homogenates were pelleted by centrifugation at 1000×*g* for 3 min for liver or 700×*g* for 5 min for heart. For heart, the supernatant was collected by decanting through two layers of pre-wetted muslin. The remaining pellet was resuspended in STE buffer supplemented with BSA and centrifuged again. The supernatant was collected as before. The liver and heart supernatants were centrifuged at 10,000×*g* for 10 min. The mitochondrial pellets were resuspended in STE buffer for liver and STE buffer supplemented with BSA for heart, and re-centrifuged at 10,000×*g*. Finally, the pellets were resuspended in 100 μL STE per heart and 5 mL STE per liver. Protein concentration was measured using the bicinchoninic acid (BCA) assay with BCA as a standard.

### 4.5 Mitochondrial incubations

Prior to all biological experiments, the synthesised prodrug compounds, MitoHCoum2 **3 TFA** and MitoMenOAc **6 TFA**, were ion-exchanged to the chloride salt using Amberlite chloride ion-exchange resin. RLM or RHM (1 mg protein/mL) were incubated in KCl buffer (120 mM KCl, 10 mM HEPES, 1 mM EGTA, pH 7.2) with succinate (10 mM) and rotenone (4 mg/mL), or, glutamate (10 mM) and malate (10 mM) at 37 °C. For incubations analysed by RP-HPLC, the mitochondria were energised with glutamate and malate instead of succinate and rotenone because rotenone is UV-active and its signal was susceptible to overlapping with other signals in the chromatograms. FCCP (1 μM) was added where appropriate. 5 μM propyl TPP was added as a control where appropriate. 5 or 10 μM MitoHCoum2 **3**, MitoMenOH **4** or MitoMenOAc **6** were added. The reaction of MitoHCoum2 **3** was analysed in a 96-well plate by fluorescence (λ_ex_ = 342 nm and λ_em_ = 454) using the CLARIOstar Plus plate-reader (BMG Labtech). Fluorescence values were converted to concentration using a calibration curve created for 7-hydroxycoumarin. To measure the different compounds produced in the reactions of MitoHCoum2 **4**, MitoMenOH **5** and MitoMenOAc **6**, after incubations, mitochondria were pelleted by centrifugation (10,000×*g*, 4 °C, 5 min). 750 μL supernatant was collected and diluted with MeCN (containing 0.1% TFA) to a final ratio of 3:1 aqueous:organic solution. The pellet was vortexed in MeCN (containing 0.1 % TFA). The supernatant and extracted pellet were centrifuged (17,000×*g*, 4 °C, 10 min). The supernatant of the extracted pellet was collected and diluted with water (containing 0.1% TFA) to a final ratio of 3:1 aqueous:organic. The samples were filtered through a syringe-driven 0.22 mm polyvinylidene difluoride (PVDF) filter unit (Millex Millipore) and analysed by RP-HPLC (as described in Section 4.3).

### 4.6 Glutathione measurements

To determine the total glutathione pool (GSH + 2 x GSSG) in the incubations described in section 4.5, mitochondria were pelleted by centrifugation (10,000×*g*, 4 °C, 5 min). The supernatant was removed and the pelleted mitochondria were treated with 100 μL 5% (w/v) sulfosalicylic acid with vortexing and centrifuged (17,000×*g*, 4 °C, 10 min). The supernatants were analysed by the GSH recycling assay as described in the literature [52,53]. Data are expressed as glutathione equivalents (GSH + 2 x GSSG) normalised to protein determined by the BCA assay.

### 4.7 Superoxide production by mitochondrial membranes

O_2_^•-^ generation by menadione **25** was measured using the acetylated cytochrome *c* (cyt *c*_acet_) reduction assay as described in the literature [54]. Bovine heart mitochondrial membranes (BHM) were kindly provided by the group of Professor Judy Hirst (Mitochondrial Biology Unit, University of Cambridge). Potassium phosphate (KPi) buffer (50 mM KPi-KOH, 1 mM EGTA, 100 μM diethylenetriaminepentaacetic acid (pH 7.8)) was supplemented with 100 μg protein/ml BHM, 50 μM cyt *c*_acet_, 400 nM myxothiazol and 2 mM KCN. The reaction mixture was supplemented with 1 mM NADH, or 5 mM succinate and 20 μM rotenone. Rotenone was used to inhibit the Q-binding site of complex I. 50 μM menadione **25** was added to initiate the reaction. Absorbance was measured at 550 nm in a SpectraMax Plus 384 plate reader (Molecular Devices) for 10 min at 37 °C. The reaction with NADH was repeated in the presence of 20 μM rotenone or 100 units/mL superoxide dismutate (SOD) from bovine erythrocytes. The reaction with succinate was repeated in the presence of 100 units/mL SOD.

### 4.8 Cell culture

Human epithelial HeLa cells, murine fibroblast C2C12 cells and human prostatic adenocarcinoma PC-3 cells were obtained from American Type Culture Collection (ATCC). HeLa and C2C12 cells were cultured in DMEM (Gibco) supplemented with 10% Fetal Bovine Serum (FBS), 100 units/mL penicillin and 100 mg/mL streptomycin. PC-3 cells were cultured in Ham's F12 Nutrient Mixture (Gibco) supplemented with 10% Fetal Bovine Serum, 100 units/mL penicillin and 100 mg/mL streptomycin. The pWPXLd-ires mCherry-TOMM20-N-10-Puro lentiviral vector was created by insertion of cloning mCherry-TOMM20-N-10 (Addgene #55146) within the *Pme*I/BamH1 MCS of thepWPXLd-ires-Puro lentiviral vector (obtained from Massimo Zeviani, Mitochondrial Biology Unit, University of Cambridge, UK) by Infusion cloning. Lentiviral particles were generated in HEK293T cells via co-transfection of the target vector together with packaging psPAX2 (Addgene #12260) and envelope pMD2.G (Addgene #12259) vectors. Viral particles were collected 24 h after transfection and WT HeLa cells were stably transduced with pWPXLd-ires-mCherry-TOMM20-N-10-puromycin lentiviral vector using 8 μg/μL of polybrene transfection reagent (Sigma-Aldrich, TR-1003). Cells expressing pWPXLd-ires-mCherry-TOMM20-N-10-puromycin vectors were selected for puromycin resistance 24 h after transduction. Mitochondrial localization of mCherry signal was validated by confocal microscopy.

### 4.9 Live-cell confocal microscopy

For MitoMenOH **5** and MitoMenOAc **6** experiments, C2C12 cells were seeded at 10,000 cells per well in an eight-well chambered coverslip (Ibidi) overnight and then treated with 3 μM menadione **25**, MitoMenOH **5** or MitoMenOAc **6** for 15 min. The media was removed and the cells were incubated in 5 μM MitoNeoD and 5 nM MitoTracker Deep Red FM for 20 min. The media was then replaced with phenol red-free DMEM (Gibco). All images were acquired using a Zyla 4.2 PLUS sCMOS camera attached to an Andor DragonFly 500 confocal spinning disk mounted on a Nikon Eclipse TiE microscope using a CFI Plan Apochormat lambda × 100 oil immersion objective. Images were acquired at physiological conditions (37 °C, 5% CO_2_ and 95% relative humidity) within a H301–K-FRAME stage top incubator (Okolab), using the Fusion user interface (Andor). The following laser settings were used: 500 ms exposure, 50% laser intensity and excitation/emission 561/620 nm for MitoNeoOH and 500 ms exposure, 50% laser intensity and excitation/emission 637/700 nm for MitoTracker Deep Red FM. Ten Z-stacks of 0.2 μ m were acquired. The absolute intensity (A.U.) of MitoNeoOH was measured by randomly selecting 25-40 regions of interest (ROIs) (15 × 15 μ m) per biological replicate. The image processing workflow was automated using the Fiji ImageJ macro scripts as previously described [55]. Mitochondrial morphology was assessed in ROIs of C2C12 cells stained with MitoTracker Deep Red from images obtained by live-cell confocal microscopy. Morphological features were analysed and quantified using an automated processing workflow called MitoMAPR [56]. In this process, ROIs were converted to binary images, which were skeletonized and processed with the AnalyzeSkeleton plugin. For MitoHCoum2 **3** experiments, HeLa cells stably expressing Tomm20-mCherry were seeded into eight-well chambered coverslips (Ibidi) at 15,000 cells per well and cultured overnight. Cells were pre-incubated with DMSO, 10 μM BAM15, or 2 μM FCCP for 20 min before imaging. Following treatment, cells were washed three times with warm serum-free medium and incubated for a further 10 min in serum-free medium. MitoHCoum2 (3 μM) was added at 0 s *in situ*, and images were acquired every 10 s using a 100 × objective as described above. The fluorescence intensity of 7-hydroxycoumarin over time, normalised to Tomm20-mCherry fluorescence, was quantified in FIJI using the ‘Plot Z-axis Profile’ function from ∼4 regions of interest (15 × 15 μm) per biological replicate.

### 4.10 LDH assay

C2C12 and PC-3 cells were seeded at 10,000 cells per well in 96 well plates and left to adhere overnight.The media was removed and the cells were washed with PBS. 200 μL DMEM without phenol red (Gibco) and without FBS but containing different concentrations of menadione **25**, MitoHCoum2 **4**, MitoMenOH **5** or MitoMenOAc **6** were added and left to incubate at 37 °C for 3 h. The volume of ethanol was kept at 2 μL across all experiments. After 3 h, 100 μL of media was removed and added to another 96-well plate, where the LDH activity was measured using the Roche LDH cytotoxicity kit. Cell medium was used as a blank to correct for background absorbance. LDH release was normalised for a 1% v/v Triton X-100 positive control, which results in 100% cell death.

### 4.11 Cell viability by flow cytometry

C2C12 cells were seeded at 500,000 cells per well in 6 well plates and left to adhere overnight. The media was replaced with 1 mL DMEM (Gibco) without FBS containing 20 μM of either menadione, MitoHCoum2 **4**, both menadione and MitoHCoum2 **4**, MitoMenOH **5** or MitoMenOAc **6**, and the cells were incubated at 37 °C for 3 h. The volume of ethanol was kept at 2 μL across all experiments. The culture media was collected, and the remaining cells were harvested by trypsinisation. The combined media and cells were centrifuged at 1000×*g*. Cell viability was measured using the Dead Cell Apoptosis Kit with Annexin V FITC and Propidium Iodide (PI) for flow cytometry (Invitrogen). The pelleted cells were resuspended in 500 μL of 1X annexin binding buffer. 50 μL of resuspended cells was added to 50 μL of 1X Annexin binding buffer containing Annexin V FITC and PI, and incubated at room temperature in the dark for 15 min. The samples were diluted with 400 μL of 1X Annexin binding buffer and kept on ice for analysis using a BD LSRFortessa Cell Analyser. Cells were identified as follows: live cells were negative for Annexin V FITC and PI, apoptotic cells were positive for Annexin V FITC and negative for PI, and dead cells were positive for both Annexin V FITC and PI. Data were analysed using FlowJo software v.10.10.0 (FlowJo). Gating was kept consistent across all samples, except for those treated with MitoMenOAc **6**, where the Annexin V FITC gate was adjusted to account for a shift in cell population fluorescence (Fig. S7A and B).

### 4.12 Statistical analysis

Microsoft Excel and GraphPad Prism 10 were used for statistical analysis and graph production. Statistical comparison between two columns was carried out using an unpaired *t*-test. Statistical comparison between three or more columns was carried out using a one-way ANOVA test. Statistical comparison between three or more groups was carried out using a two-way ANOVA test. The number of biological replicates (n) and statistical values can be found in each figure legend. For *in vitro* experiments without biological tissue, n = independent experiments, whereas for isolated mitochondria and cell experiments, n = tissue from individual animals or cells from different passages, respectively. ∗p < 0.05, ∗∗p < 0.01, ∗∗∗p < 0.001, ∗∗∗∗p < 0.0001.

## CRediT authorship contribution statement

**Patrick A. Cardwell:** Conceptualization, Data curation, Formal analysis, Investigation, Methodology, Project administration, Writing – original draft, Writing – review & editing. **Alva M. Casey:** Data curation, Formal analysis, Investigation, Methodology, Writing – review & editing. **Suvagata Roy Chowdhury:** Data curation, Formal analysis, Investigation, Methodology, Writing – review & editing. **Eloïse Marques:** Data curation, Formal analysis, Investigation, Methodology, Software, Writing – review & editing. **Chak Shun Yu:** Data curation, Formal analysis, Investigation, Methodology, Writing – review & editing. **Rebecca L. Taig:** Investigation, Writing – review & editing. **Stuart T. Caldwell:** Data curation, Formal analysis, Investigation, Methodology, Supervision, Writing – review & editing. **Julien Prudent:** Resources, Writing – review & editing. **Michael P. Murphy:** Conceptualization, Funding acquisition, Methodology, Project administration, Resources, Writing – review & editing. **Richard C. Hartley:** Conceptualization, Funding acquisition, Methodology, Project administration, Resources, Supervision, Writing – review & editing.

## Declaration of competing interest

The authors declare the following financial interests/personal relationships which may be considered as potential competing interests: Richard C. Hartley reports financial support was provided by Wellcome Trust. Patrick A. Cardwell reports financial support was provided by Engineering and Physical Sciences Research Council. Michael P. Murphy reports financial support was provided by UKRI Medical Research Council. Michael P. Murphy reports financial support was provided by Wellcome Trust. If there are other authors, they declare that they have no known competing financial interests or personal relationships that could have appeared to influence the work reported in this paper.

## Data Availability

The raw NMR, fluorescence and kinetics data are available here http://dx.doi.org/10.5525/gla.researchdata.2122.
