# Supplementary material for: Dual-action mitochondria-targeted prodrugs that both deplete mitochondrial glutathione and deliver a toxic payload to the matrix
Source: Eur J Med Chem. 2026 Apr 15;308:118706. doi: 10.1016/j.ejmech.2026.118706 (PMC13201212; doi:10.1016/j.ejmech.2026.118706)
Supplement: Multimedia component 1 [file mmc1.docx]

Supporting Information

**Dual-action mitochondria-targeted prodrugs that both deplete mitochondrial glutathione and deliver a toxic payload to the matrix**

**Patrick A. Cardwell^a,b^, Alva M. Casey^b^, Suvagata Roy Chowdhury^b^, Eloïse Marques^b^, Chak Shun Yu^b^, Rebecca L. Taig^a^, Stuart T. Caldwell^a^, Julien Prudent^b^, Michael P. Murphy^b,c,*.^ Richard C. Hartley^a,*^**

^a^*School of Chemistry, Joseph Black Building, University Avenue, University of Glasgow, Glasgow G12 8QQ, UK*

^b^*MRC Mitochondrial Biology Unit, University of Cambridge, Cambridge Biomedical Campus, Cambridge CB2 0XY, UK*

^c^*Department of Medicine, University of Cambridge, CB2 0QQ, UK*

**Corresponding authors –* [*mpm37@cam.ac.uk*](mailto:mpm37@cam.ac.uk)*,* [*richard.hartley@glasgow.ac.uk*](mailto:richard.hartley@glasgow.ac.uk)

Contents

[Additional Synthesis 3](#_Toc217045432)

[Reaction between menadione and GSH analysed by LC-MS/MS 4](#_Toc217045433)

[Supplementary Figures 5](#_Toc217045434)

[References 11](#_Toc217045435)

[NMR spectra of compounds 12](#_Toc217045436)

# Additional Synthesis

**Scheme 1.** Synthesis of 7-aminocoumarin. a: Bromoacetamide, K_2_CO_3_, MeCN, 50 °C, 16 h. b: (i) Cs_2_CO_3_, DMF, 70 °C, 24 h. (ii) HCl, EtOH, 90 °C, 5 h.

**2‐[(2’‐Oxo‐2H‐chromen‐7’‐yl)oxy]acetamide**

Adapting the procedure of Lippe *et al* [1], bromoacetamide (1.02 g, 7.40 mmol, 1.20 eq.) and K_2_CO_3_ (1.02 g, 7.40 mmol, 1.00 eq.) were added to a solution of 7-hydroxycoumarin (1.00 g, 0.62 mmol, 1.00 eq.) in anhydrous acetonitrile (40 mL) and the reaction was stirred at 50 °C for 16 h under argon. The reaction was allowed to cool to room temperature and the solvent was removed under reduced pressure. The crude material was washed with water (2 x 40 mL) and filtered under vacuum to afford 2‐[(2’‐oxo‐2H‐chromen‐7’‐yl)oxy]acetamide as a white solid (1.26 g, 93%). Mp: 219 - 220 °C. Ʋ_max_ (ATR): 3437 (N-H), 1716 (C=O), 1674 (C=O) cm^-1^. ^1^H NMR (400 MHz, DMSO): δ_H_ 7.99 (1H, d, *J* = 9.5 Hz, H-4’), 7.65 (1H, d, *J* = 8.5 Hz, H-5’), 7.61 (1H, s, NH), 7.43 (1H, s), 6.99 (1H, dd, *J* = 8.5, 2.5 Hz, H-6’), 6.96 (1H, d, *J* = 2.4 Hz, H-8’), 6.31 (1H, d, *J* = 9.5 Hz, H-3’), 4.56 (2H, s, CH_2_). ^13^C NMR (101 MHz, DMSO): δ_C_ 169.16 (C), 160.83 (C), 160.18 (C), 155.13 (C), 144.24 (CH), 129.46 (CH), 112.80 (CH), 112.76 (C), 112.73 (CH), 101.65 (CH), 66.92 (CH_2_). HRMS (APCI^+^): C_12_H_10_NO_4_ requires 220.0604 found 220.0605 (M + H)^+^.

**7‐Aminocoumarin**

Adapting the procedure of Lippe *et al* [1], Cs_2_CO_3_ (2.25 g, 6.92 mmol, 1.20 eq.) was added to a solution of 2‐[(2’‐oxo‐2H‐chromen‐7’‐yl)oxy]acetamide (1.26 g, 5.77 mmol, 1.00 eq.) in anhydrous dimethylformamide (58 mL) and the reaction was stirred at 70 °C for 24 h under argon. The reaction was allowed to cool to room temperature and the solvent was removed under reduced pressure. The solid residue was redissolved in ethanol (58 mL) and HCl (18 mL, 6 M). The reaction was stirred at 90 °C for 5 h under argon. The solvent was removed under reduced pressure and the solid residue was redissolved in dichloromethane (50 mL) and washed with a saturated solution of NaHCO_3_ (50 mL). The organic layer was separated, and the aqueous layer was extracted with dichloromethane (2 x 40 mL). The organic layers were combined, dried with MgSO_4_ and the solvent was removed under reduced pressure. The crude material was purified by column chromatography [SiO_2_, dichloromethane: methanol 0-10%] to afford 7-aminocoumarin as an off-white solid (325 mg, 35%). Mp: 202 - 204 °C. ^1^H NMR (400 MHz, (CD_3_)_2_CO)): δ_H_ 7.72 (1H, d, *J* = 9.4 Hz, H-4), 7.31 (1H, d, *J* = 8.4 Hz, H-5), 6.64 (1H, dd, *J* = 8.4, 2.1 Hz, H-6), 6.52 (1H, d, *J* = 2.1 Hz, H-8), 5.96 (1H, d, *J* = 9.4 Hz, H-3), 5.62 (2H, s, NH_2_). ^13^C NMR (101 MHz, (CD_3_)_2_CO): δ_C_ 161.58 (C), 157.63 (C), 153.81 (C), 153.75 (C), 144.93 (CH), 130.14 (CH), 112.31 (CH), 110.09 (CH), 100.23 (CH). HRMS (APCI^+^): C_9_H_8_NO_2_ requires 162.0550 found 162.0554 (M + H)^+^. The ^1^H and ^13^C NMR data is in broad agreement with that reported in the literature in DMSO [2].

# Reaction between menadione and GSH analysed by LC-MS/MS

MS¹ and product ion scans were performed using flow-injection analysis (FIA) on a Shimadzu 8060 triple quadrupole mass spectrometer (Shimadzu, UK). Menadione standard and samples obtained after the reaction between GSH and menadione were injected (2 µL) and mass spectra were acquired over an m/z range of 100–1000. Chromatographic separation was performed on an Atlantis Premier BEH Z-HILIC column (1.7 μm, 2.1 × 150 mm; Waters). Mobile phase A consisted of 15 mM ammonium acetate with 0.1% ammonium, and mobile phase B was 100% MeOH. The flow rate was 0.1 mL/min, and the injection volume was 10 to 20 µL. HPLC was performed on an LC-30AD system with a flow rate of 0.1 mL/min. Samples were injected at 0.5 min using a SIL-30AC autosampler. The total run time was 10 min with the following gradient: 0–10 min: 80% B isocratic elution. No internal standard was used. **Source conditions were as follows:** Nebulizing gas: 3 L/min; Heating gas: 10 L/min; Interface temperature: 400 °C; DL temperature: 245 °C; Heat block temperature: 400 °C; Drying gas: 10 L/min. Mass ions were detected using positive ion mode as follows:

***Menadione GS-Conjugate***

- 479.0 → 331.9: Dwell 100; Q1 -15 V; CE -18; Q3 -11 V
- 479.0 → 173.15: Dwell 100; Q1 -25 V; CE -49; Q3 -17 V
- 479.0 → 231.3: Dwell 100; Q1 -26 V; CE -27; Q3 -36 V
- 479.0 → 403.95: Dwell 100; Q1 -25 V; CE -21; Q3 -28 V

*Menadione*

- 172.95 → 127.05: Dwell 100; Q1 -18 V; CE -23; Q3 -25 V
- 172.95 → 117.0: Dwell 100; Q1 -19 V; CE -19; Q3 -19 V
- 172.95 → 77.2: Dwell 100; Q1 -20 V; CE -35; Q3 -13 V
- 172.95 → 155.05: Dwell 100; Q1 -21 V; CE -19; Q3 -15 V

# Supplementary Figures

**Figure S1.** Fluorescence calibration curves of (A) 7-hydroxycoumarin **9**, (B) ACoum1 **12** and (C) ACoum2 **13** at pH 8.0 excited at 342 nm and emission measured at 342 nm using a Horiba Duetta spectrometer.

**Figure S2.** Initial rate graphs of the reactions of 10 µM prodrug compounds with or without GSH at pH 8.0, 30 °C. Data are means ± SD, N = 3. (A) HCoum1 **7**; (B) HCoum2 **8**; (C) MitoACoum1 **10** + 2 mM GSH; (D) MitoACoum2 **11** + 2 mM GSH; (E) MitoHCoum1 **3** + 10 mM GSH; (F) MitoHCoum2 **4** + 10 mM GSH. Initial rates in µM/s were divided by 10 µM compound to obtain the first or pseudo-first order rate constant, the latter of which was divided by the concentration of GSH to obtain the second-order rate constant.

**Figure S3.** Reaction of menadione (5 µM) with GSH (5 µM) at pH 8.0 analysed by LC-MS/MS.

**Figure S4**. Fluorescence calibration curve of 7-hydroxycoumarin **9** at pH 8.0 excited at 342 nm and emission measured at 342 nm using a CLARIOstar Plus plate-reader.

**Figure S5.** Prodrugs incubated with isolated rat liver mitochondria (RLM) or rat heart mitochondria (RHM) associate with the pellet, analysed by RP-HPLC. (A) MitoHCoum2 **4** (5 µM) incubated with RLM (1 mg/mL), glutamate (10 mM) and malate (10 mM) ± FCCP (1 µM). (B) MitoMenOH **5** (5 µM) incubated with RLM (1 mg/mL), glutamate (10 mM) and malate (10 mM) ± FCCP (1 µM). (C) MitoMenOAc **6** (5 µM) incubated with RLM (1 mg/mL), glutamate (10 mM) and malate (10 mM) ± FCCP (1 µM). MitoMenOAc **6** is primarily converted to MitoMenOH **5** by esterase-catalysed hydrolysis. (D) MitoMenOAc **6** (5 µM) incubated with RHM (1 mg/mL), glutamate (10 mM) and malate (10 mM) ± FCCP (1 µM). Data are means ± SEM, N ≥ 3. p values were calculated using an unpaired t test for experiments (A), (B) and (D), and using a two-way ANOVA test for experiment (C). *p < 0.05, **p < 0.01.

**Figure S6**. MitoHCoum2 (10 µM) incubated with DMEM media ± FBS and release of 7-hydroxycoumarin analysed by fluorescence.

**Figure S7.** (A) Gating strategy for C2C12s as analysed by flow cytometry. (B) Representative gated images for each condition. (C) Live cells (left) and combined apoptotic and dead cells (right) after incubation with 20 µM of compound for 3 h.

# References

1. Lippe, D. S.; Elghawy, O.; Zucker, A. M.; Yanagawa, E. S. K.; Mathews, E.; Ahmed, Y. G.; D'Elia, P. N.; Bimson, S.; Walvoord, R. R., Synthesis of 7-Aminocoumarins from 7-Hydroxycoumarins via Amide Smiles Rearrangement. *ACS Omega* **2022,** *7* (39), 35269-35279.

2. Yu, J.; Wang, Y.; Zhang, P.; Wu, J., Direct Amination of Phenols under Metal-Free Conditions. *Synlett* **2013,** *24* (11), 1448-1454.

# NMR spectra of compounds
